# Supplementary material for: CNN2 silencing inhibits colorectal cancer development through promoting ubiquitination of EGR1
Source: Life Sci Alliance. 2023 May 15;6(7):e202201639. doi: 10.26508/lsa.202201639 (PMC10185810; doi:10.26508/lsa.202201639)
Supplement: Supplementary file 1 [file LSA-2022-01639_SdataF1_F2.6_F3_F4_F5.pptx]

## Slide 1
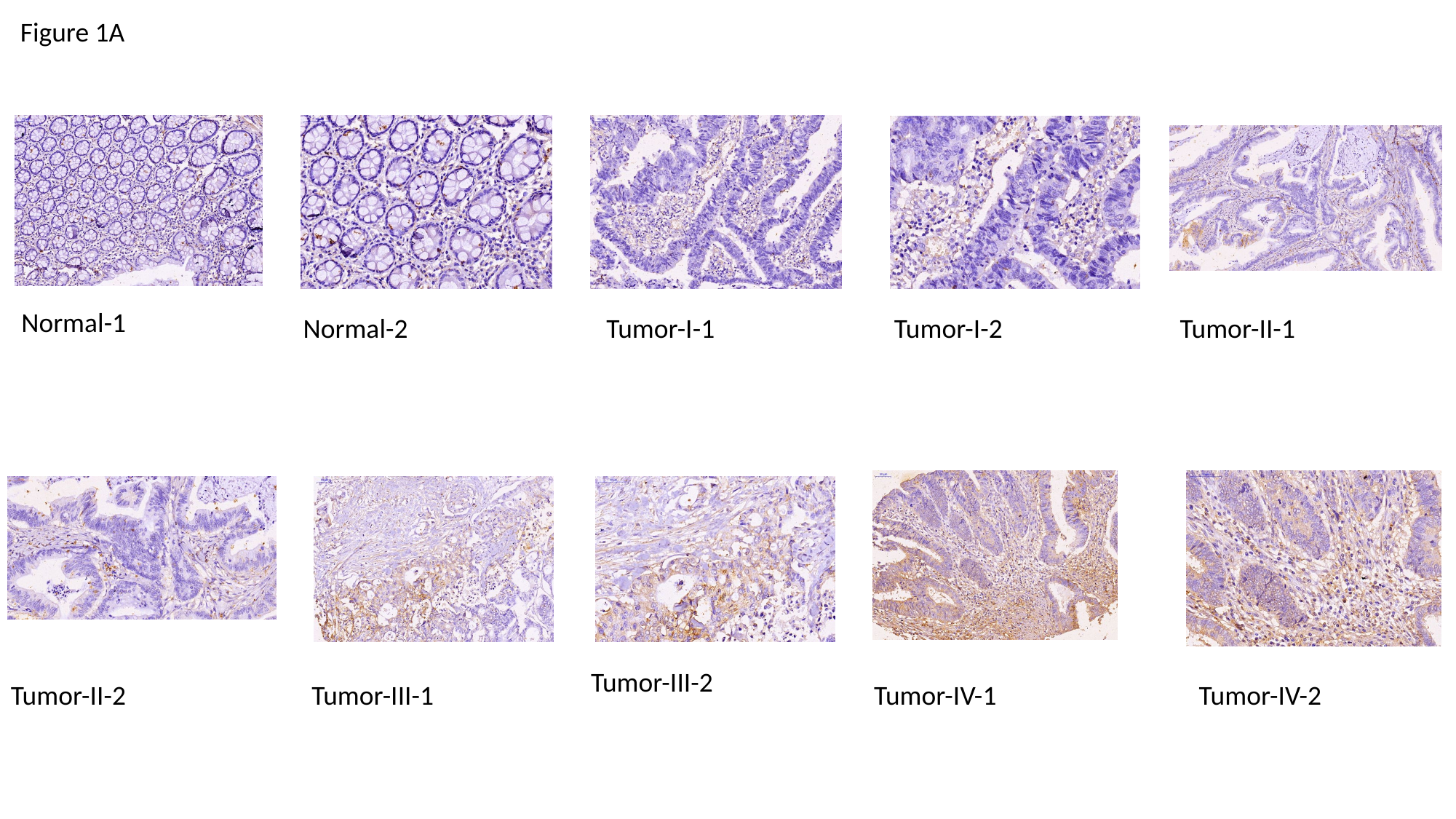

Figure 1A
Normal-1
Normal-2
Tumor-I-1
Tumor-I-2
Tumor-II-1
Tumor-III-2
Tumor-II-2
Tumor-III-1
Tumor-IV-1
Tumor-IV-2

## Slide 2
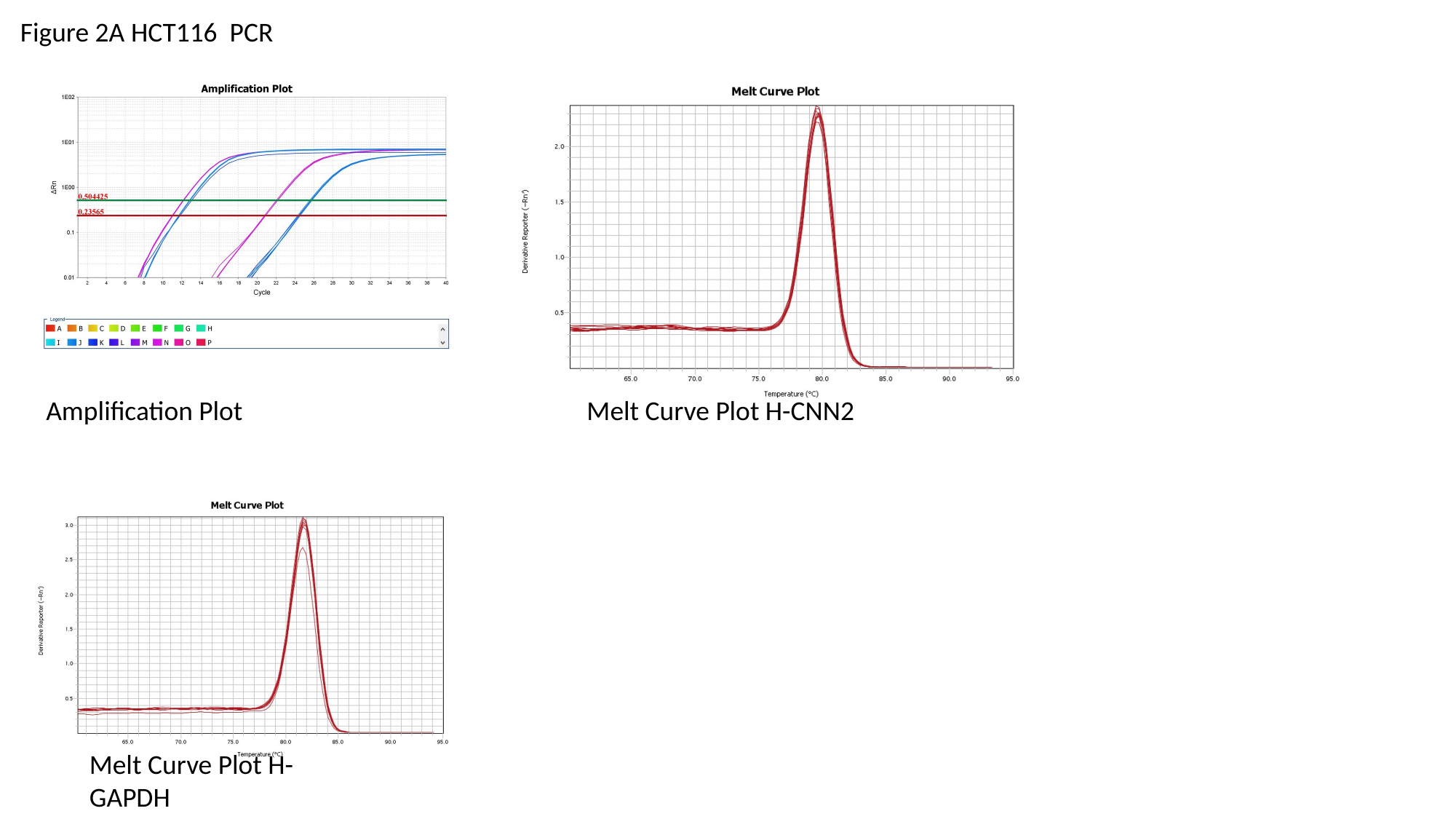

Figure 2A HCT116 PCR
Amplification Plot
Melt Curve Plot H-CNN2
Melt Curve Plot H-GAPDH

## Slide 3
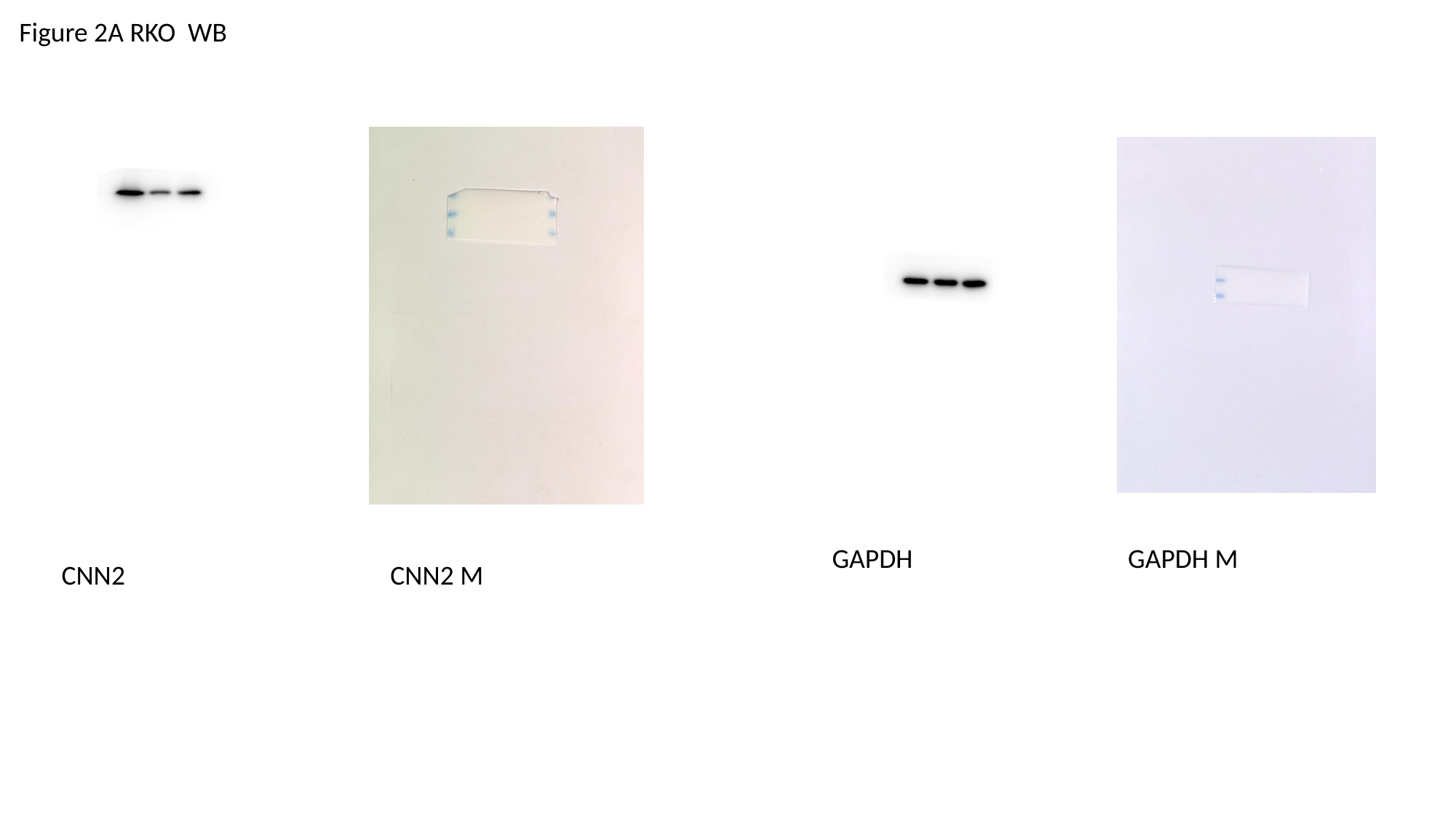

Figure 2A RKO WB
GAPDH
GAPDH M
CNN2
CNN2 M

## Slide 4
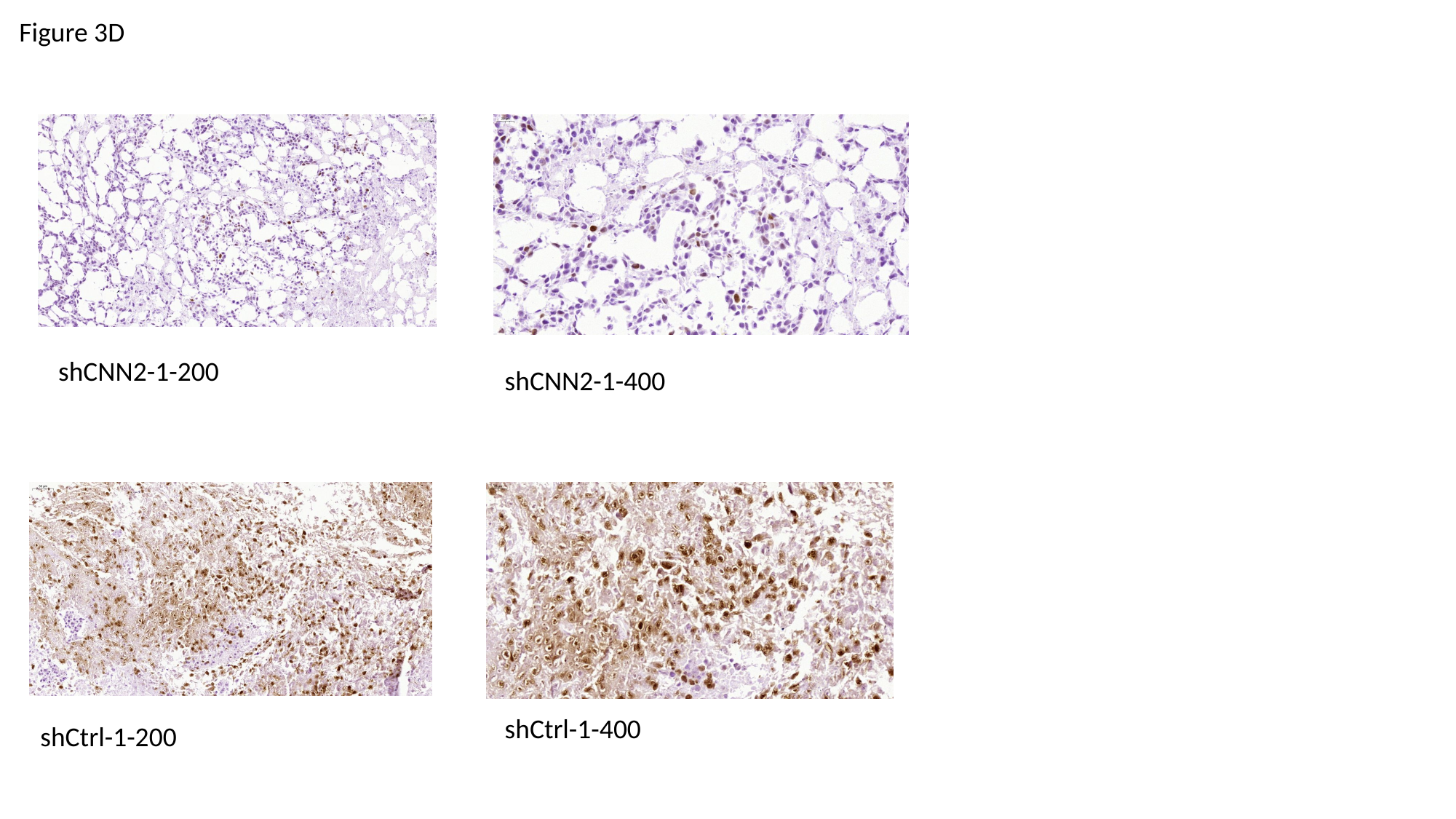

Figure 3D
shCNN2-1-200
shCNN2-1-400
shCtrl-1-400
shCtrl-1-200

## Slide 5
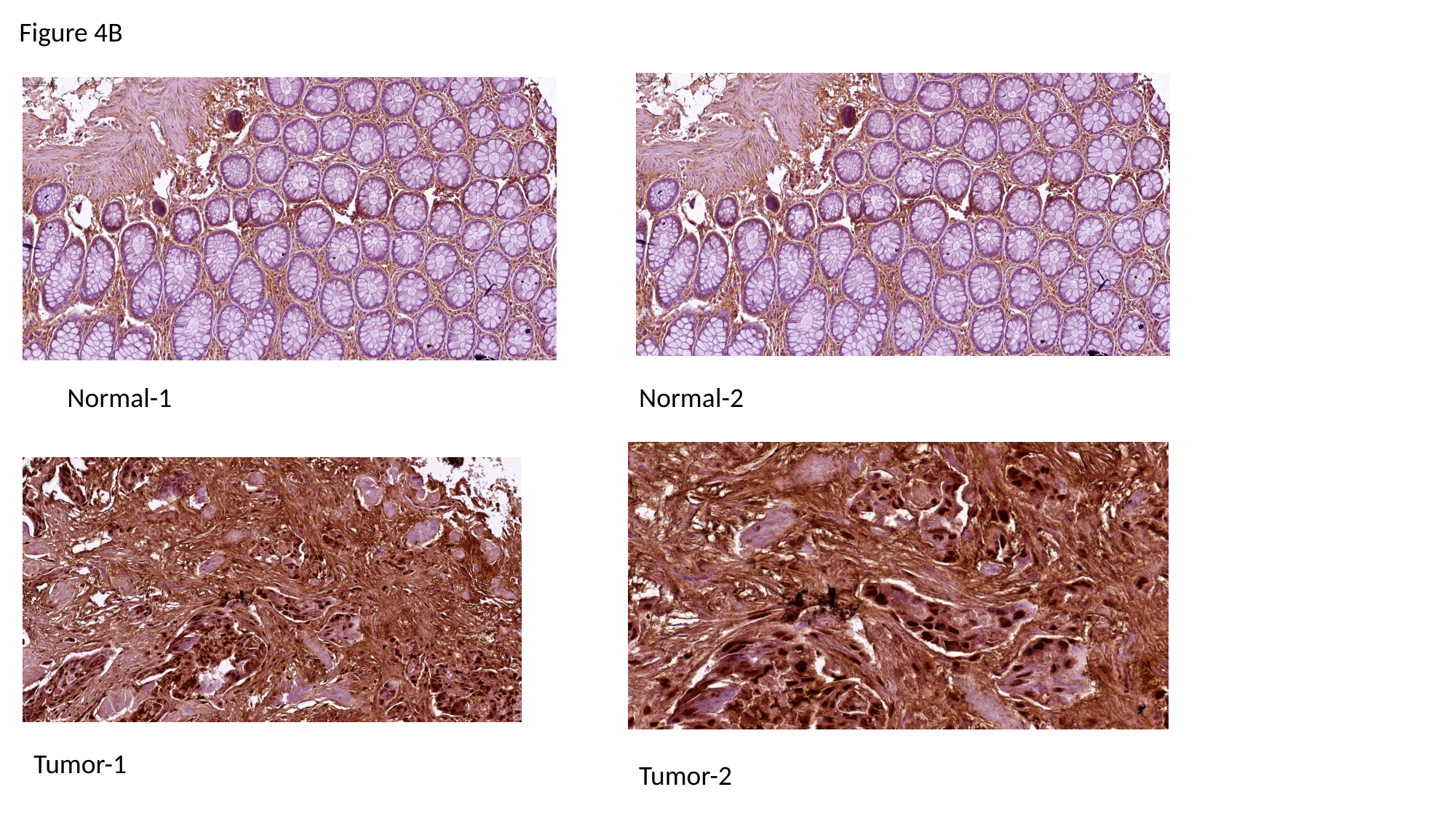

Figure 4B
Normal-1
Normal-2
Tumor-1
Tumor-2

## Slide 6
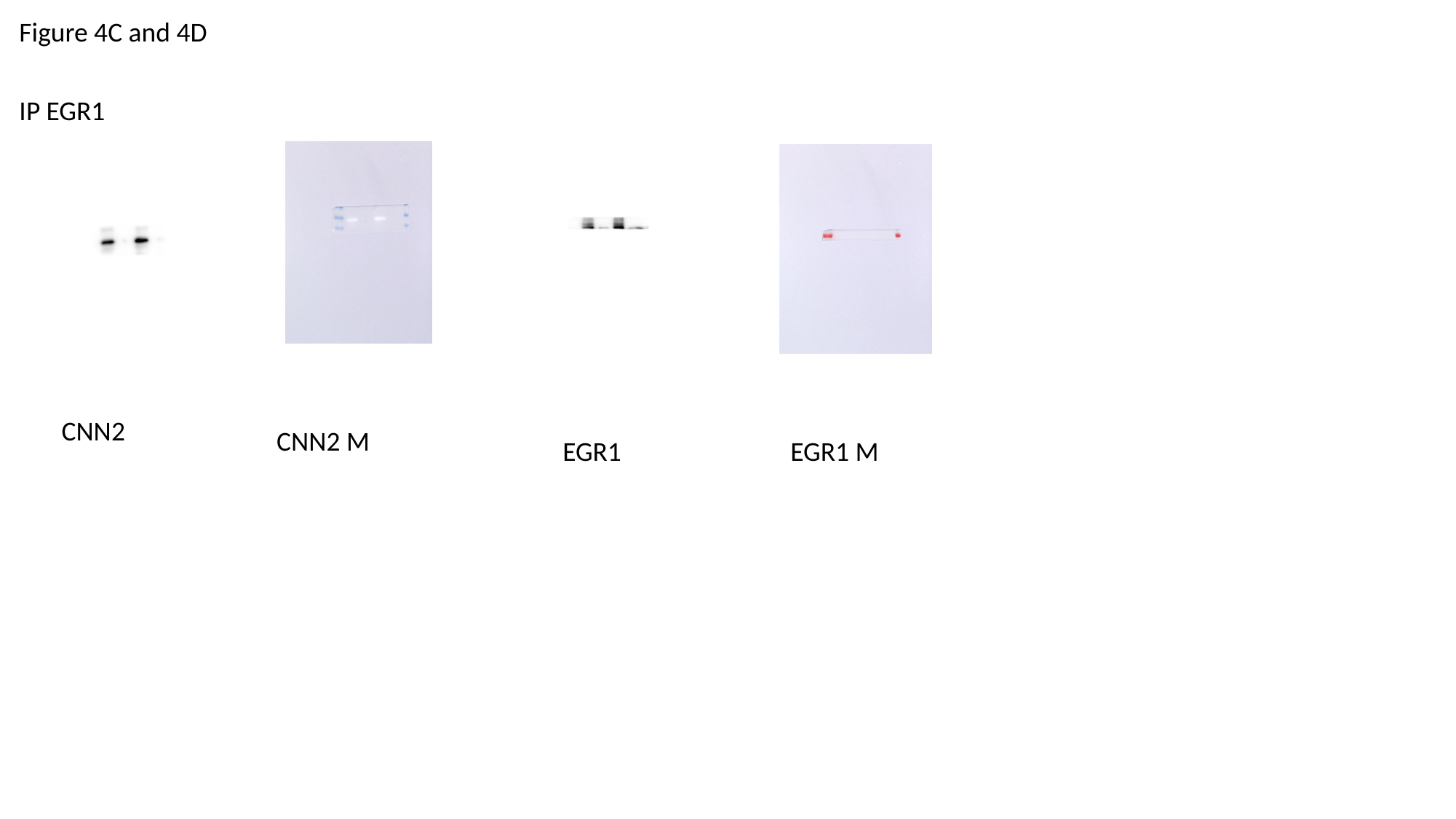

Figure 4C and 4D
IP EGR1
CNN2
CNN2 M
EGR1
EGR1 M

## Slide 7
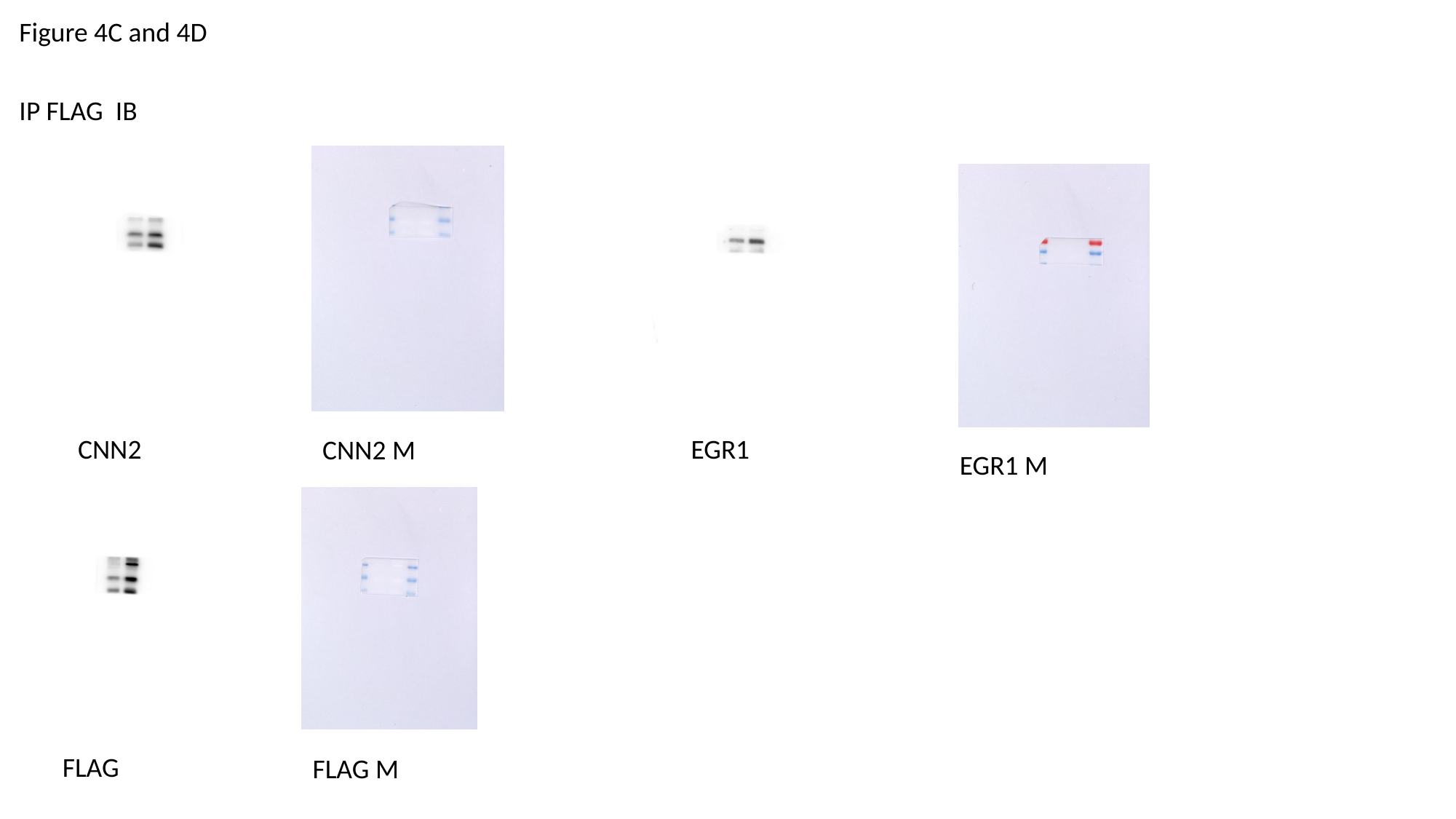

Figure 4C and 4D
IP FLAG IB
CNN2
EGR1
CNN2 M
EGR1 M
FLAG
FLAG M

## Slide 8
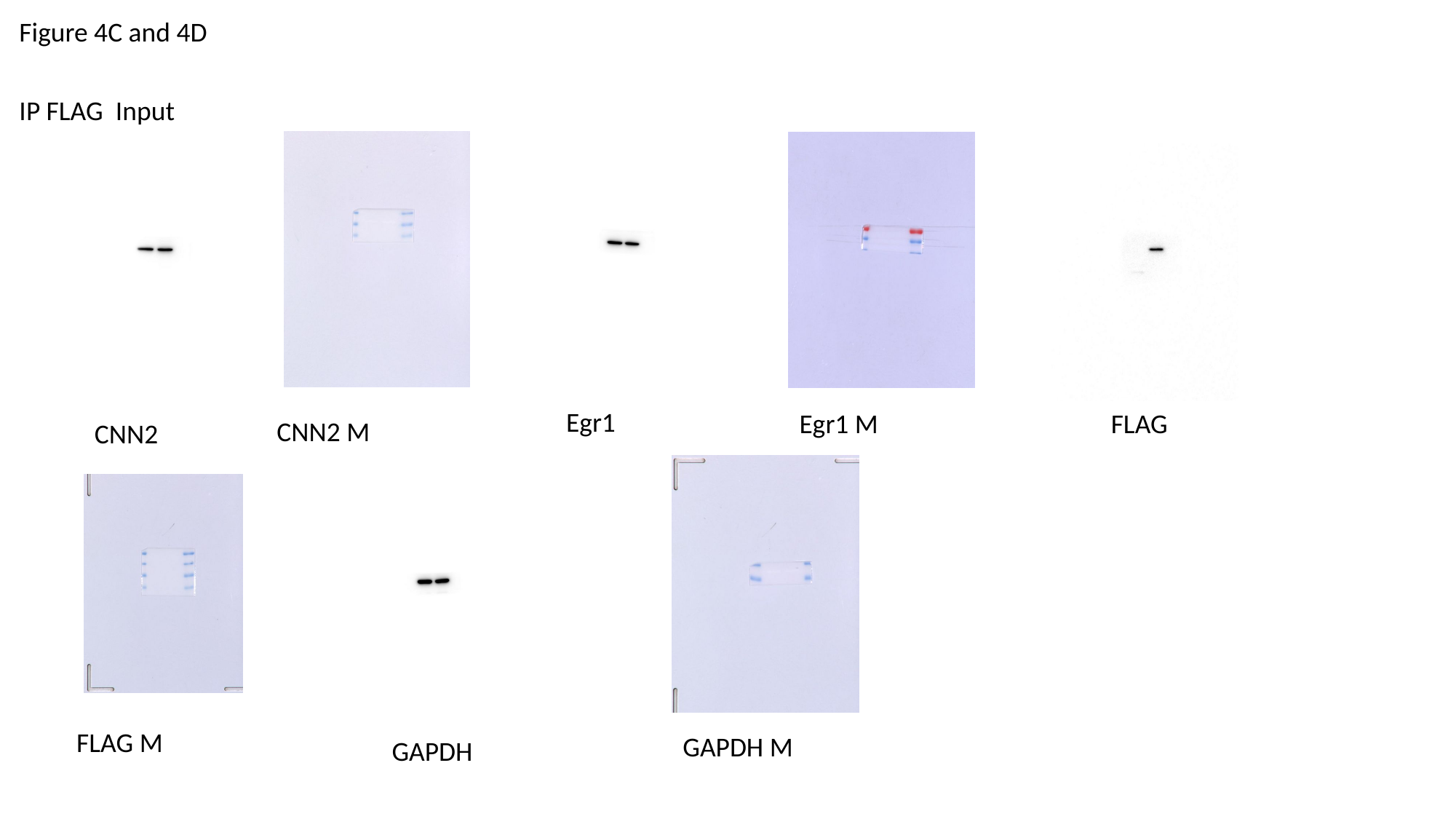

Figure 4C and 4D
IP FLAG Input
Egr1
Egr1 M
FLAG
CNN2 M
CNN2
FLAG M
GAPDH M
GAPDH

## Slide 9
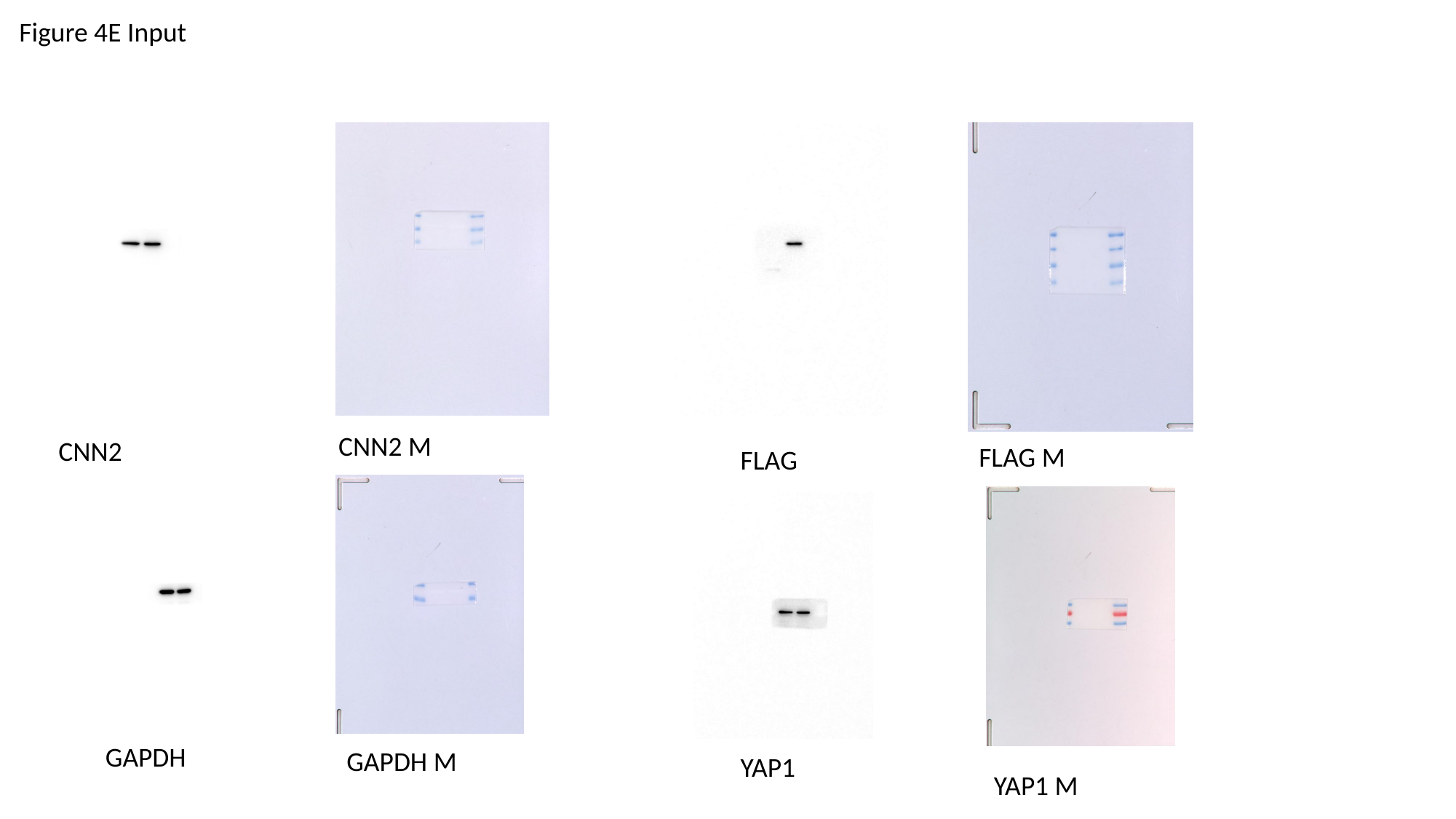

Figure 4E Input
CNN2 M
CNN2
FLAG M
FLAG
GAPDH
GAPDH M
YAP1
YAP1 M

## Slide 10
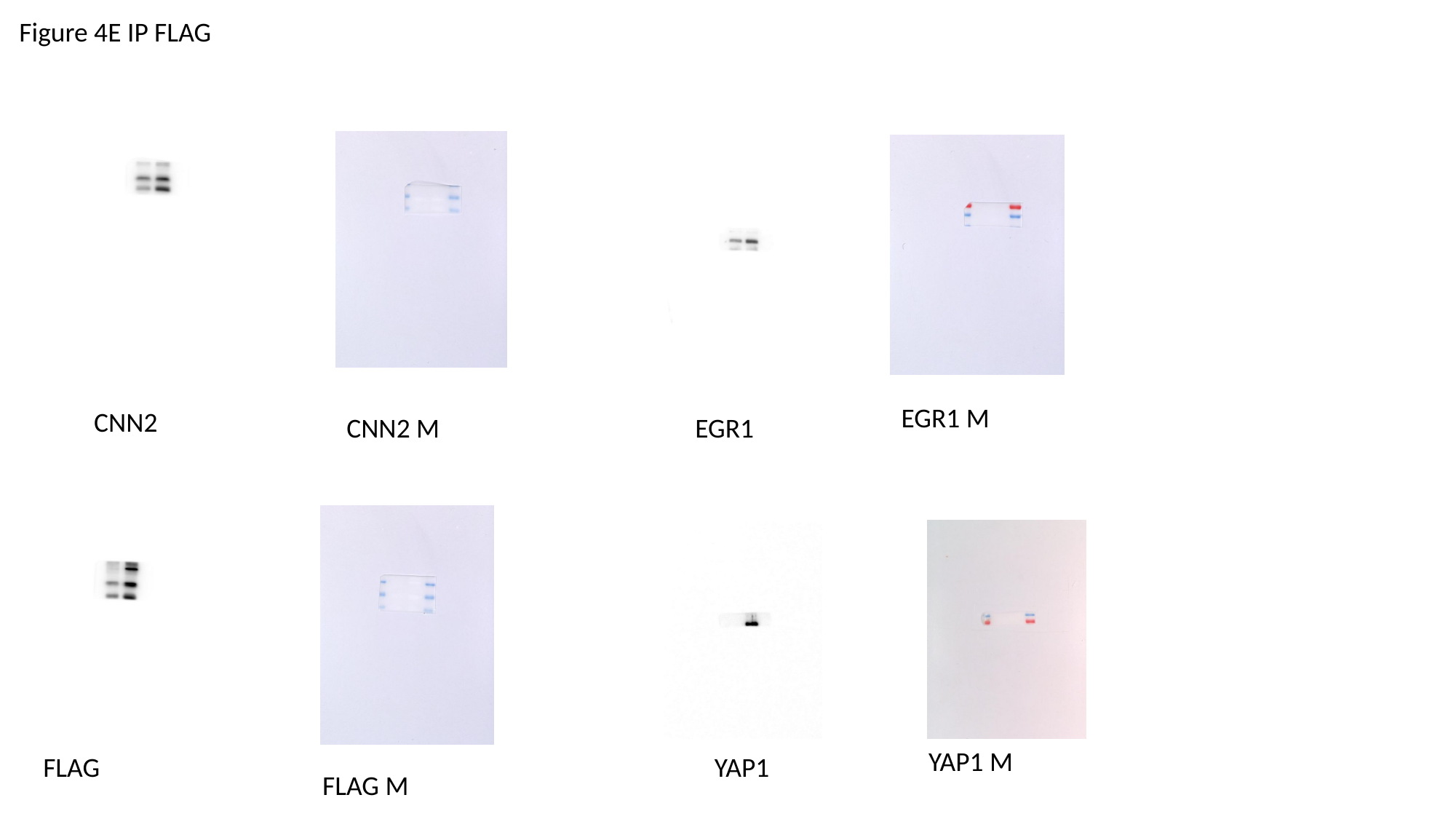

Figure 4E IP FLAG
EGR1 M
CNN2
CNN2 M
EGR1
YAP1 M
FLAG
YAP1
FLAG M

## Slide 11
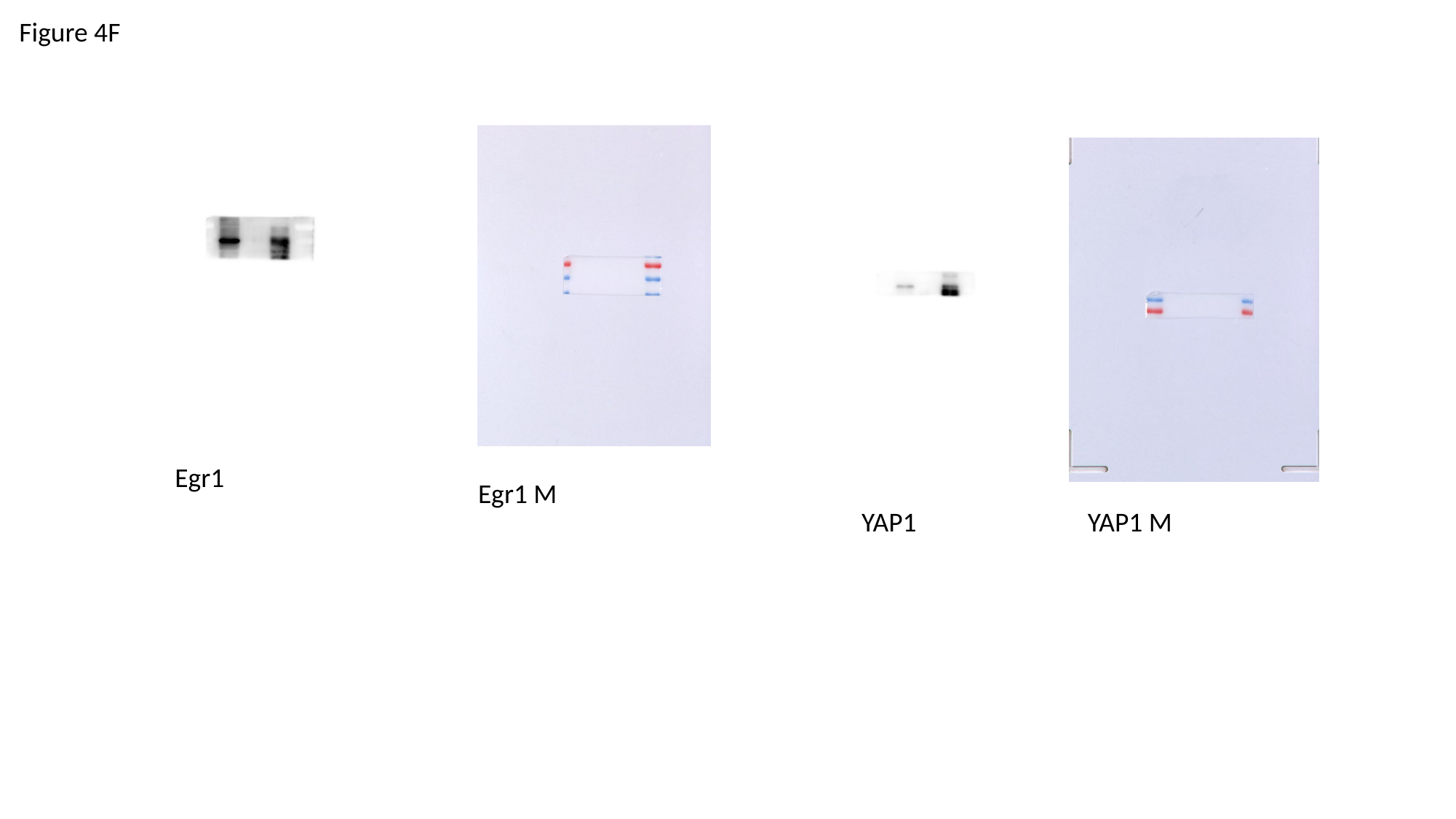

Figure 4F
Egr1
Egr1 M
YAP1
YAP1 M

## Slide 12
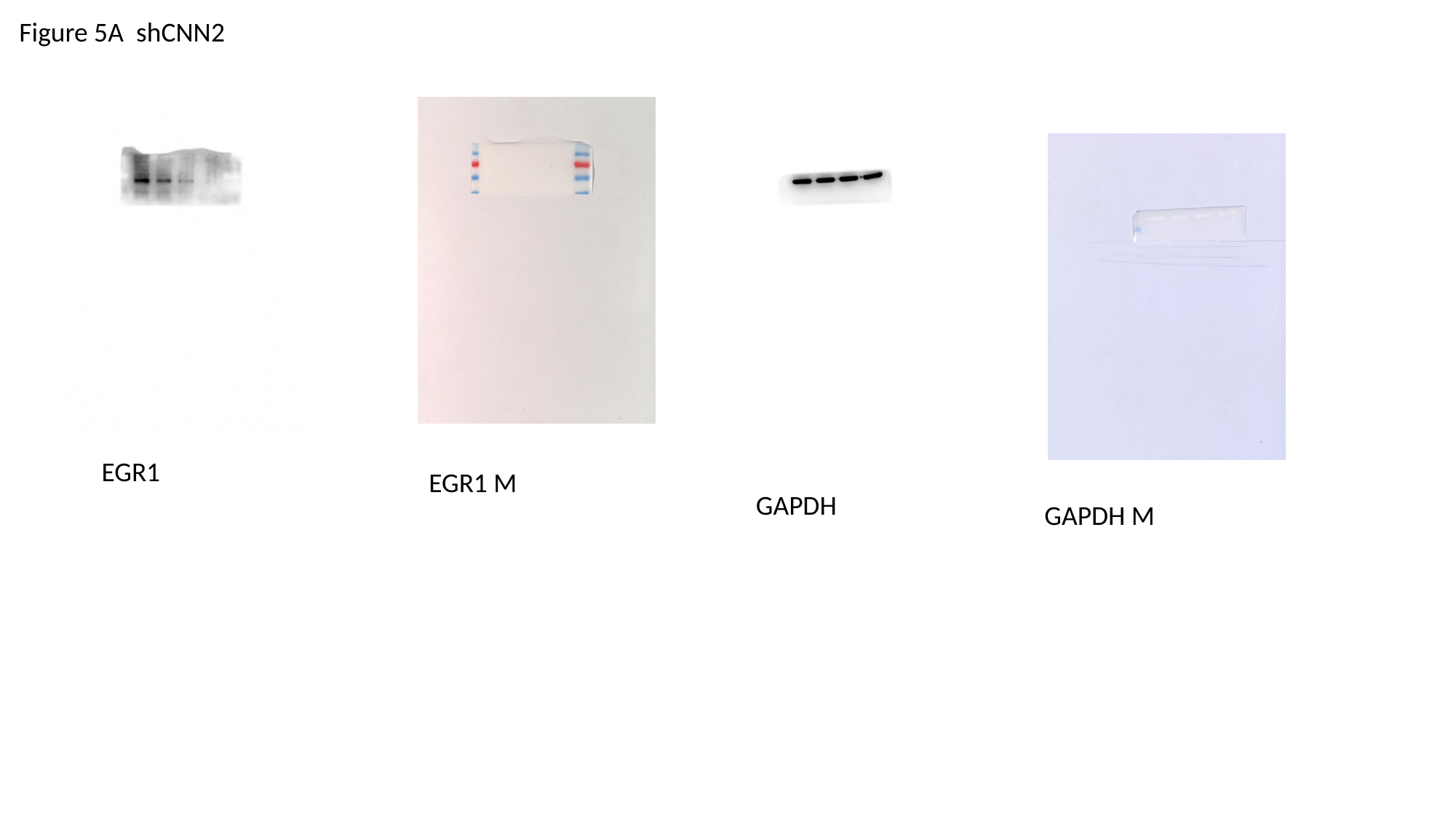

Figure 5A shCNN2
EGR1
EGR1 M
GAPDH
GAPDH M

## Slide 13
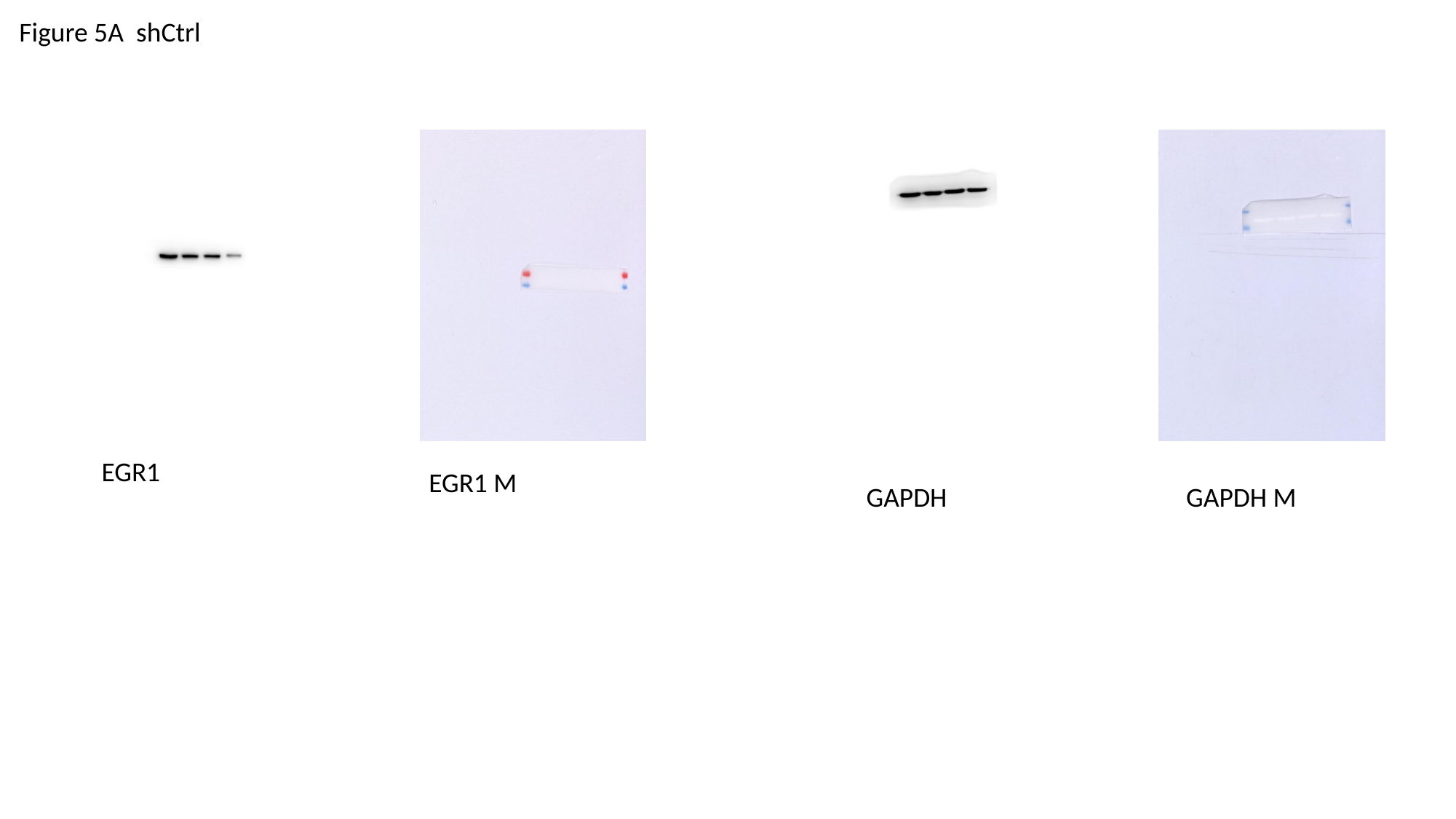

Figure 5A shCtrl
EGR1
EGR1 M
GAPDH
GAPDH M

## Slide 14
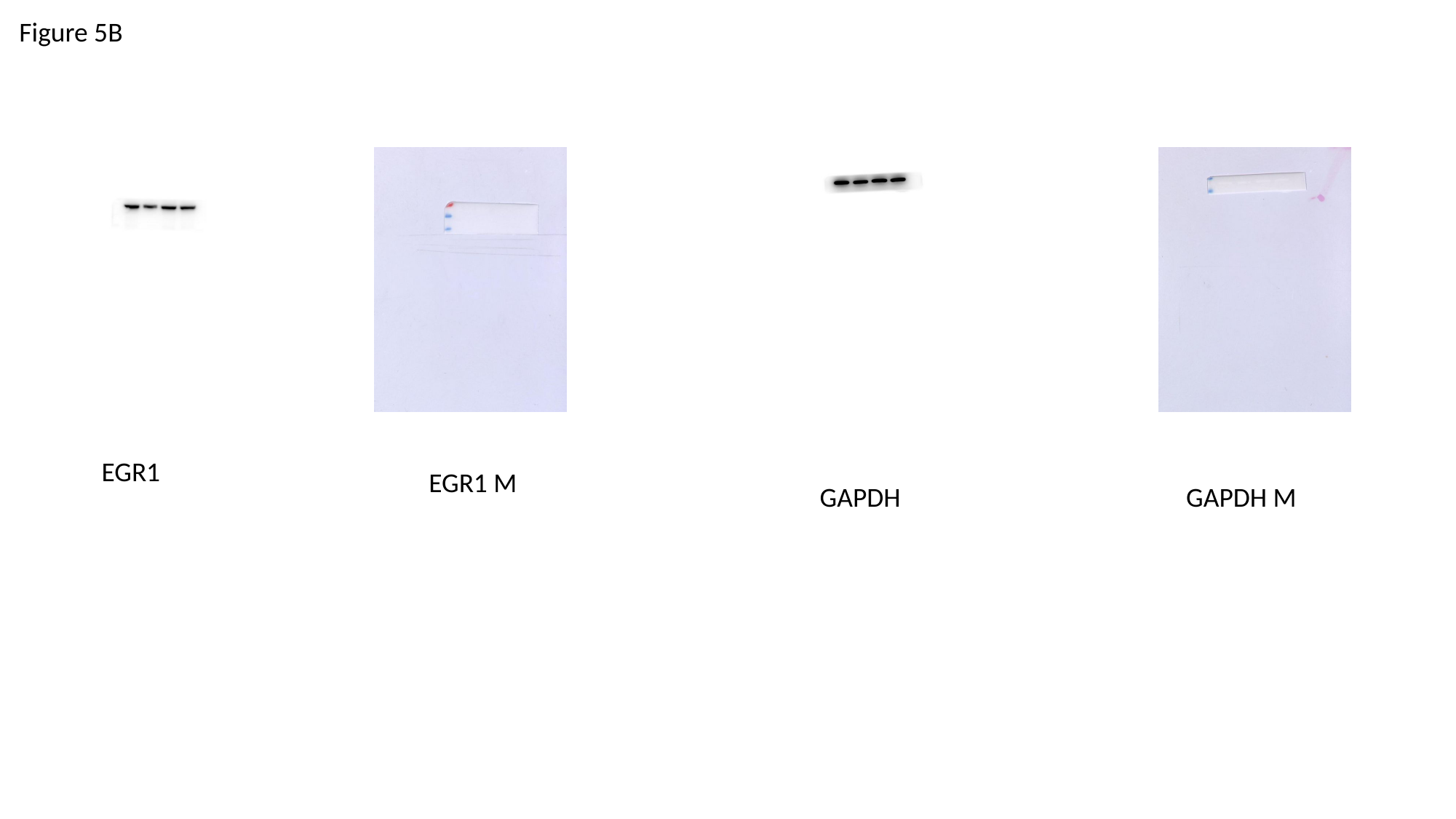

Figure 5B
EGR1
EGR1 M
GAPDH
GAPDH M

## Slide 15
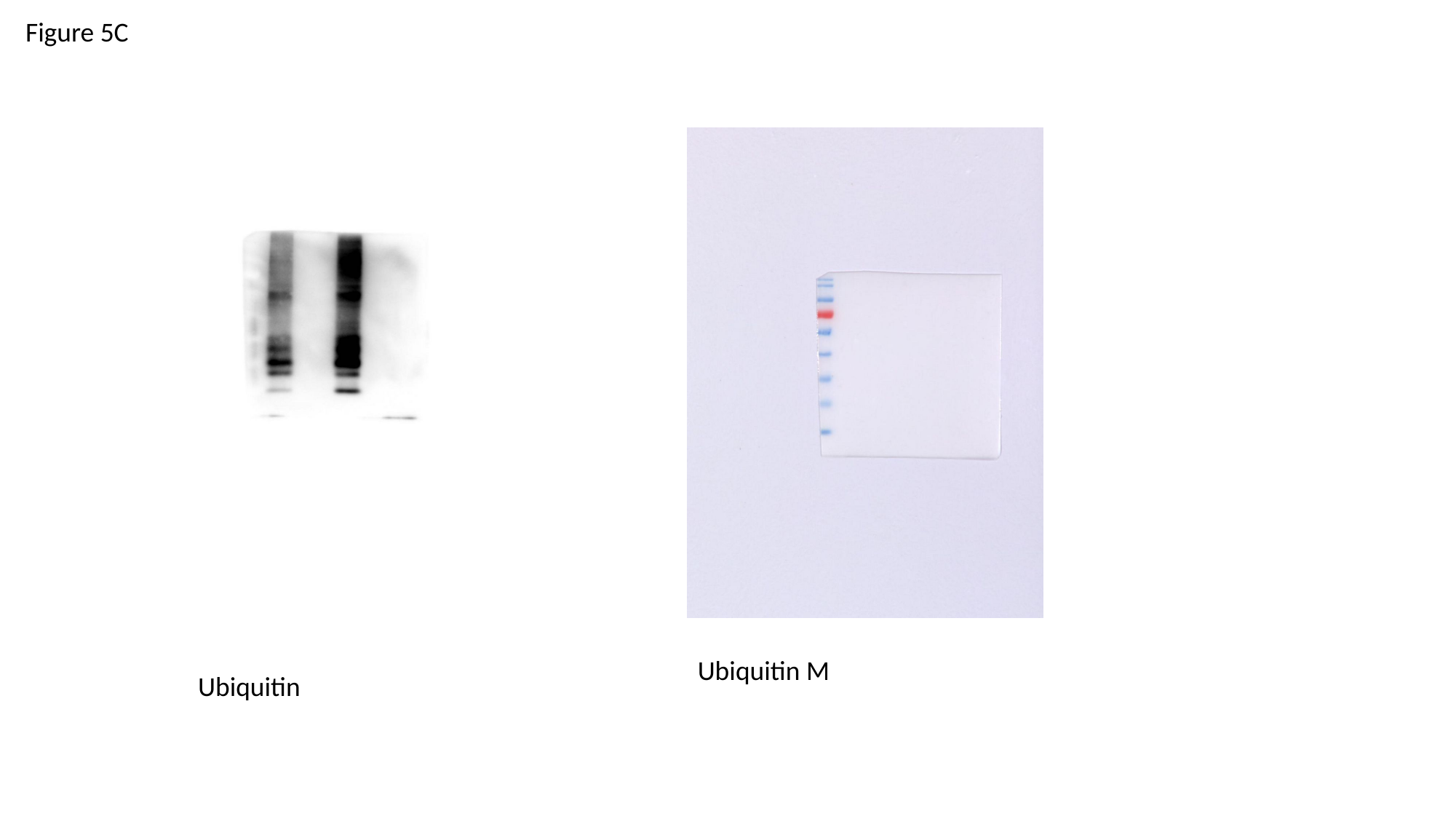

Figure 5C
Ubiquitin M
Ubiquitin

## Slide 16
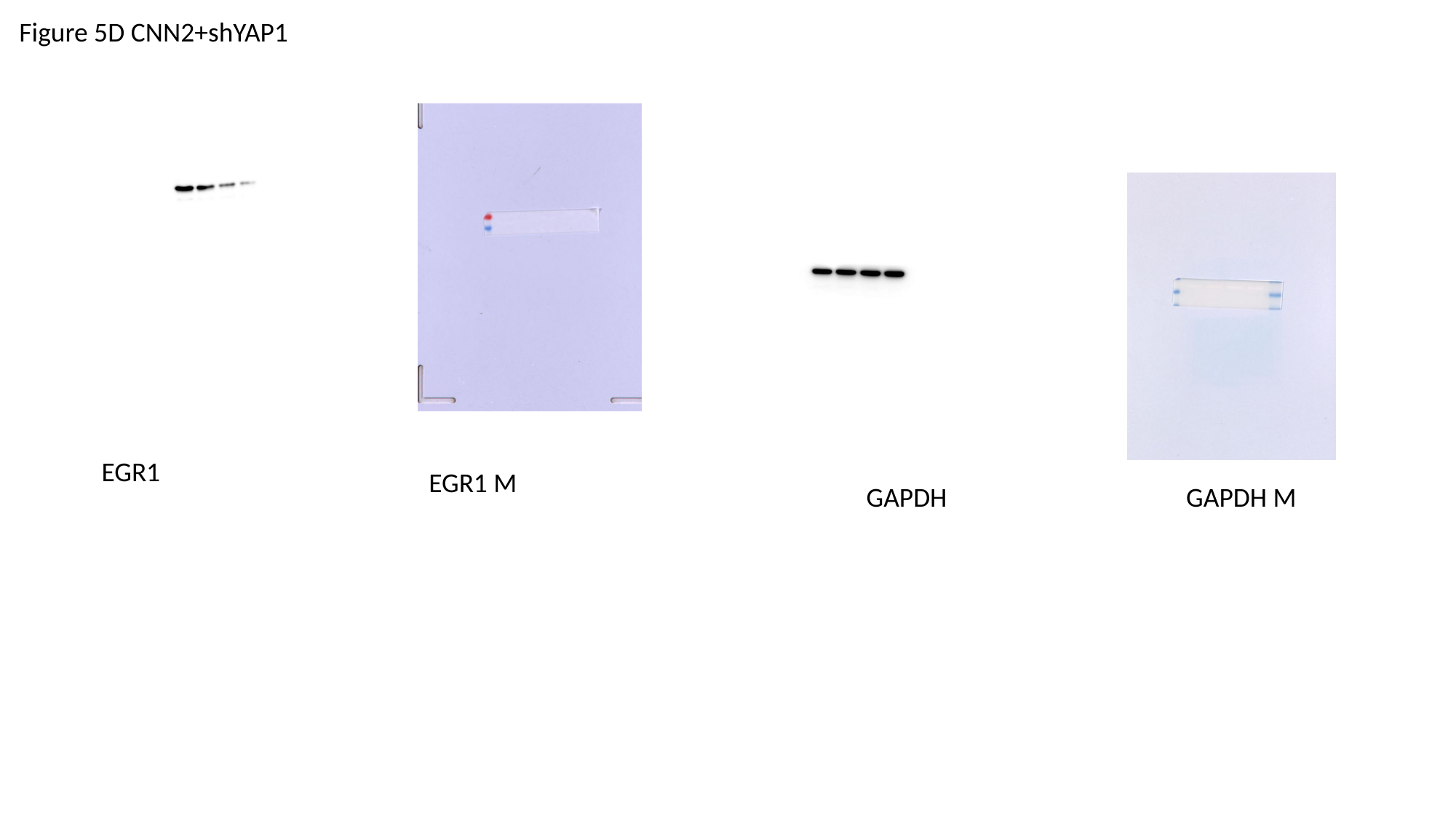

Figure 5D CNN2+shYAP1
EGR1
EGR1 M
GAPDH
GAPDH M

## Slide 17
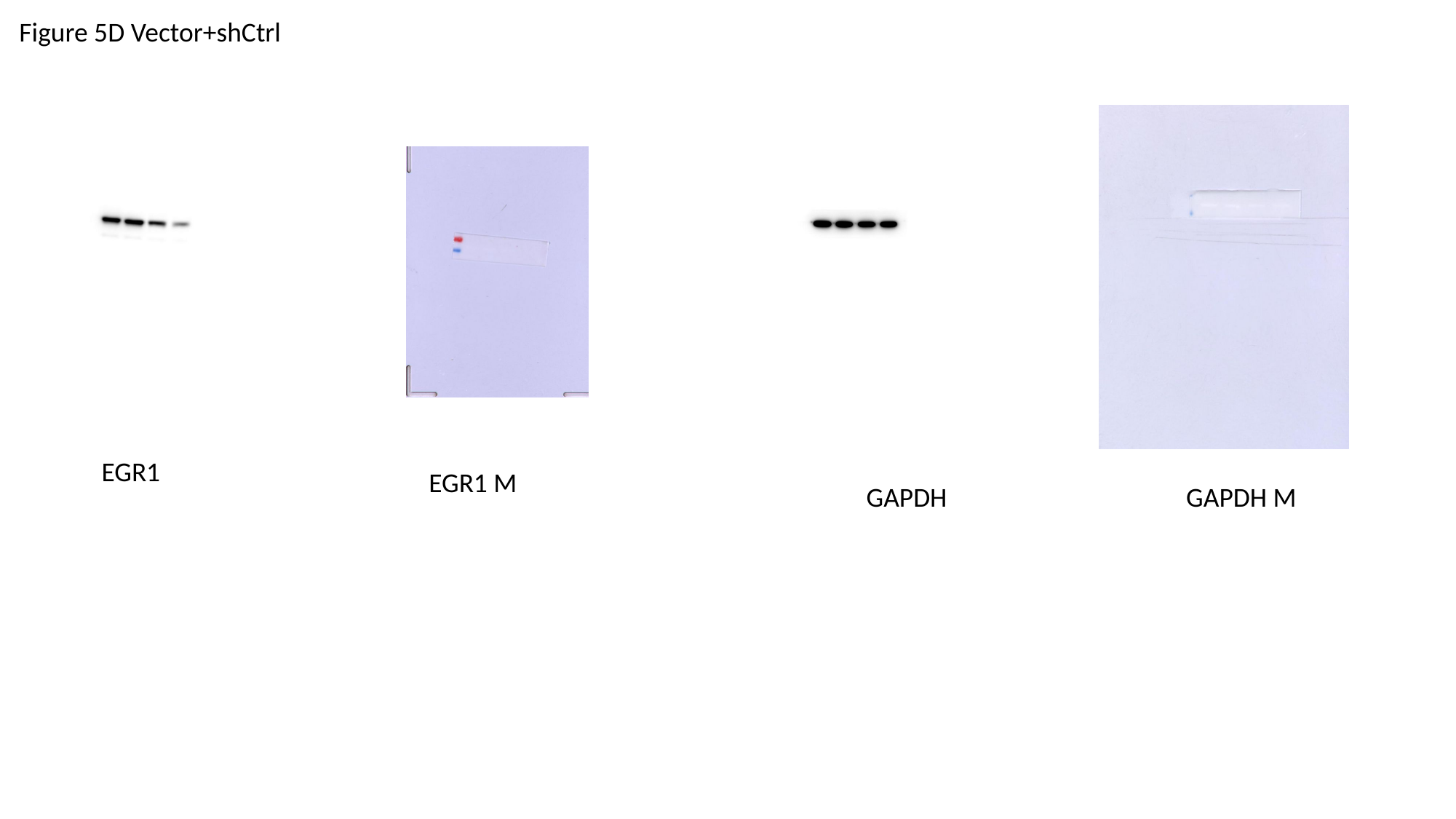

Figure 5D Vector+shCtrl
EGR1
EGR1 M
GAPDH
GAPDH M

## Slide 18
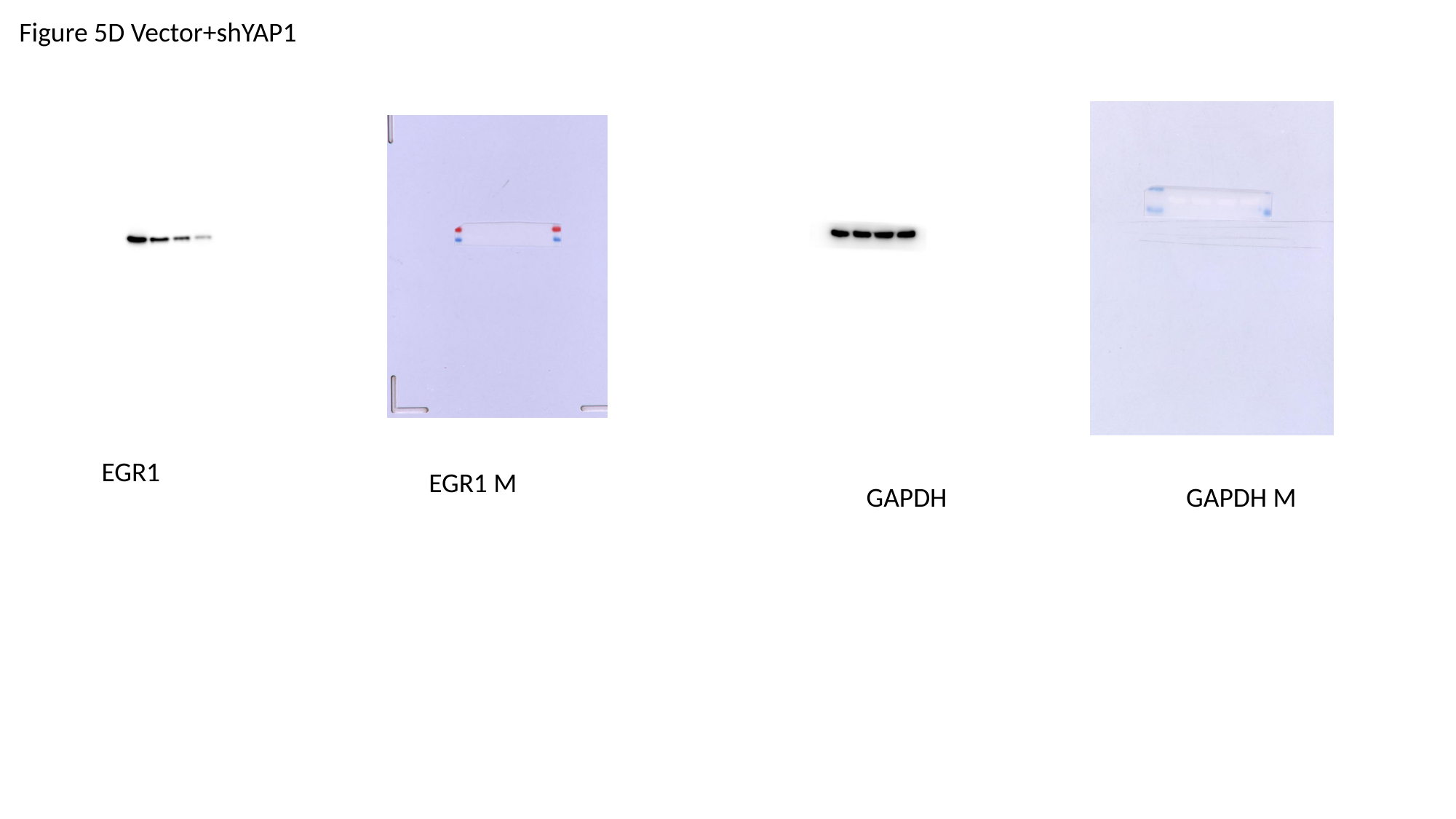

Figure 5D Vector+shYAP1
EGR1
EGR1 M
GAPDH
GAPDH M

## Slide 19
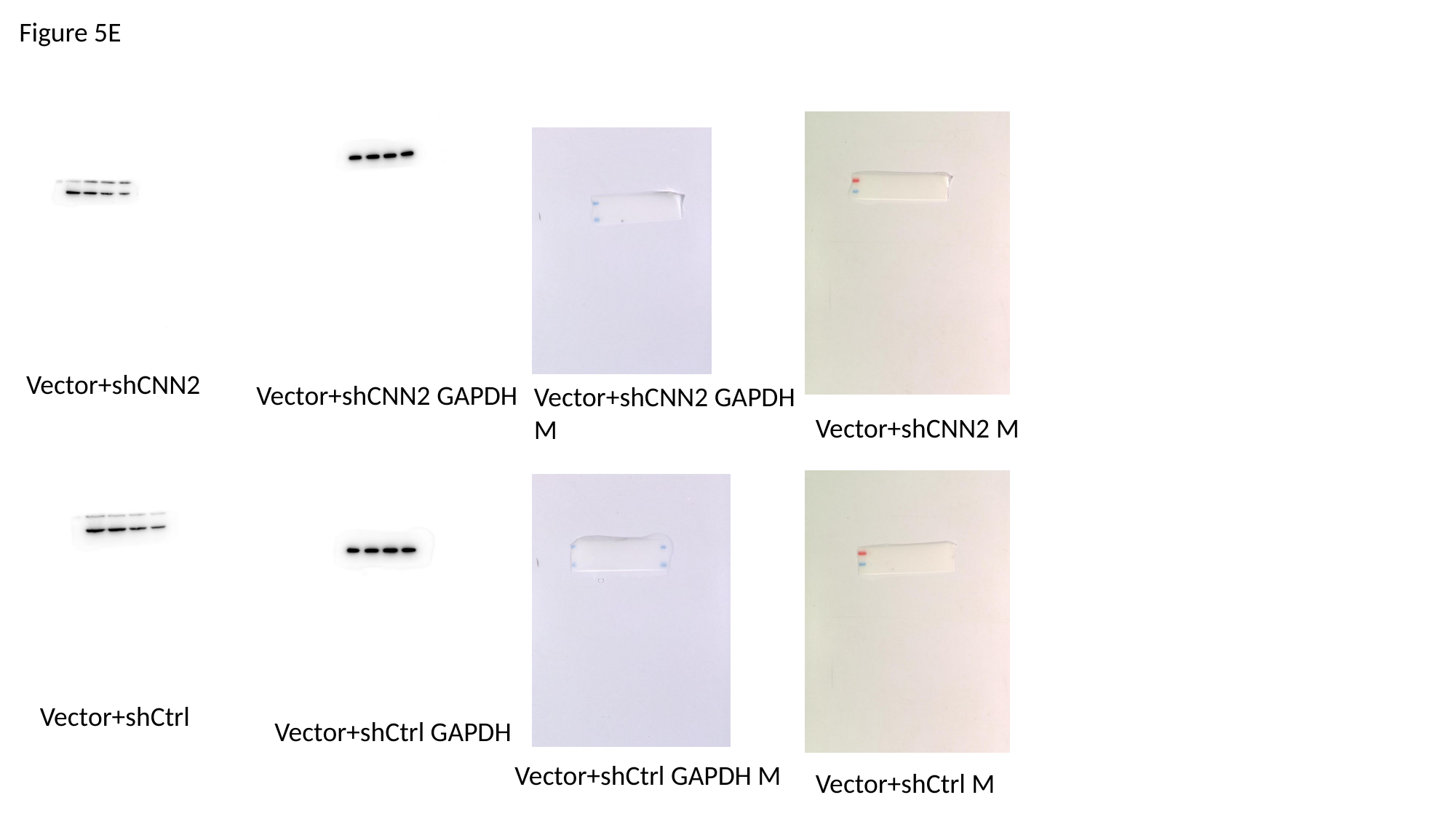

Figure 5E
Vector+shCNN2
Vector+shCNN2 GAPDH
Vector+shCNN2 GAPDH M
Vector+shCNN2 M
Vector+shCtrl
Vector+shCtrl GAPDH
Vector+shCtrl GAPDH M
Vector+shCtrl M

## Slide 20
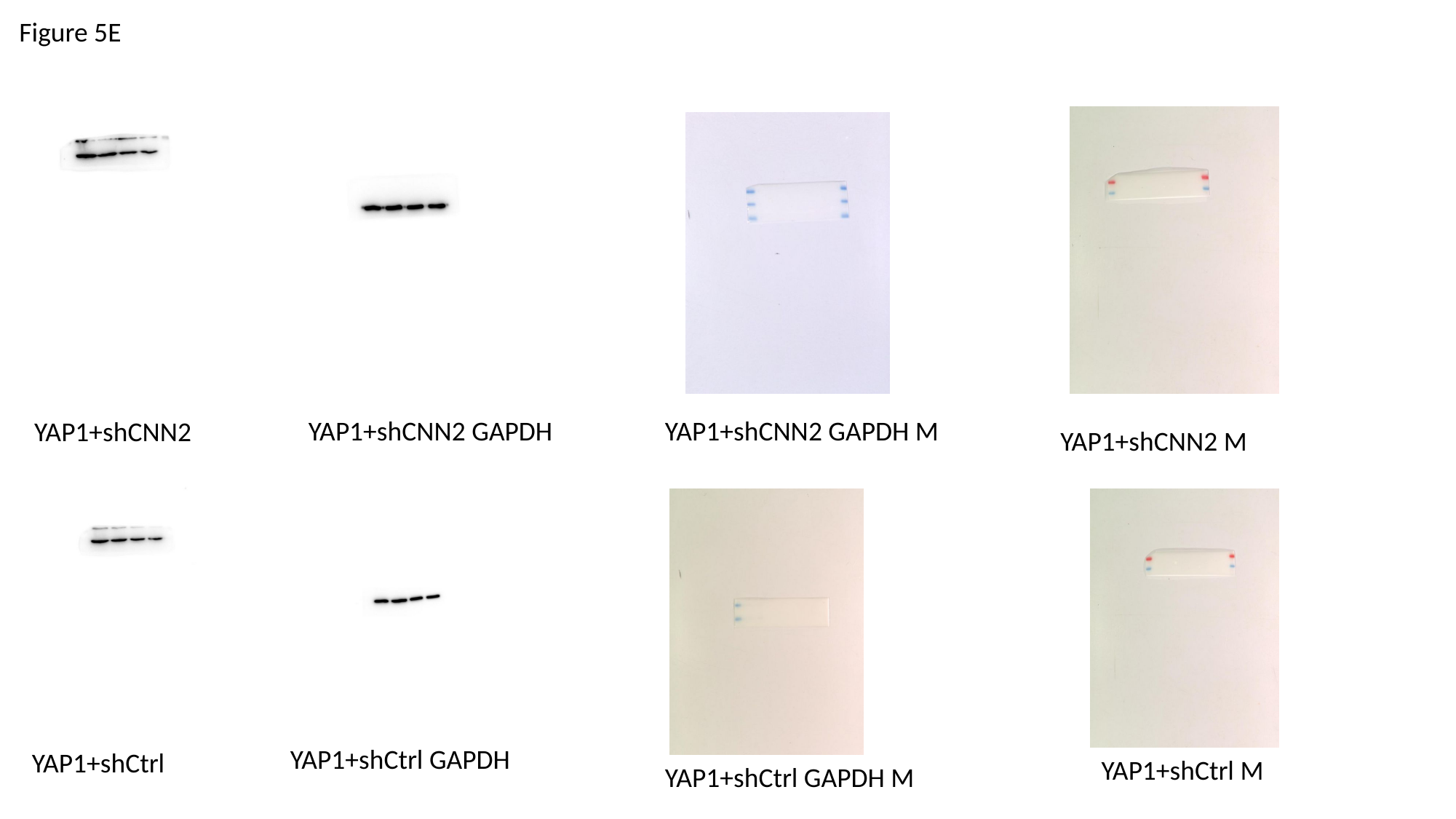

Figure 5E
YAP1+shCNN2 GAPDH
YAP1+shCNN2 GAPDH M
YAP1+shCNN2
YAP1+shCNN2 M
YAP1+shCtrl GAPDH
YAP1+shCtrl
YAP1+shCtrl M
YAP1+shCtrl GAPDH M
