# Supplementary material for: CNN2 silencing inhibits colorectal cancer development through promoting ubiquitination of EGR1
Source: Life Sci Alliance. 2023 May 15;6(7):e202201639. doi: 10.26508/lsa.202201639 (PMC10185810; doi:10.26508/lsa.202201639)
Supplement: Supplementary file 2 [file LSA-2022-01639_TableS1.docx]

Table S1 Relationship between CNN2 expression and tumor characteristics in patients with colorectal cancer analyzed by Spearman rank correlation analysis

| Tumor characteristics | index |  |
| --- | --- | --- |
| [lymphatic](D:/360%E5%AE%89%E5%85%A8%E6%B5%8F%E8%A7%88%E5%99%A8%E4%B8%8B%E8%BD%BD/Dict/8.4.0.0/resultui/html/index.html#/javascript:;) [metastasis](D:/360%E5%AE%89%E5%85%A8%E6%B5%8F%E8%A7%88%E5%99%A8%E4%B8%8B%E8%BD%BD/Dict/8.4.0.0/resultui/html/index.html#/javascript:;) (N) | Spearman correlation | 0.328 |
|  | Significance (two tailed) | 0.001 |
|  | n | 100 |
| Stage | Spearman correlation | 0.294 |
|  | Significance (two tailed) | 0.003 |
|  | n | 100 |
