# Supplementary material for: CNN2 silencing inhibits colorectal cancer development through promoting ubiquitination of EGR1
Source: Life Sci Alliance. 2023 May 15;6(7):e202201639. doi: 10.26508/lsa.202201639 (PMC10185810; doi:10.26508/lsa.202201639)
Supplement: Supplementary file 3 [file LSA-2022-01639_SdataF2.1.pptx]

## Slide 1
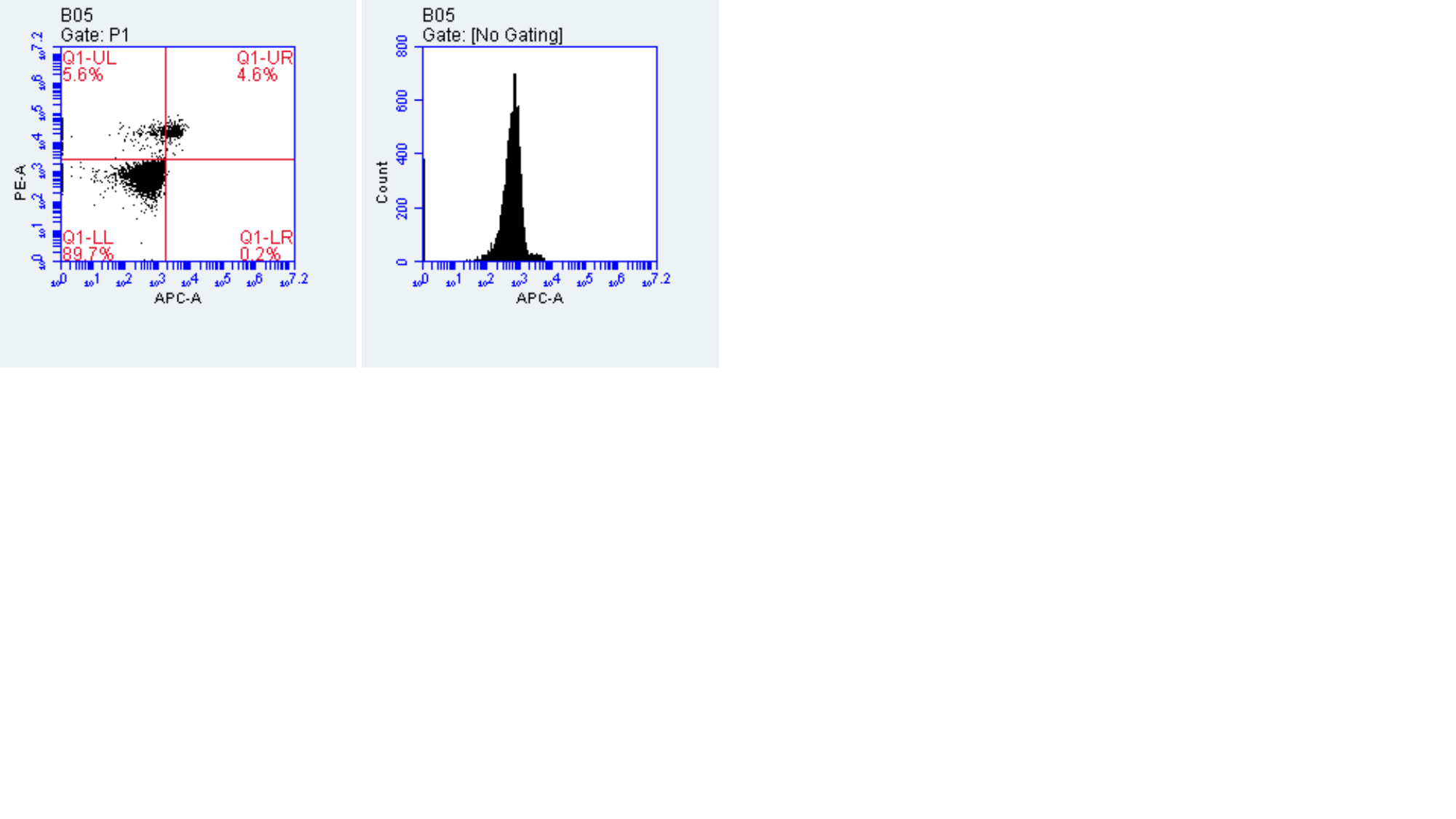

## Slide 2
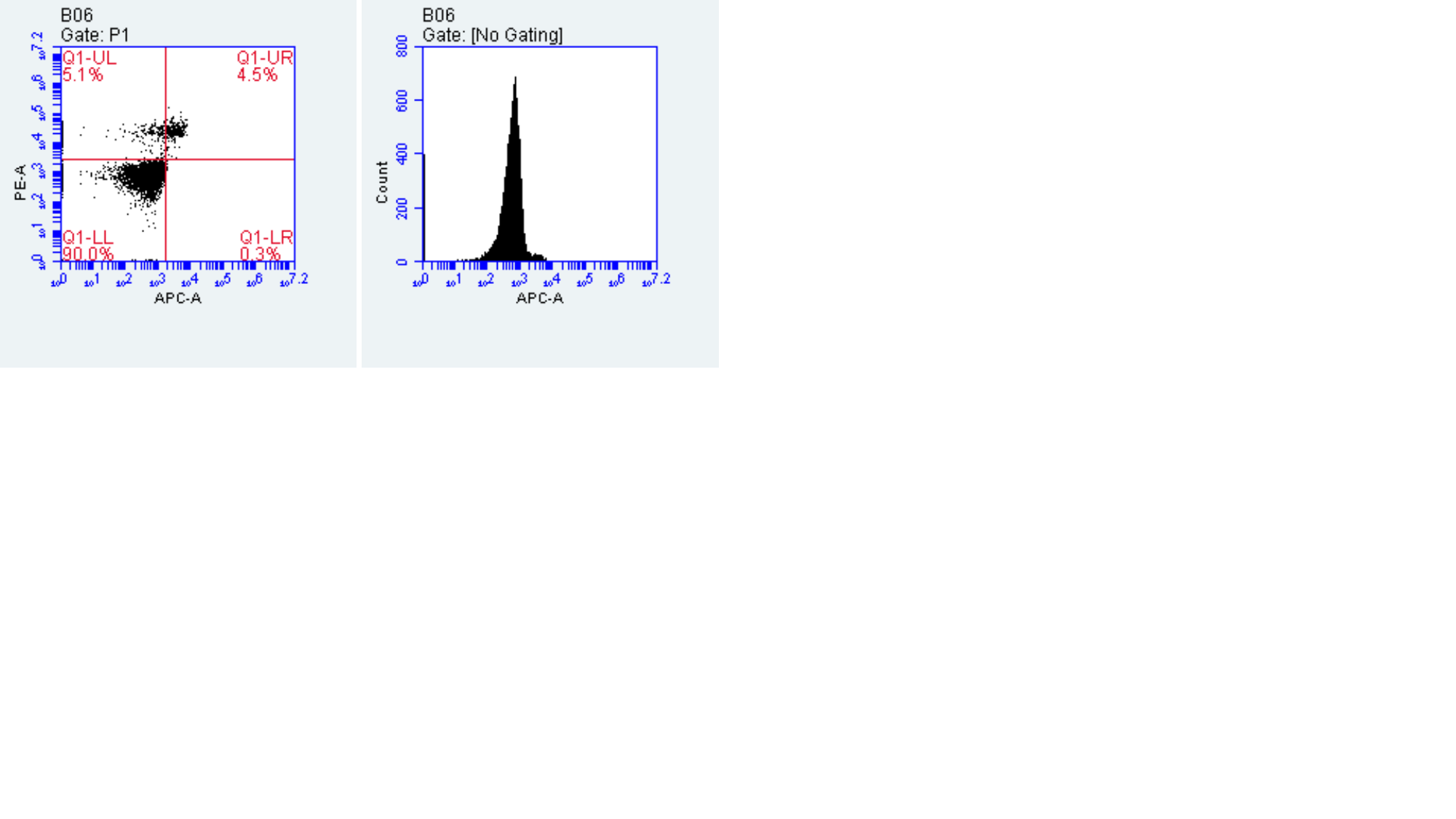

## Slide 3
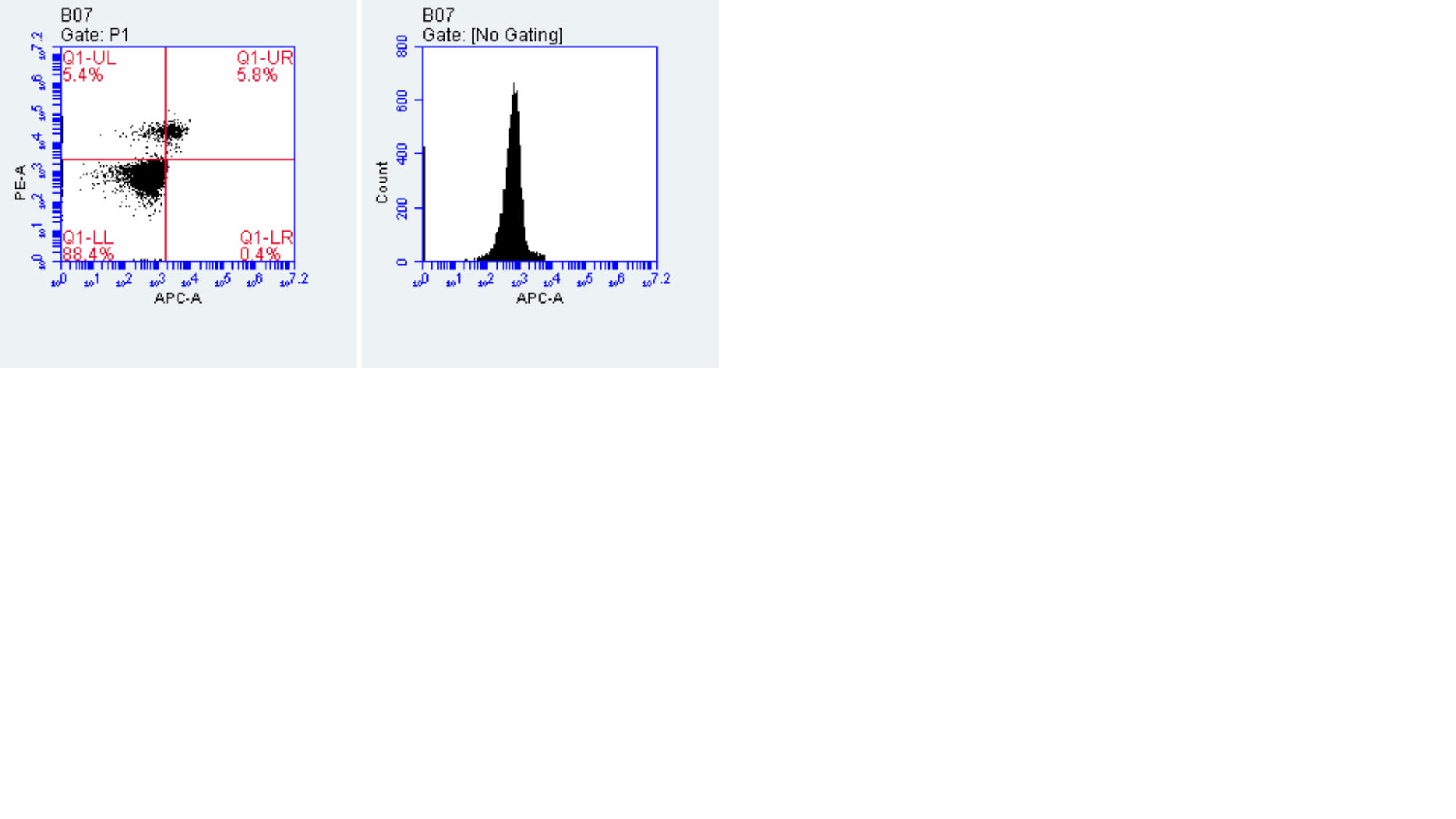

## Slide 4
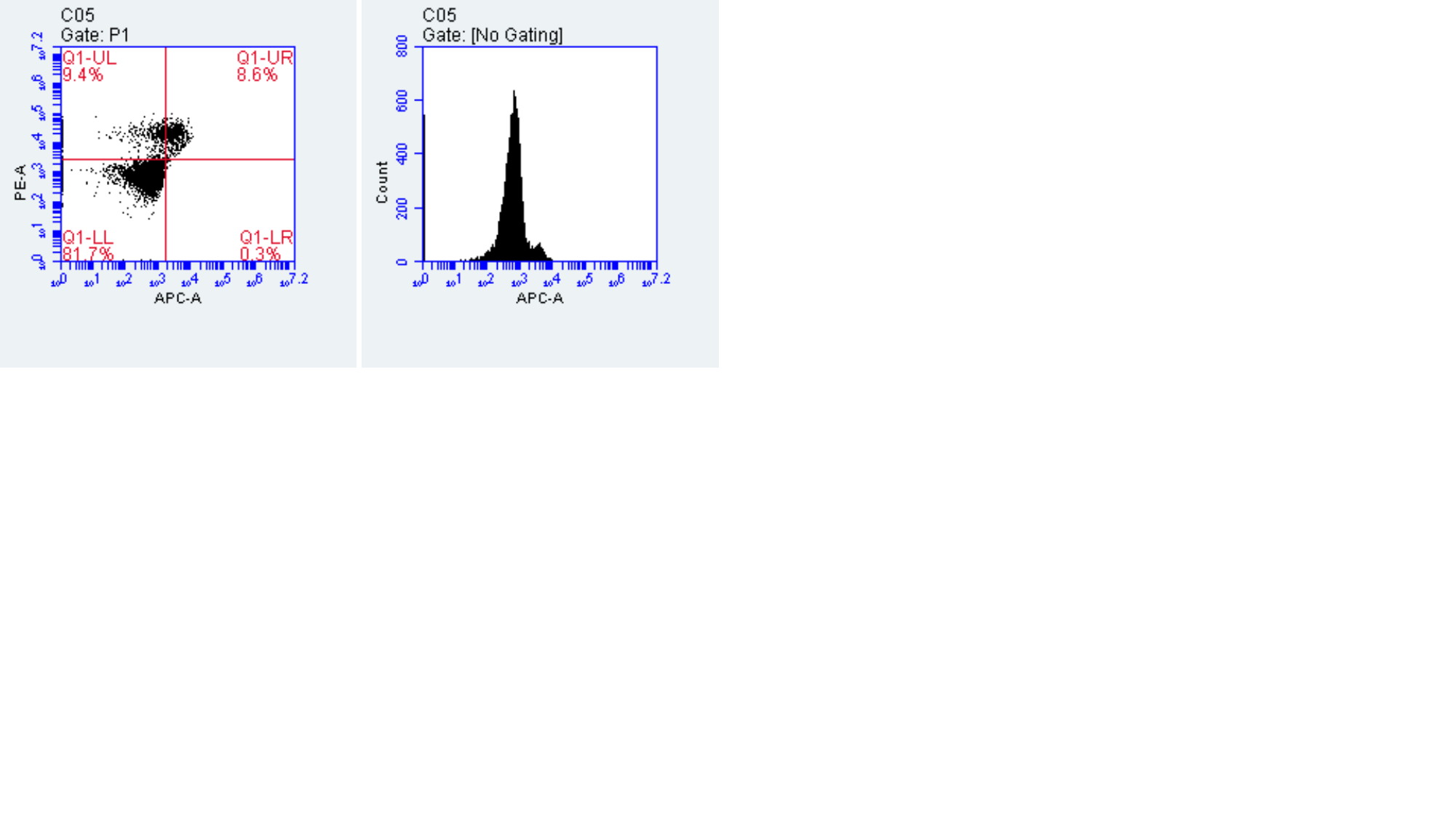

## Slide 5
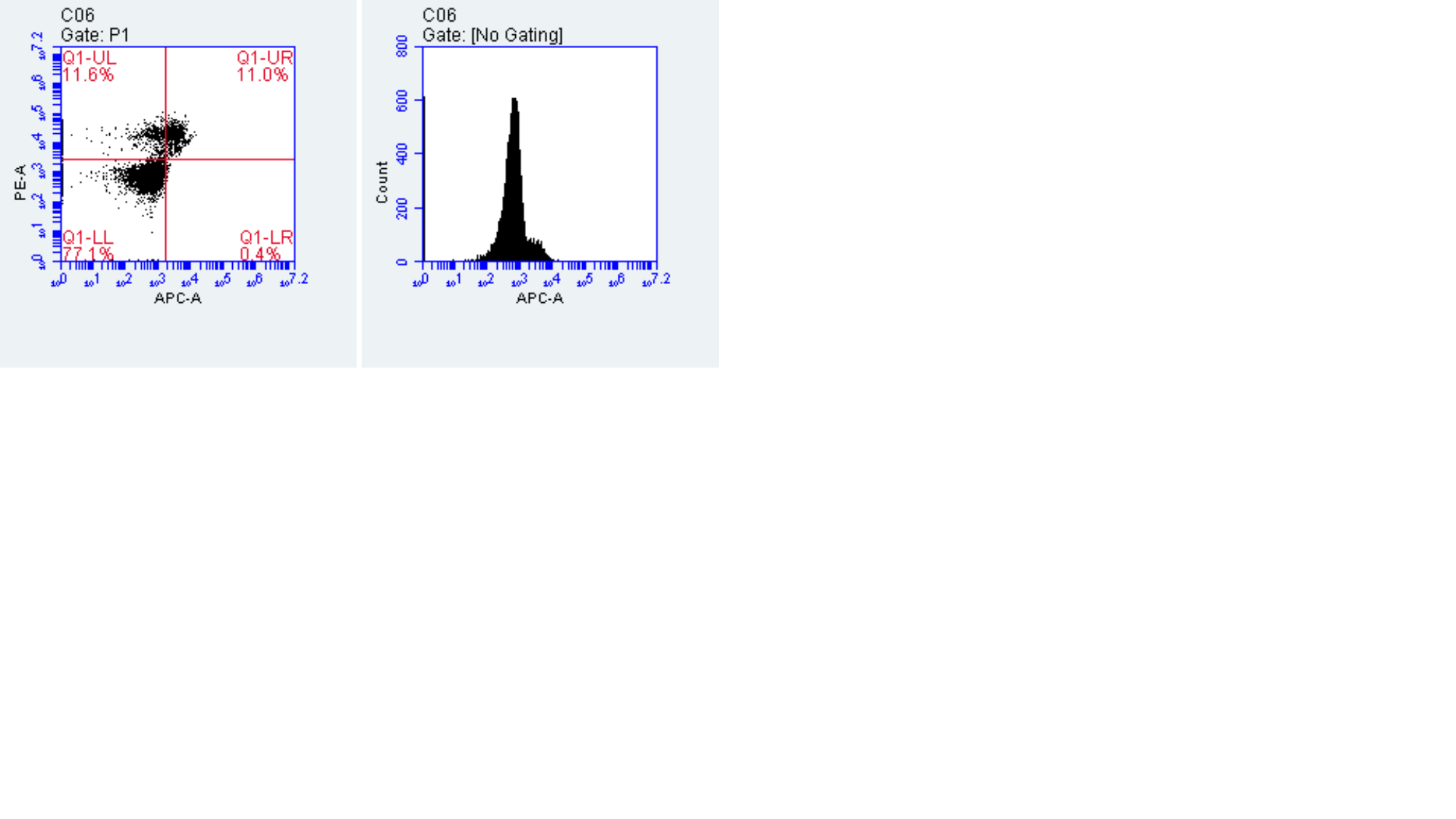

## Slide 6
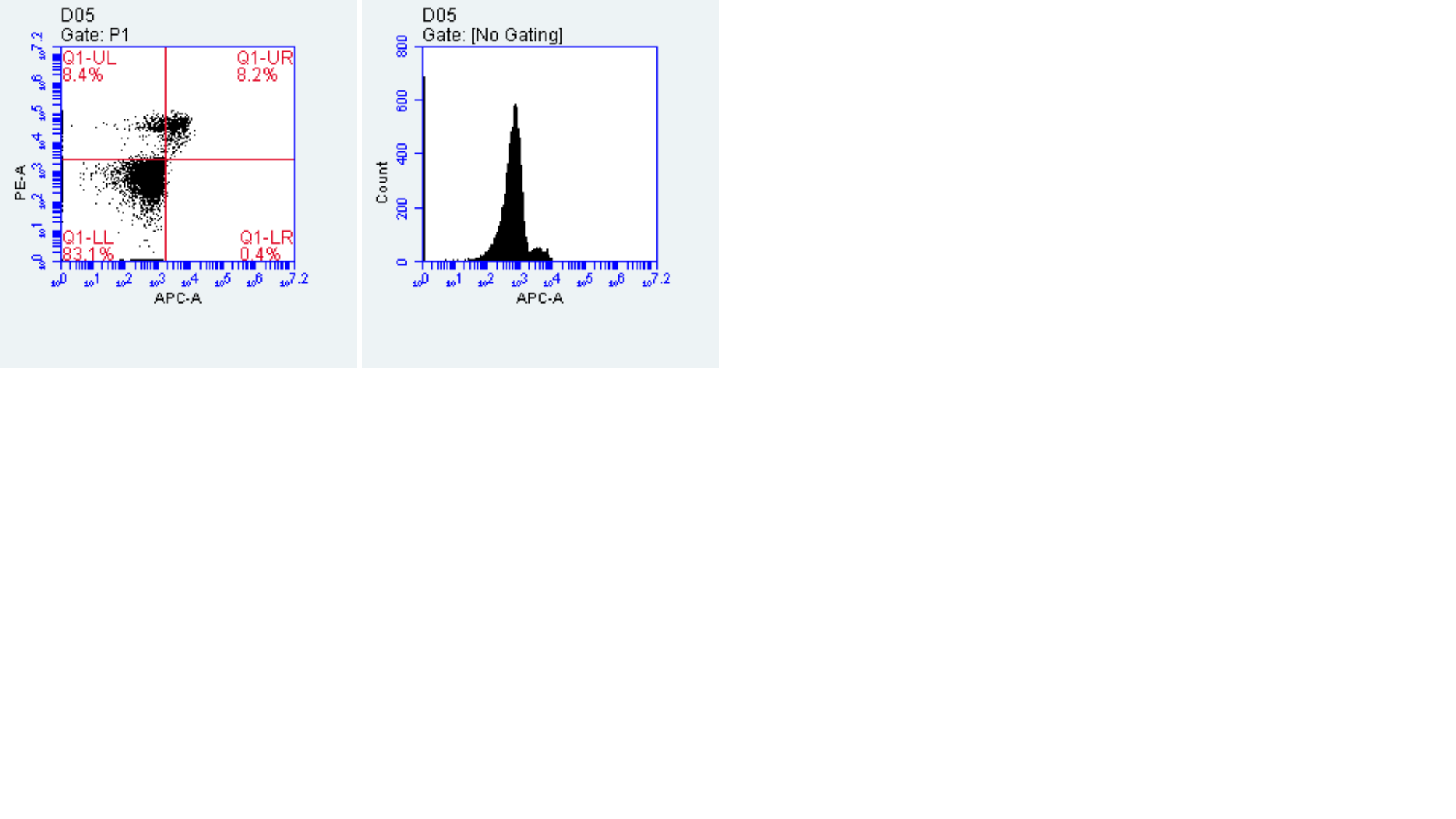

## Slide 7
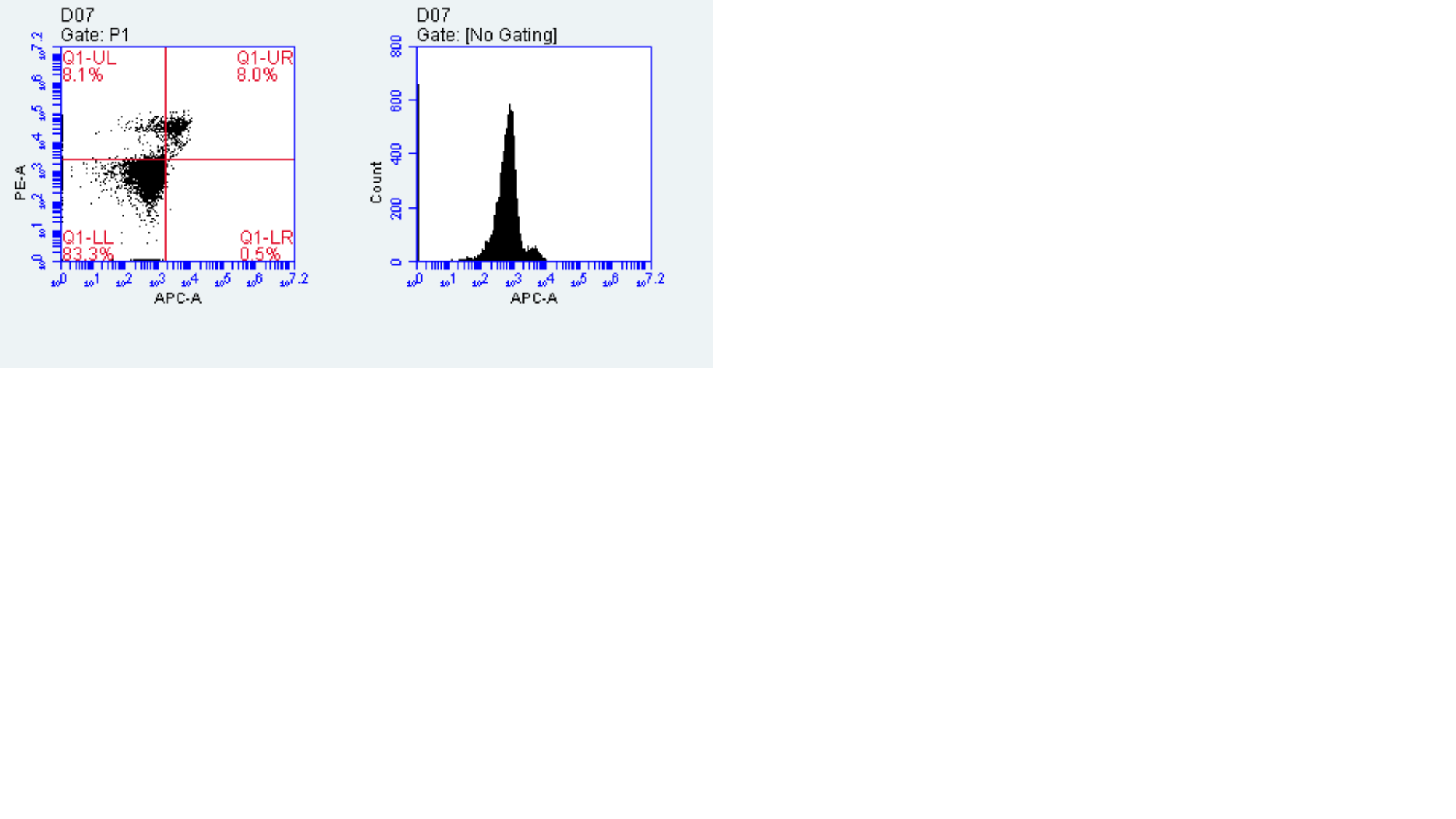

## Slide 8
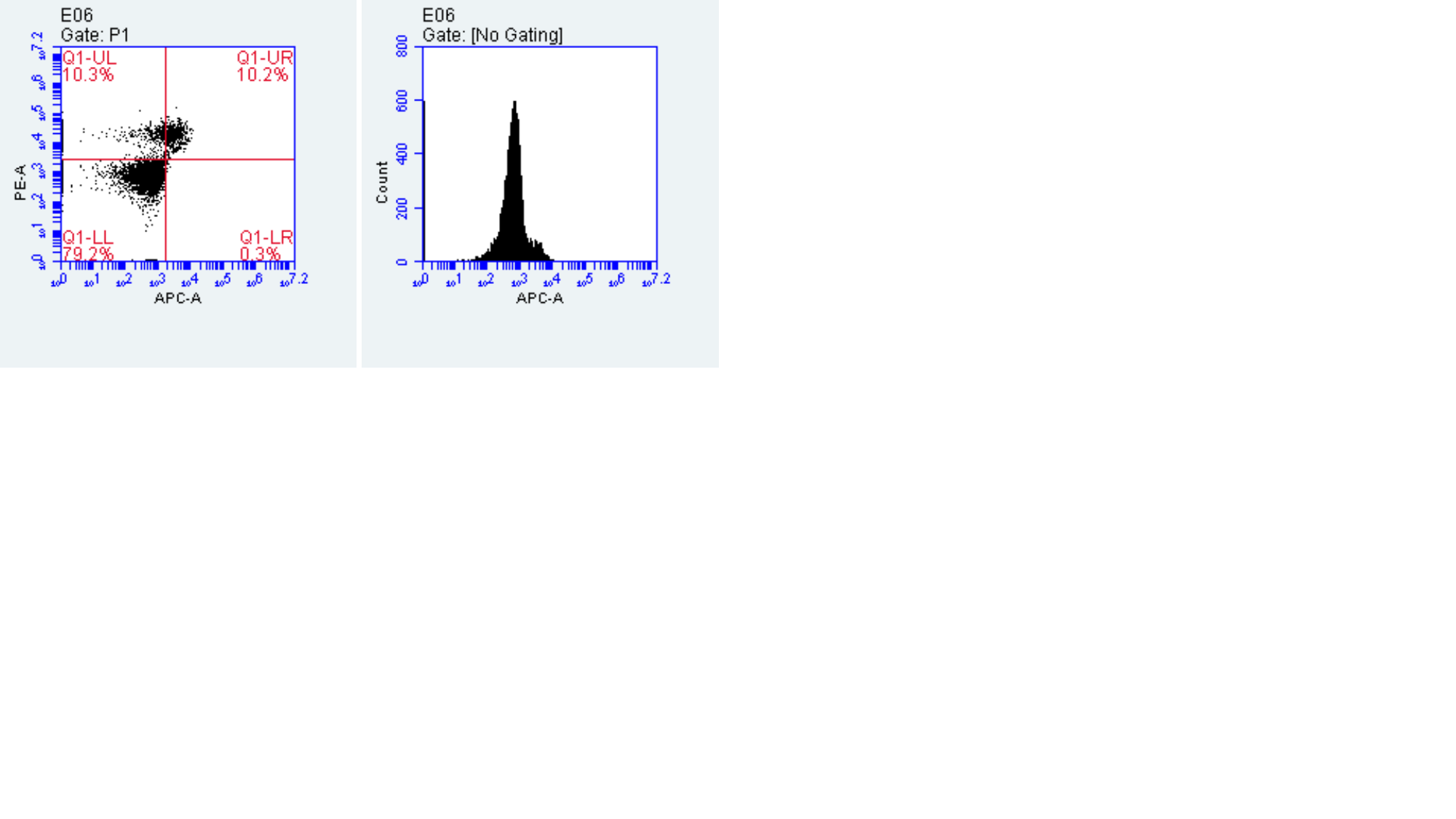

## Slide 9
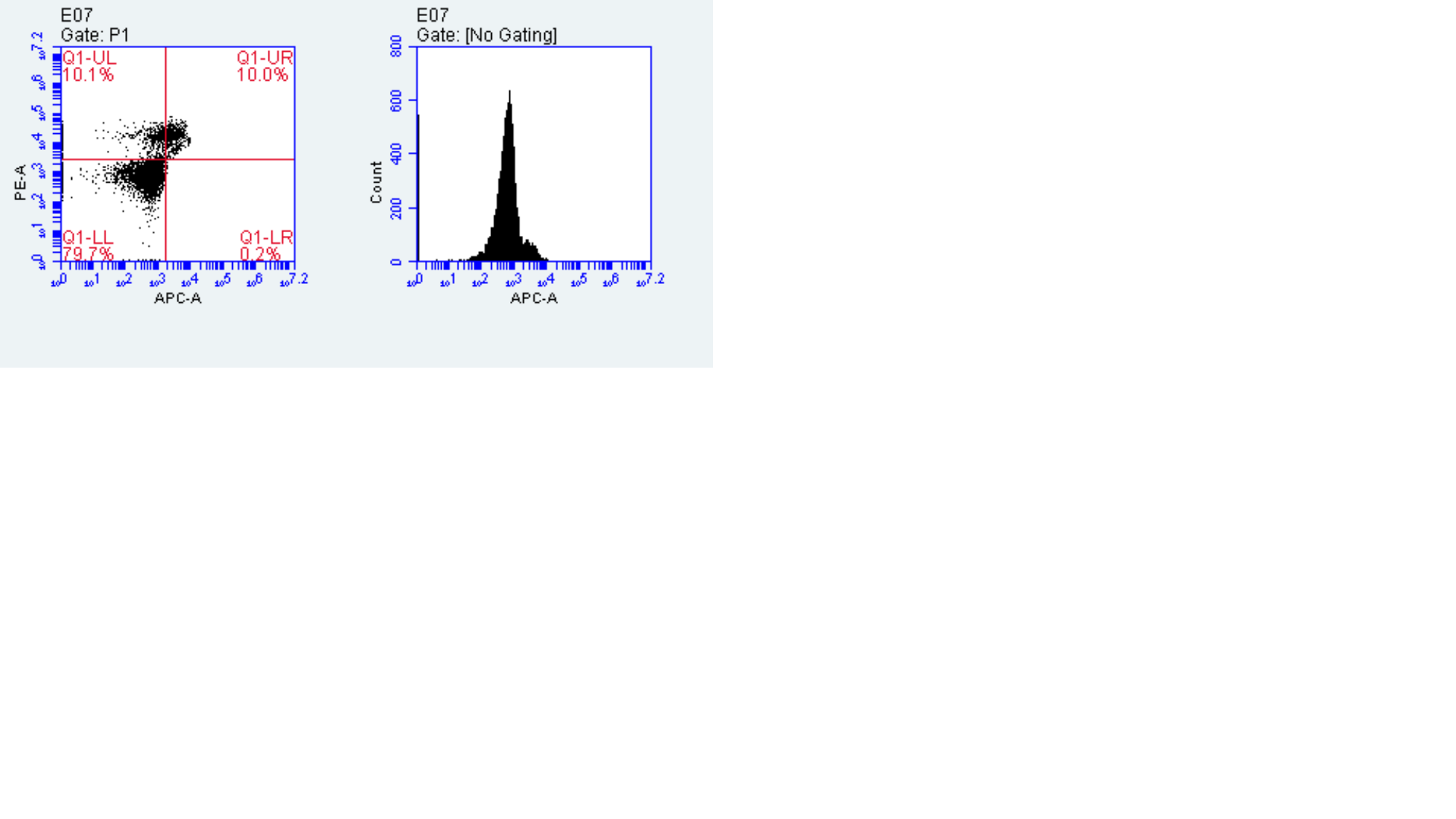

## Slide 10
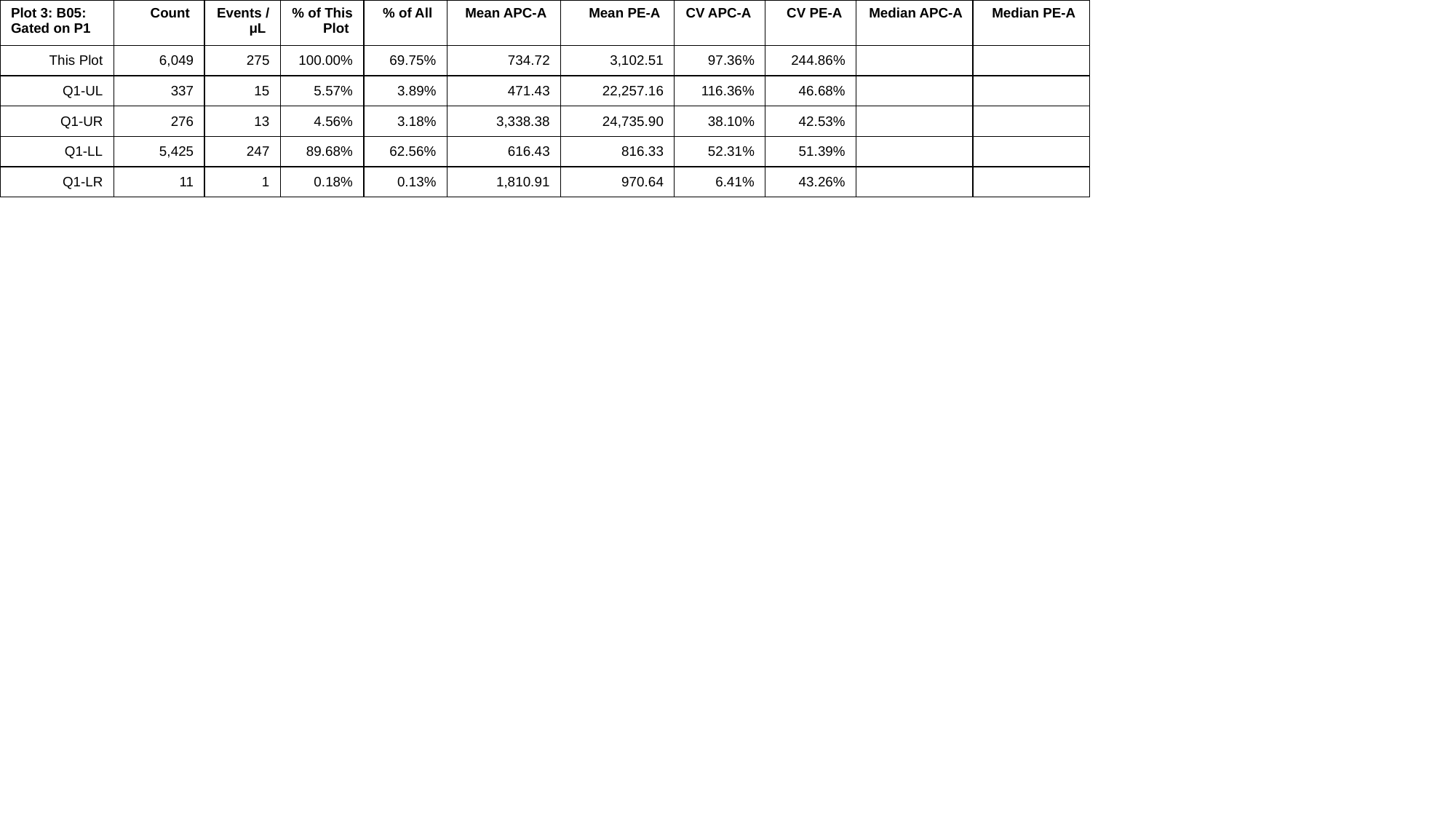

| Plot 3: B05: Gated on P1 | Count | Events / μL | % of This Plot | % of All | Mean APC-A | Mean PE-A | CV APC-A | CV PE-A | Median APC-A | Median PE-A |
| --- | --- | --- | --- | --- | --- | --- | --- | --- | --- | --- |
| This Plot | 6,049 | 275 | 100.00% | 69.75% | 734.72 | 3,102.51 | 97.36% | 244.86% | | |
| Q1-UL | 337 | 15 | 5.57% | 3.89% | 471.43 | 22,257.16 | 116.36% | 46.68% | | |
| Q1-UR | 276 | 13 | 4.56% | 3.18% | 3,338.38 | 24,735.90 | 38.10% | 42.53% | | |
| Q1-LL | 5,425 | 247 | 89.68% | 62.56% | 616.43 | 816.33 | 52.31% | 51.39% | | |
| Q1-LR | 11 | 1 | 0.18% | 0.13% | 1,810.91 | 970.64 | 6.41% | 43.26% | | |

## Slide 11
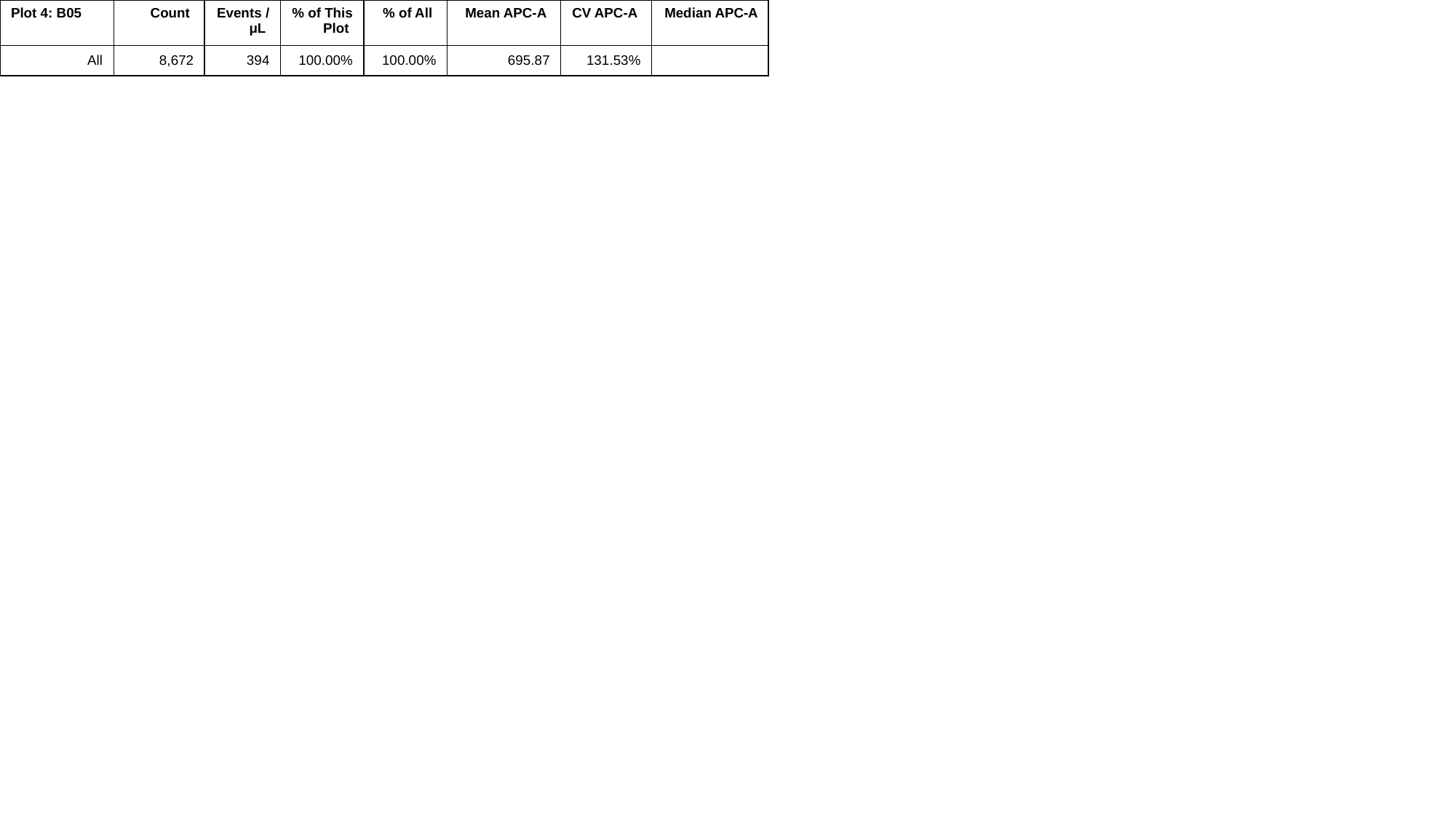

| Plot 4: B05 | Count | Events / μL | % of This Plot | % of All | Mean APC-A | CV APC-A | Median APC-A |
| --- | --- | --- | --- | --- | --- | --- | --- |
| All | 8,672 | 394 | 100.00% | 100.00% | 695.87 | 131.53% | |

## Slide 12
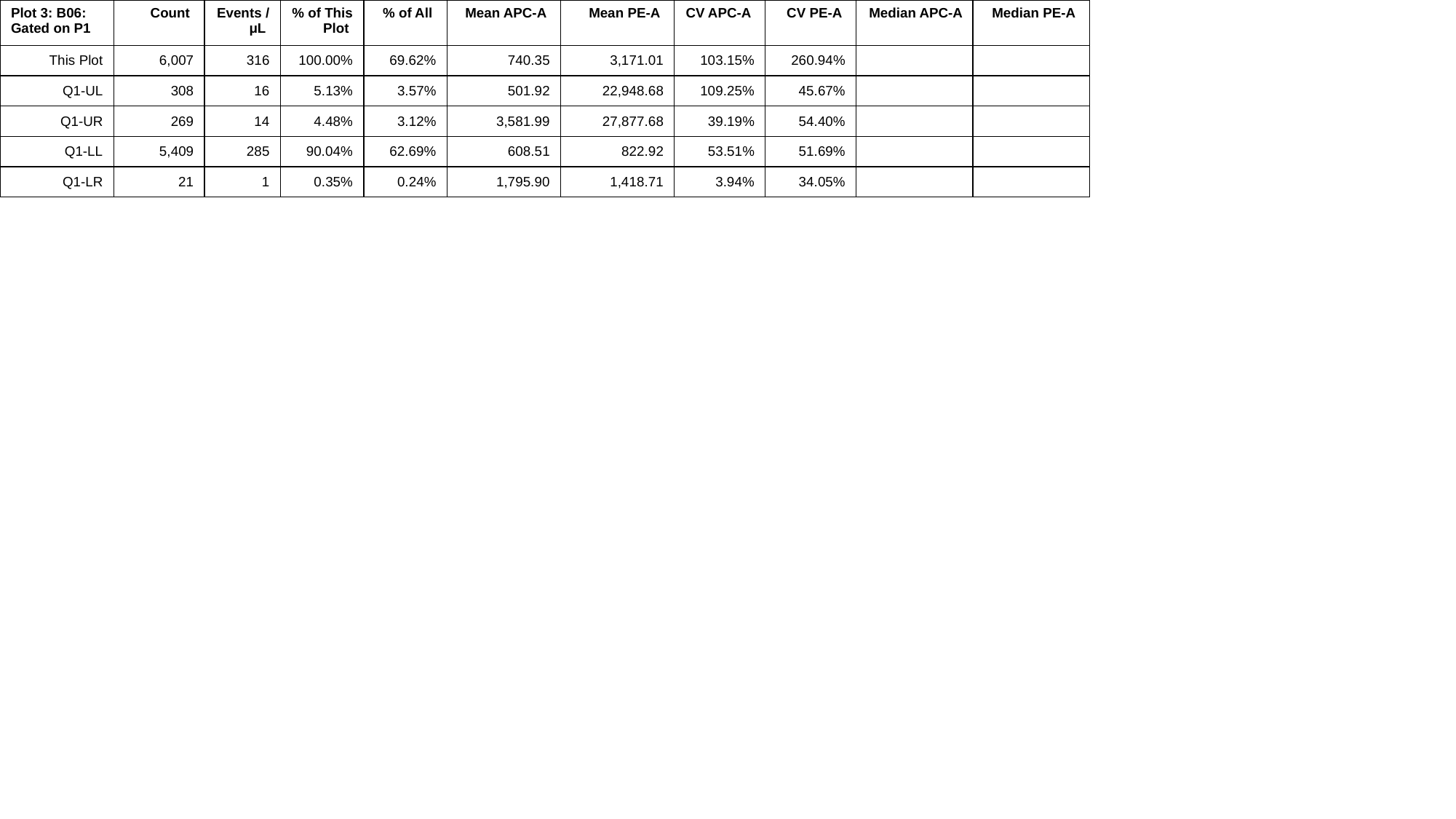

| Plot 3: B06: Gated on P1 | Count | Events / μL | % of This Plot | % of All | Mean APC-A | Mean PE-A | CV APC-A | CV PE-A | Median APC-A | Median PE-A |
| --- | --- | --- | --- | --- | --- | --- | --- | --- | --- | --- |
| This Plot | 6,007 | 316 | 100.00% | 69.62% | 740.35 | 3,171.01 | 103.15% | 260.94% | | |
| Q1-UL | 308 | 16 | 5.13% | 3.57% | 501.92 | 22,948.68 | 109.25% | 45.67% | | |
| Q1-UR | 269 | 14 | 4.48% | 3.12% | 3,581.99 | 27,877.68 | 39.19% | 54.40% | | |
| Q1-LL | 5,409 | 285 | 90.04% | 62.69% | 608.51 | 822.92 | 53.51% | 51.69% | | |
| Q1-LR | 21 | 1 | 0.35% | 0.24% | 1,795.90 | 1,418.71 | 3.94% | 34.05% | | |

## Slide 13
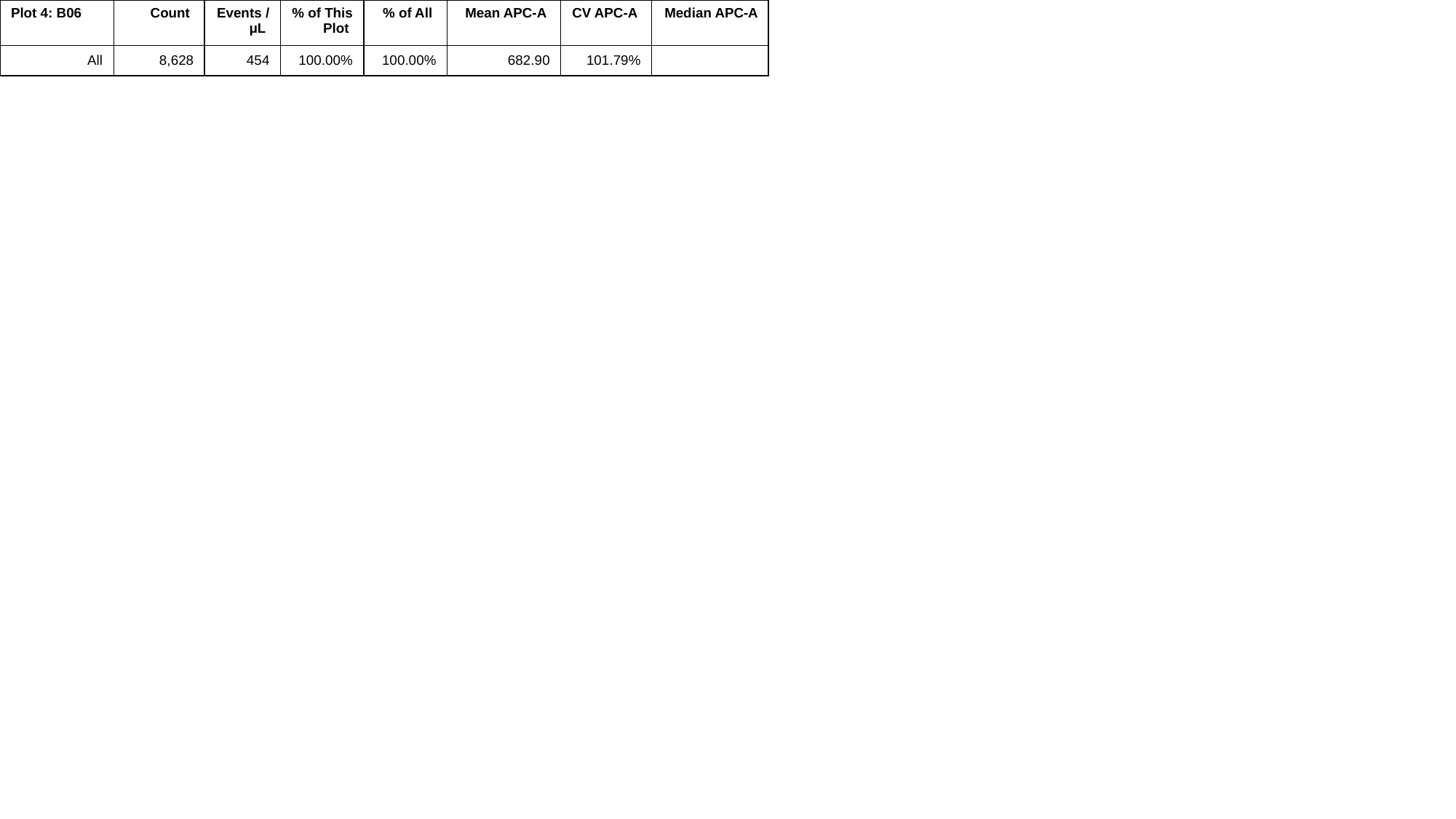

| Plot 4: B06 | Count | Events / μL | % of This Plot | % of All | Mean APC-A | CV APC-A | Median APC-A |
| --- | --- | --- | --- | --- | --- | --- | --- |
| All | 8,628 | 454 | 100.00% | 100.00% | 682.90 | 101.79% | |

## Slide 14
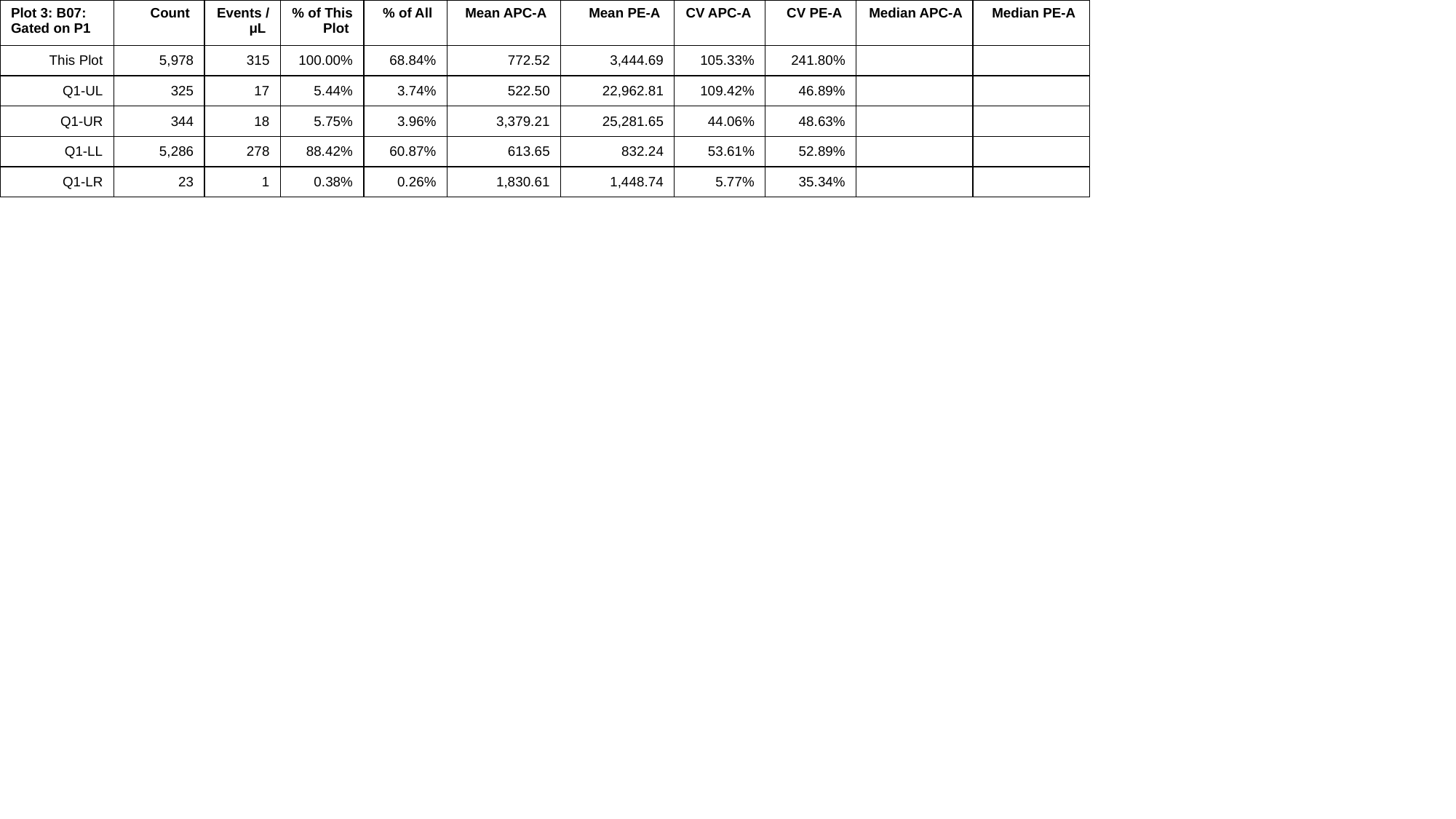

| Plot 3: B07: Gated on P1 | Count | Events / μL | % of This Plot | % of All | Mean APC-A | Mean PE-A | CV APC-A | CV PE-A | Median APC-A | Median PE-A |
| --- | --- | --- | --- | --- | --- | --- | --- | --- | --- | --- |
| This Plot | 5,978 | 315 | 100.00% | 68.84% | 772.52 | 3,444.69 | 105.33% | 241.80% | | |
| Q1-UL | 325 | 17 | 5.44% | 3.74% | 522.50 | 22,962.81 | 109.42% | 46.89% | | |
| Q1-UR | 344 | 18 | 5.75% | 3.96% | 3,379.21 | 25,281.65 | 44.06% | 48.63% | | |
| Q1-LL | 5,286 | 278 | 88.42% | 60.87% | 613.65 | 832.24 | 53.61% | 52.89% | | |
| Q1-LR | 23 | 1 | 0.38% | 0.26% | 1,830.61 | 1,448.74 | 5.77% | 35.34% | | |

## Slide 15
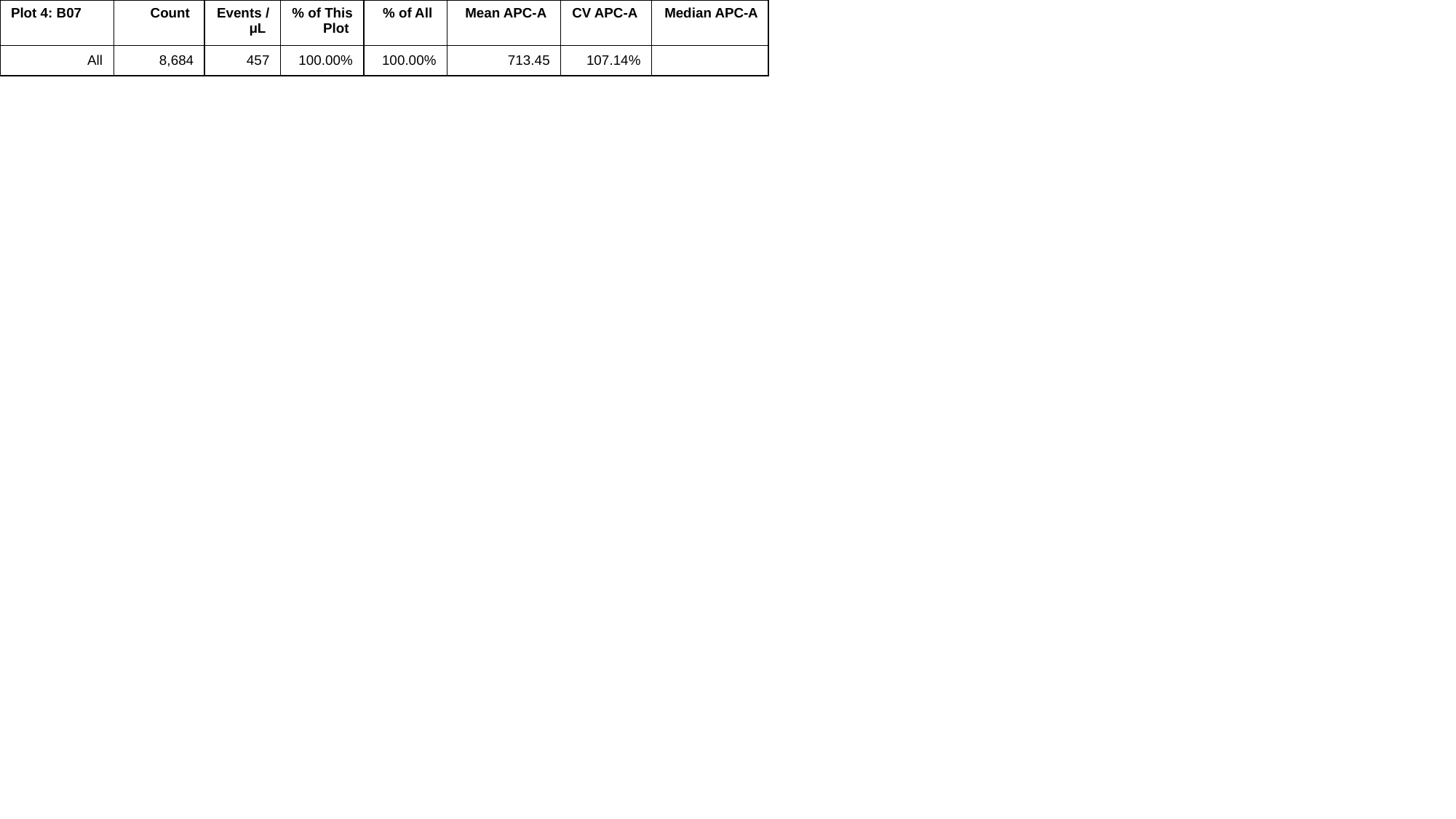

| Plot 4: B07 | Count | Events / μL | % of This Plot | % of All | Mean APC-A | CV APC-A | Median APC-A |
| --- | --- | --- | --- | --- | --- | --- | --- |
| All | 8,684 | 457 | 100.00% | 100.00% | 713.45 | 107.14% | |

## Slide 16
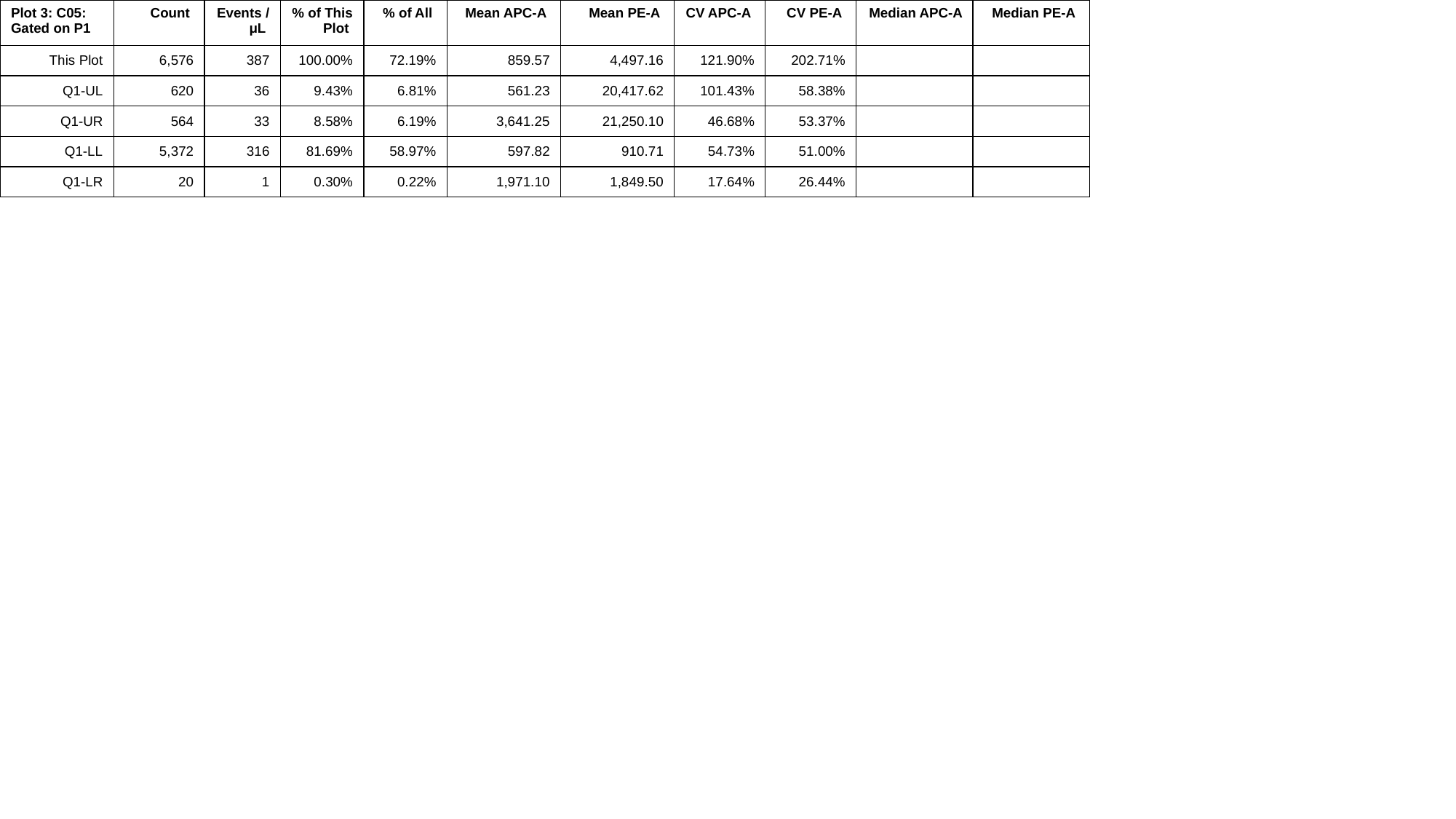

| Plot 3: C05: Gated on P1 | Count | Events / μL | % of This Plot | % of All | Mean APC-A | Mean PE-A | CV APC-A | CV PE-A | Median APC-A | Median PE-A |
| --- | --- | --- | --- | --- | --- | --- | --- | --- | --- | --- |
| This Plot | 6,576 | 387 | 100.00% | 72.19% | 859.57 | 4,497.16 | 121.90% | 202.71% | | |
| Q1-UL | 620 | 36 | 9.43% | 6.81% | 561.23 | 20,417.62 | 101.43% | 58.38% | | |
| Q1-UR | 564 | 33 | 8.58% | 6.19% | 3,641.25 | 21,250.10 | 46.68% | 53.37% | | |
| Q1-LL | 5,372 | 316 | 81.69% | 58.97% | 597.82 | 910.71 | 54.73% | 51.00% | | |
| Q1-LR | 20 | 1 | 0.30% | 0.22% | 1,971.10 | 1,849.50 | 17.64% | 26.44% | | |

## Slide 17
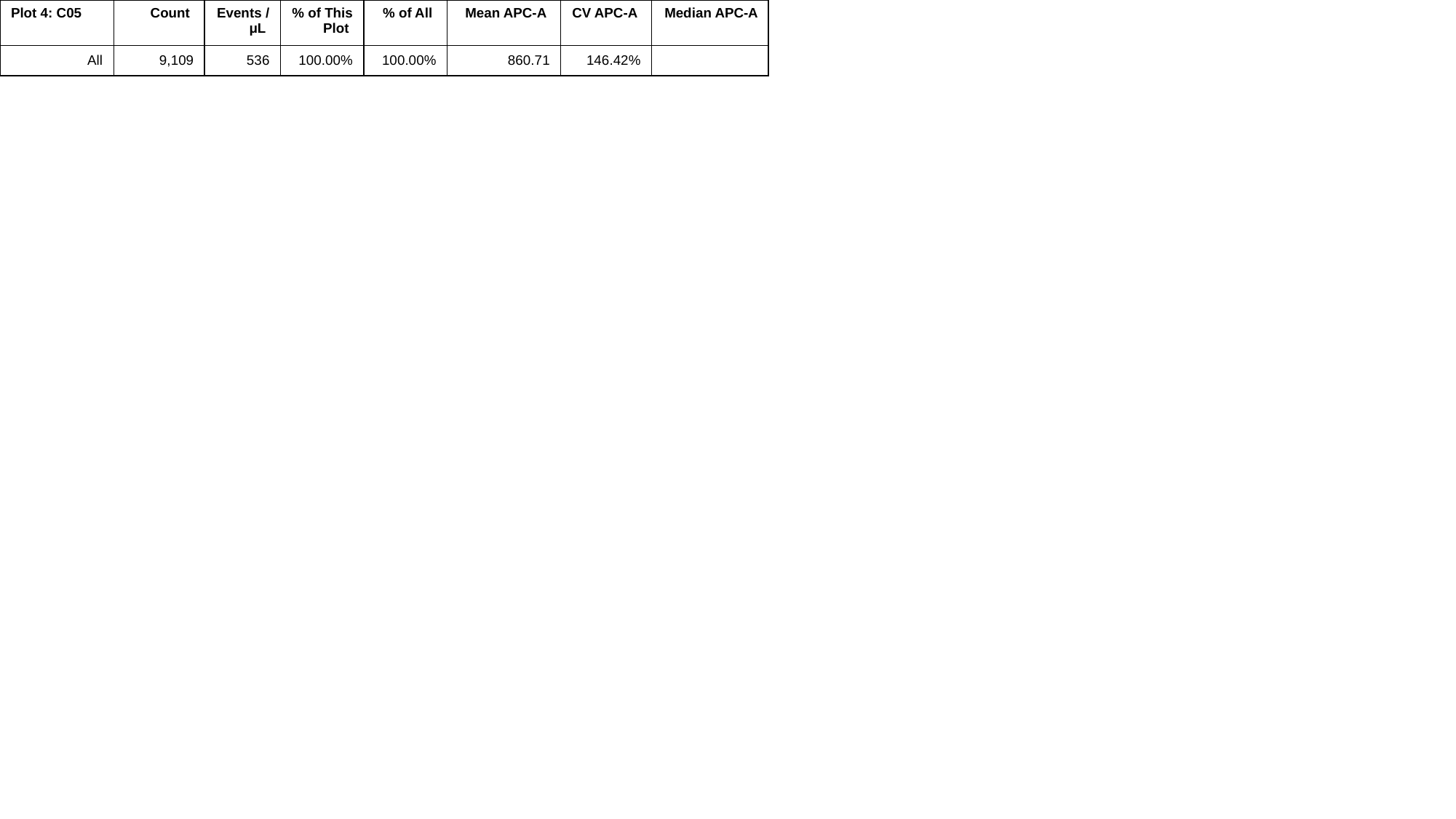

| Plot 4: C05 | Count | Events / μL | % of This Plot | % of All | Mean APC-A | CV APC-A | Median APC-A |
| --- | --- | --- | --- | --- | --- | --- | --- |
| All | 9,109 | 536 | 100.00% | 100.00% | 860.71 | 146.42% | |

## Slide 18
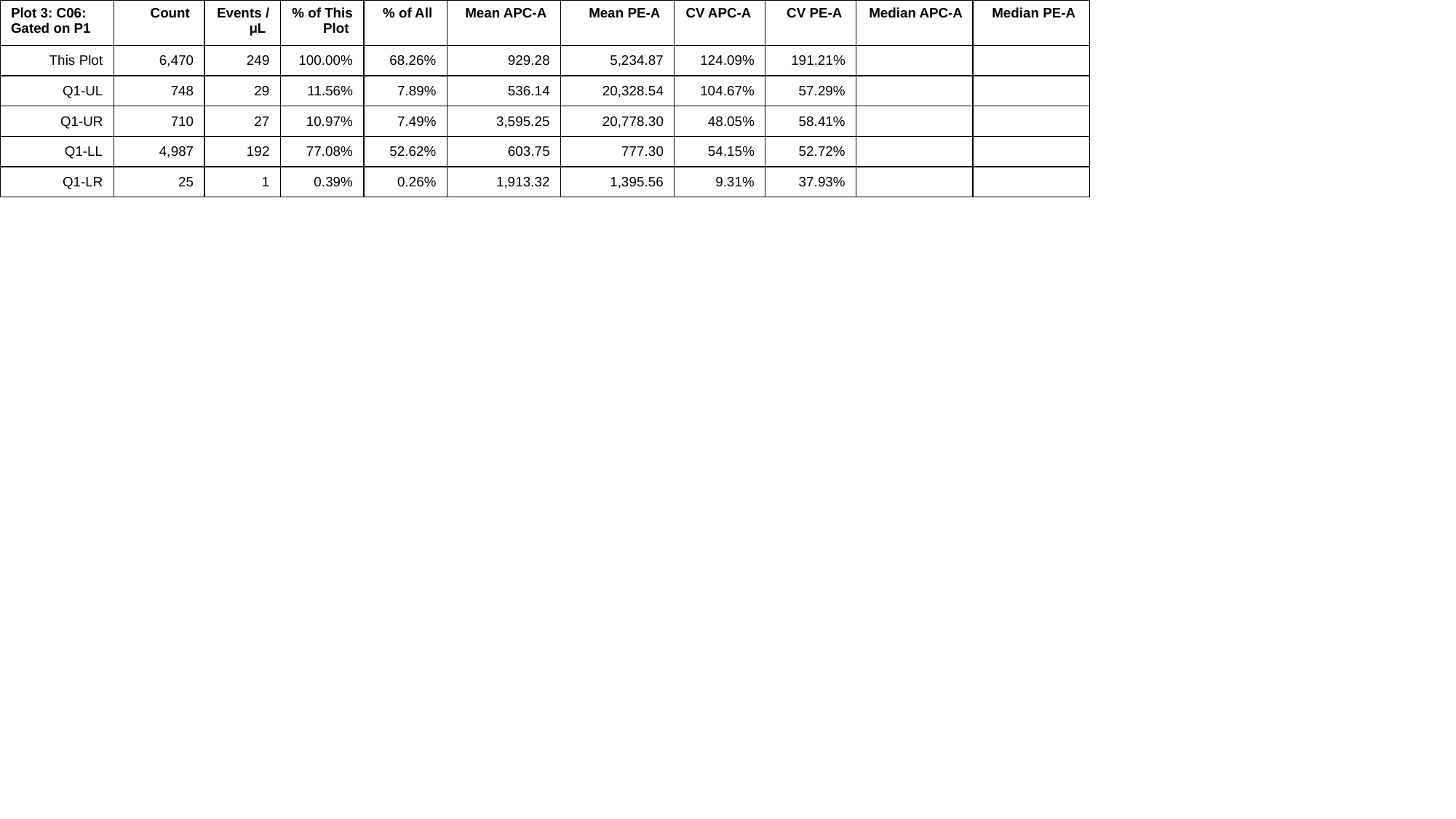

| Plot 3: C06: Gated on P1 | Count | Events / μL | % of This Plot | % of All | Mean APC-A | Mean PE-A | CV APC-A | CV PE-A | Median APC-A | Median PE-A |
| --- | --- | --- | --- | --- | --- | --- | --- | --- | --- | --- |
| This Plot | 6,470 | 249 | 100.00% | 68.26% | 929.28 | 5,234.87 | 124.09% | 191.21% | | |
| Q1-UL | 748 | 29 | 11.56% | 7.89% | 536.14 | 20,328.54 | 104.67% | 57.29% | | |
| Q1-UR | 710 | 27 | 10.97% | 7.49% | 3,595.25 | 20,778.30 | 48.05% | 58.41% | | |
| Q1-LL | 4,987 | 192 | 77.08% | 52.62% | 603.75 | 777.30 | 54.15% | 52.72% | | |
| Q1-LR | 25 | 1 | 0.39% | 0.26% | 1,913.32 | 1,395.56 | 9.31% | 37.93% | | |

## Slide 19
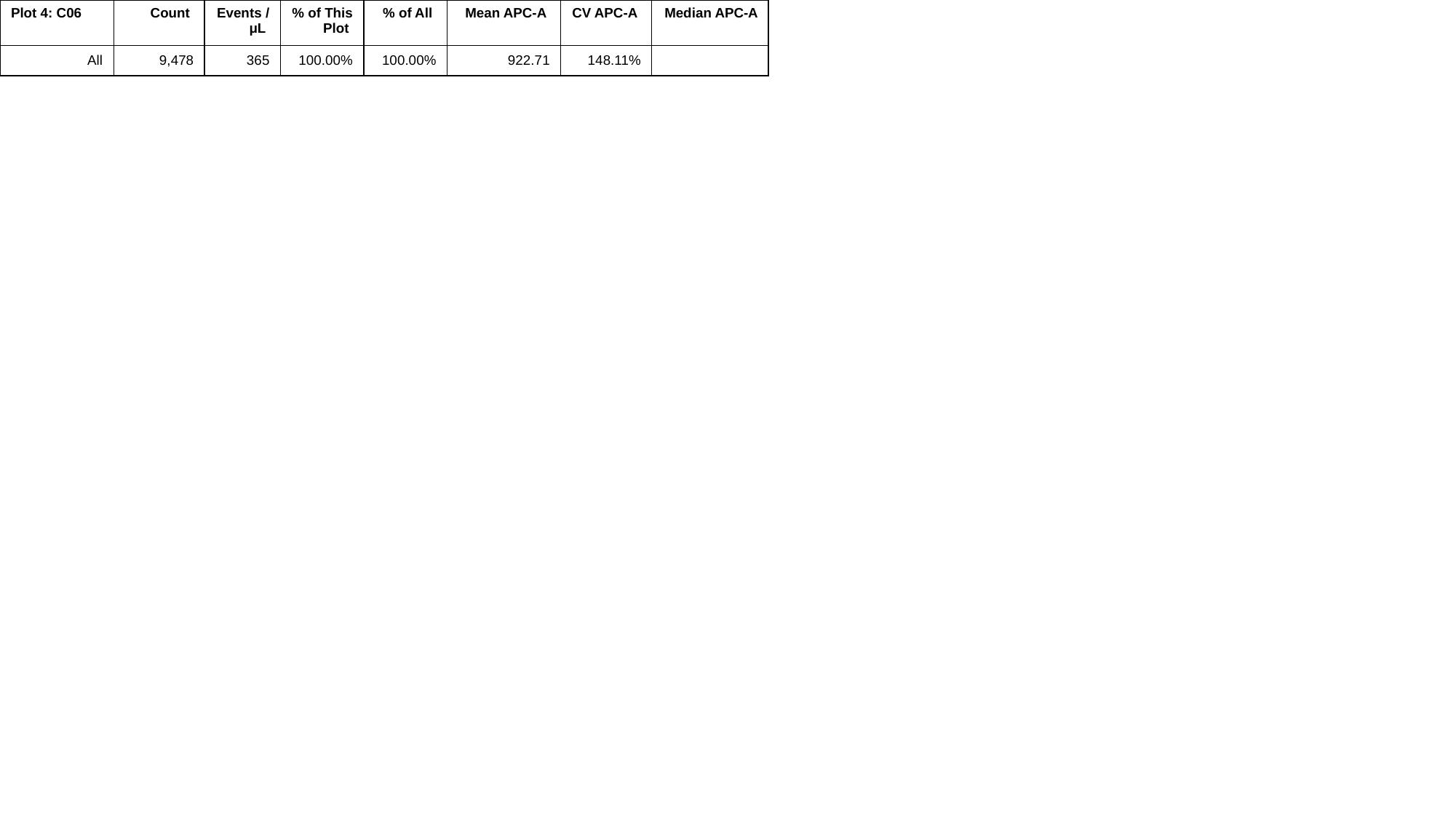

| Plot 4: C06 | Count | Events / μL | % of This Plot | % of All | Mean APC-A | CV APC-A | Median APC-A |
| --- | --- | --- | --- | --- | --- | --- | --- |
| All | 9,478 | 365 | 100.00% | 100.00% | 922.71 | 148.11% | |

## Slide 20
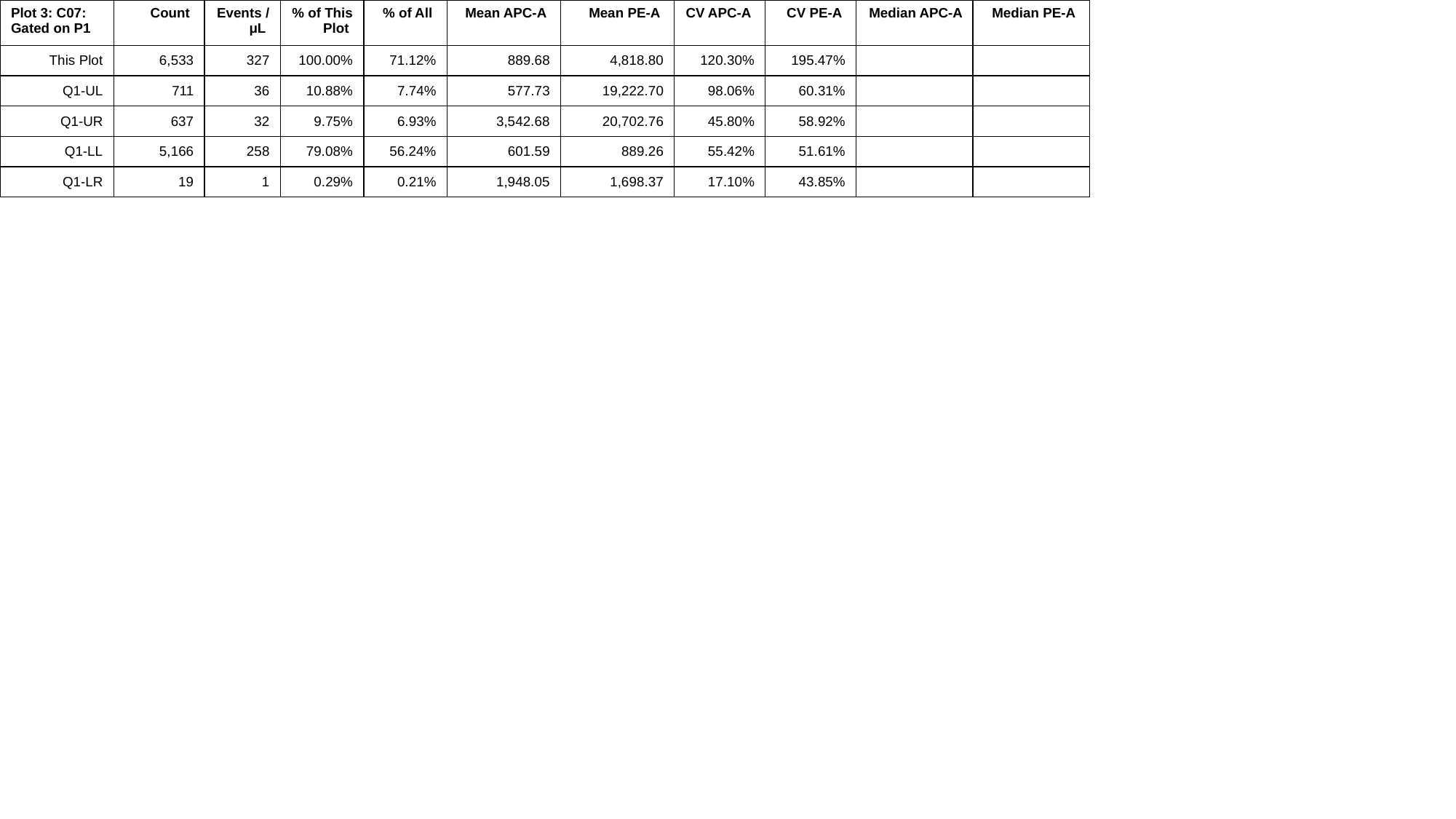

| Plot 3: C07: Gated on P1 | Count | Events / μL | % of This Plot | % of All | Mean APC-A | Mean PE-A | CV APC-A | CV PE-A | Median APC-A | Median PE-A |
| --- | --- | --- | --- | --- | --- | --- | --- | --- | --- | --- |
| This Plot | 6,533 | 327 | 100.00% | 71.12% | 889.68 | 4,818.80 | 120.30% | 195.47% | | |
| Q1-UL | 711 | 36 | 10.88% | 7.74% | 577.73 | 19,222.70 | 98.06% | 60.31% | | |
| Q1-UR | 637 | 32 | 9.75% | 6.93% | 3,542.68 | 20,702.76 | 45.80% | 58.92% | | |
| Q1-LL | 5,166 | 258 | 79.08% | 56.24% | 601.59 | 889.26 | 55.42% | 51.61% | | |
| Q1-LR | 19 | 1 | 0.29% | 0.21% | 1,948.05 | 1,698.37 | 17.10% | 43.85% | | |

## Slide 21
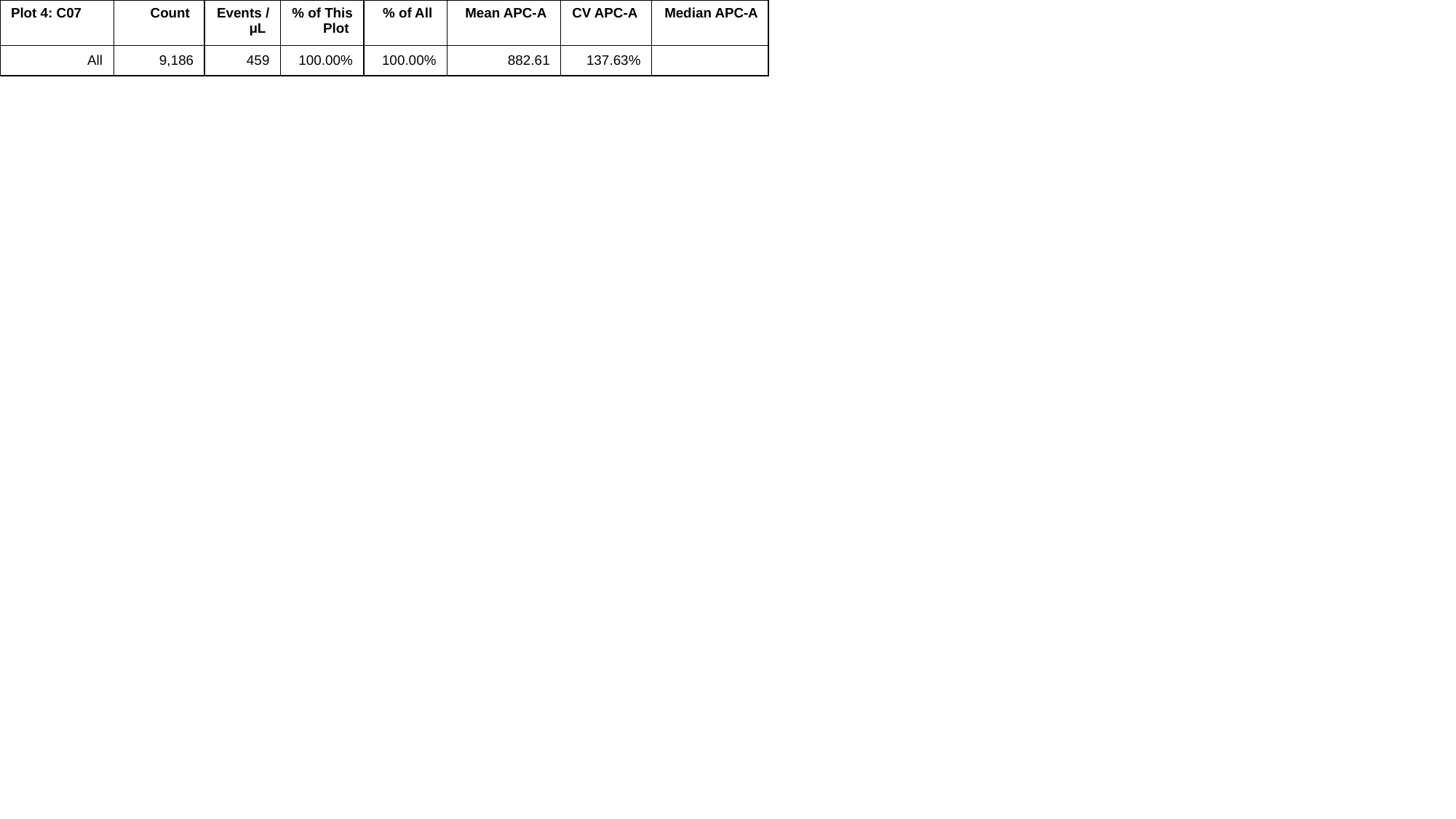

| Plot 4: C07 | Count | Events / μL | % of This Plot | % of All | Mean APC-A | CV APC-A | Median APC-A |
| --- | --- | --- | --- | --- | --- | --- | --- |
| All | 9,186 | 459 | 100.00% | 100.00% | 882.61 | 137.63% | |

## Slide 22
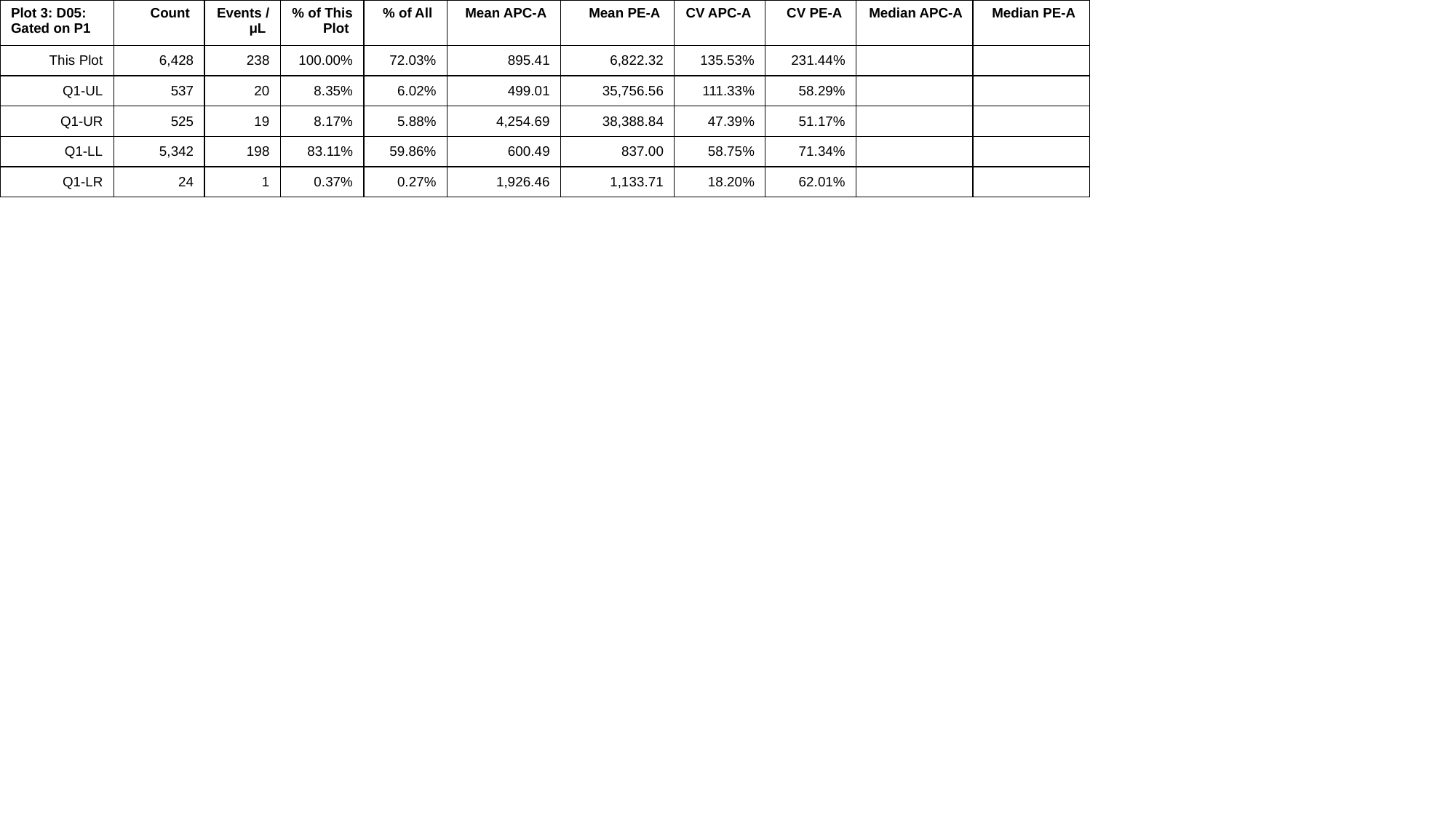

| Plot 3: D05: Gated on P1 | Count | Events / μL | % of This Plot | % of All | Mean APC-A | Mean PE-A | CV APC-A | CV PE-A | Median APC-A | Median PE-A |
| --- | --- | --- | --- | --- | --- | --- | --- | --- | --- | --- |
| This Plot | 6,428 | 238 | 100.00% | 72.03% | 895.41 | 6,822.32 | 135.53% | 231.44% | | |
| Q1-UL | 537 | 20 | 8.35% | 6.02% | 499.01 | 35,756.56 | 111.33% | 58.29% | | |
| Q1-UR | 525 | 19 | 8.17% | 5.88% | 4,254.69 | 38,388.84 | 47.39% | 51.17% | | |
| Q1-LL | 5,342 | 198 | 83.11% | 59.86% | 600.49 | 837.00 | 58.75% | 71.34% | | |
| Q1-LR | 24 | 1 | 0.37% | 0.27% | 1,926.46 | 1,133.71 | 18.20% | 62.01% | | |

## Slide 23
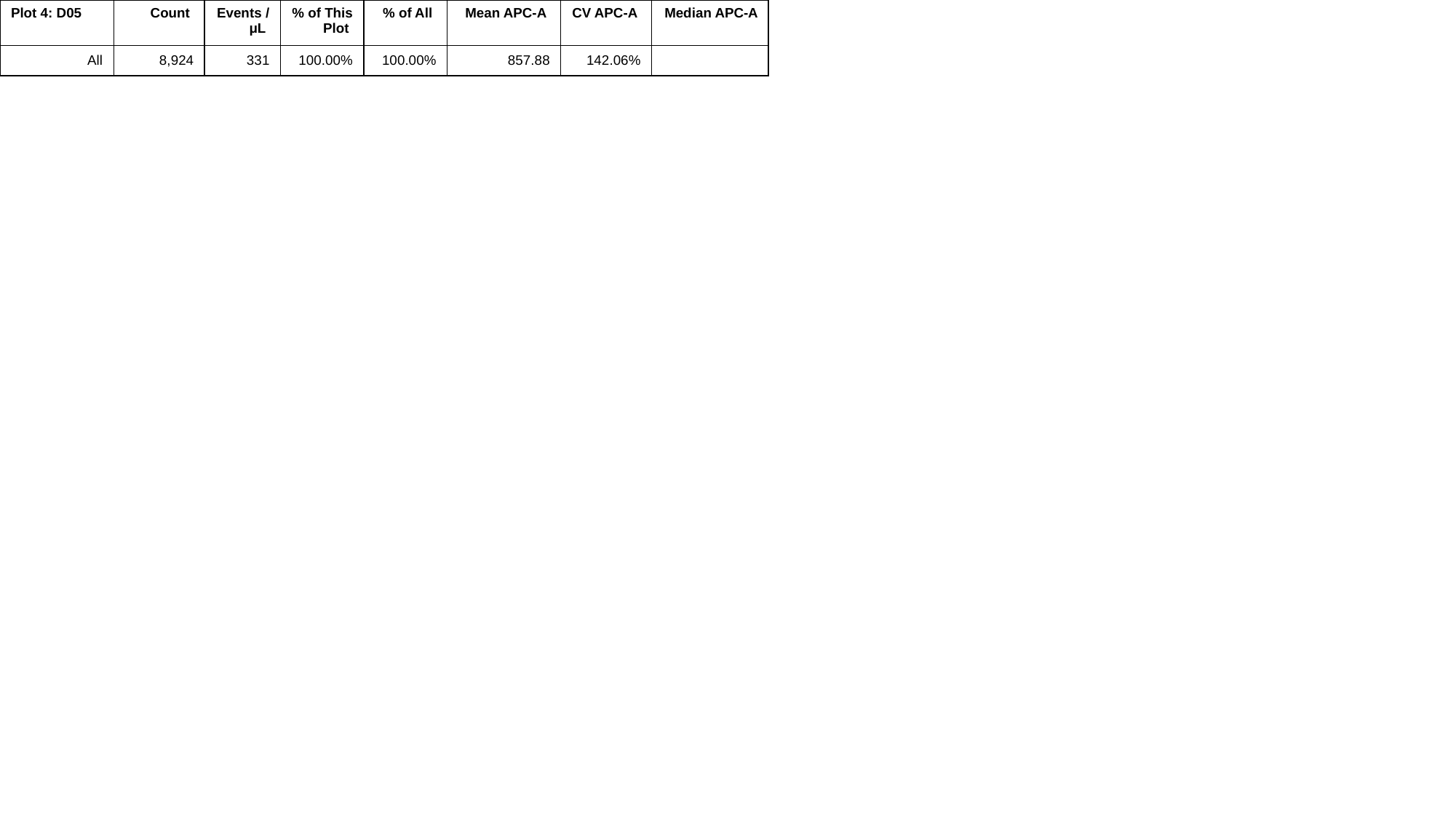

| Plot 4: D05 | Count | Events / μL | % of This Plot | % of All | Mean APC-A | CV APC-A | Median APC-A |
| --- | --- | --- | --- | --- | --- | --- | --- |
| All | 8,924 | 331 | 100.00% | 100.00% | 857.88 | 142.06% | |

## Slide 24
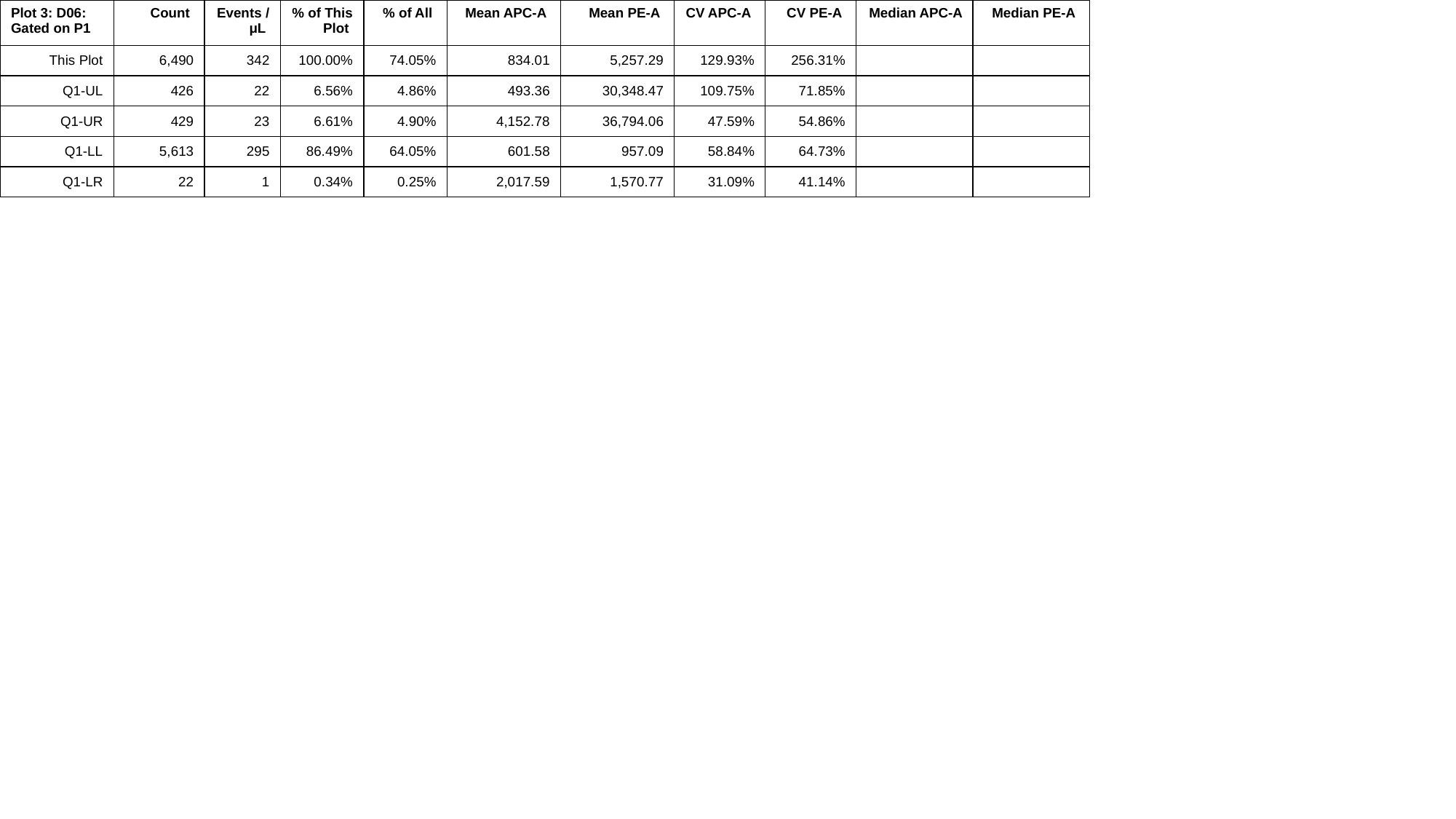

| Plot 3: D06: Gated on P1 | Count | Events / μL | % of This Plot | % of All | Mean APC-A | Mean PE-A | CV APC-A | CV PE-A | Median APC-A | Median PE-A |
| --- | --- | --- | --- | --- | --- | --- | --- | --- | --- | --- |
| This Plot | 6,490 | 342 | 100.00% | 74.05% | 834.01 | 5,257.29 | 129.93% | 256.31% | | |
| Q1-UL | 426 | 22 | 6.56% | 4.86% | 493.36 | 30,348.47 | 109.75% | 71.85% | | |
| Q1-UR | 429 | 23 | 6.61% | 4.90% | 4,152.78 | 36,794.06 | 47.59% | 54.86% | | |
| Q1-LL | 5,613 | 295 | 86.49% | 64.05% | 601.58 | 957.09 | 58.84% | 64.73% | | |
| Q1-LR | 22 | 1 | 0.34% | 0.25% | 2,017.59 | 1,570.77 | 31.09% | 41.14% | | |

## Slide 25
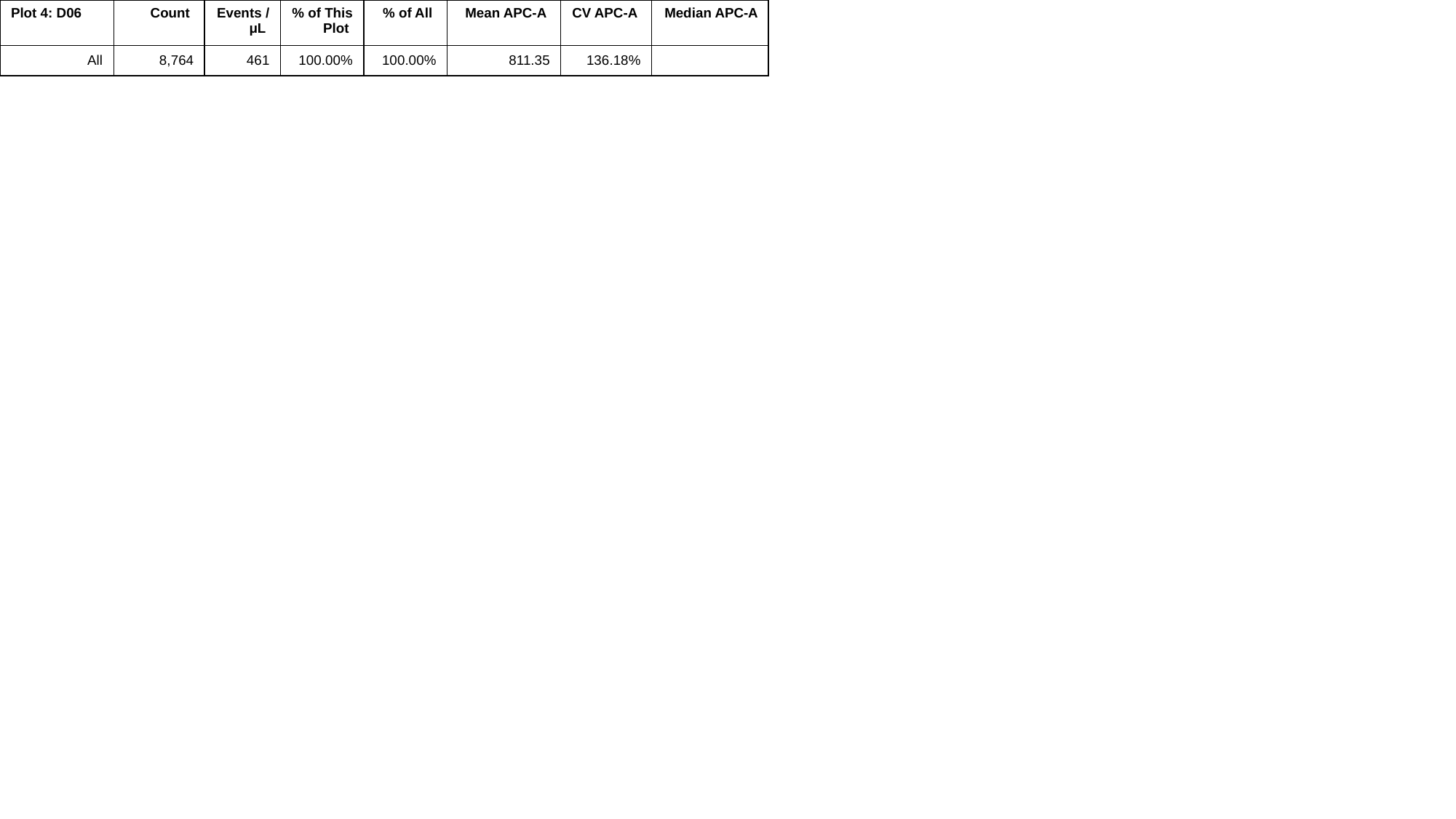

| Plot 4: D06 | Count | Events / μL | % of This Plot | % of All | Mean APC-A | CV APC-A | Median APC-A |
| --- | --- | --- | --- | --- | --- | --- | --- |
| All | 8,764 | 461 | 100.00% | 100.00% | 811.35 | 136.18% | |

## Slide 26
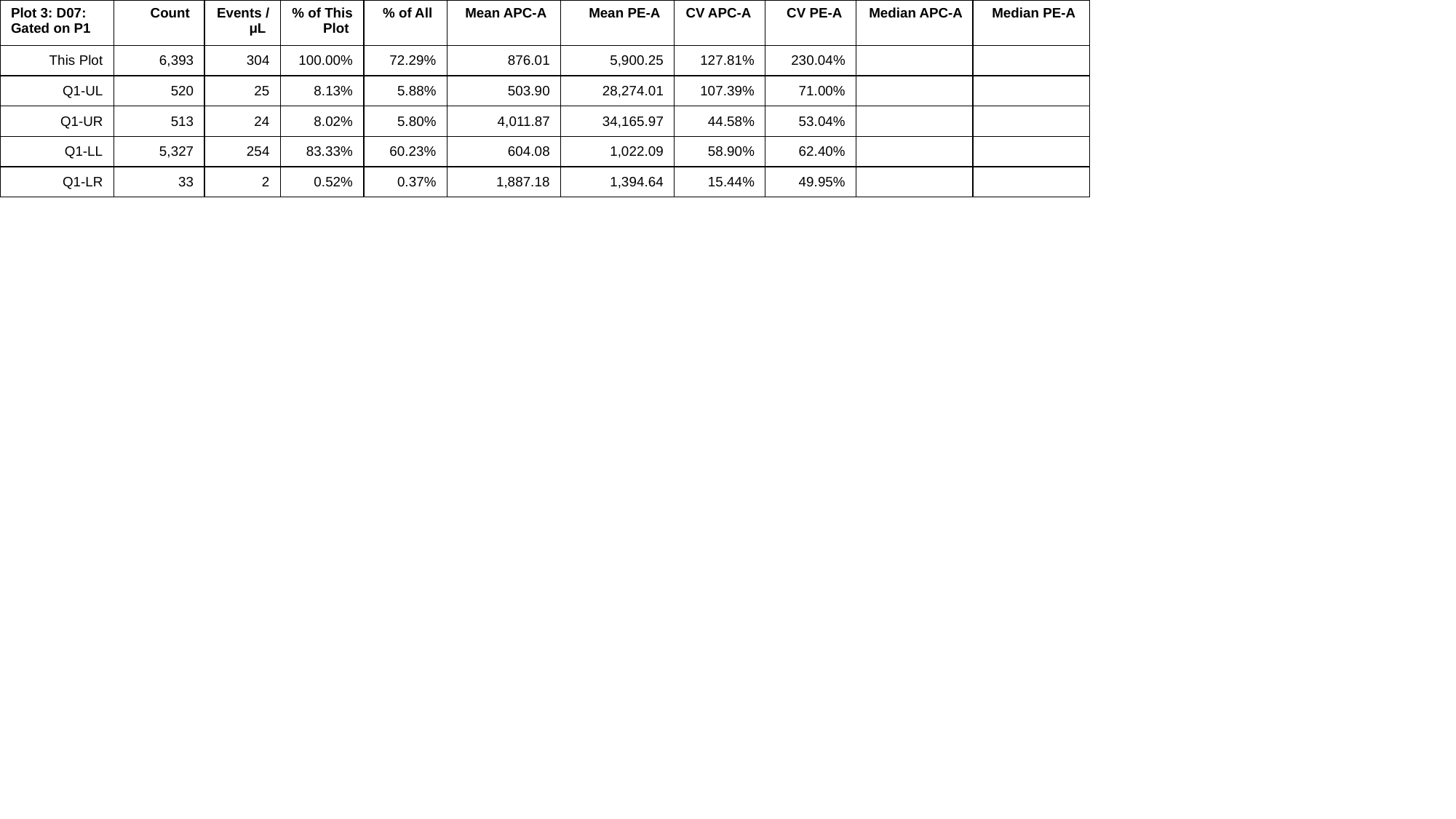

| Plot 3: D07: Gated on P1 | Count | Events / μL | % of This Plot | % of All | Mean APC-A | Mean PE-A | CV APC-A | CV PE-A | Median APC-A | Median PE-A |
| --- | --- | --- | --- | --- | --- | --- | --- | --- | --- | --- |
| This Plot | 6,393 | 304 | 100.00% | 72.29% | 876.01 | 5,900.25 | 127.81% | 230.04% | | |
| Q1-UL | 520 | 25 | 8.13% | 5.88% | 503.90 | 28,274.01 | 107.39% | 71.00% | | |
| Q1-UR | 513 | 24 | 8.02% | 5.80% | 4,011.87 | 34,165.97 | 44.58% | 53.04% | | |
| Q1-LL | 5,327 | 254 | 83.33% | 60.23% | 604.08 | 1,022.09 | 58.90% | 62.40% | | |
| Q1-LR | 33 | 2 | 0.52% | 0.37% | 1,887.18 | 1,394.64 | 15.44% | 49.95% | | |

## Slide 27
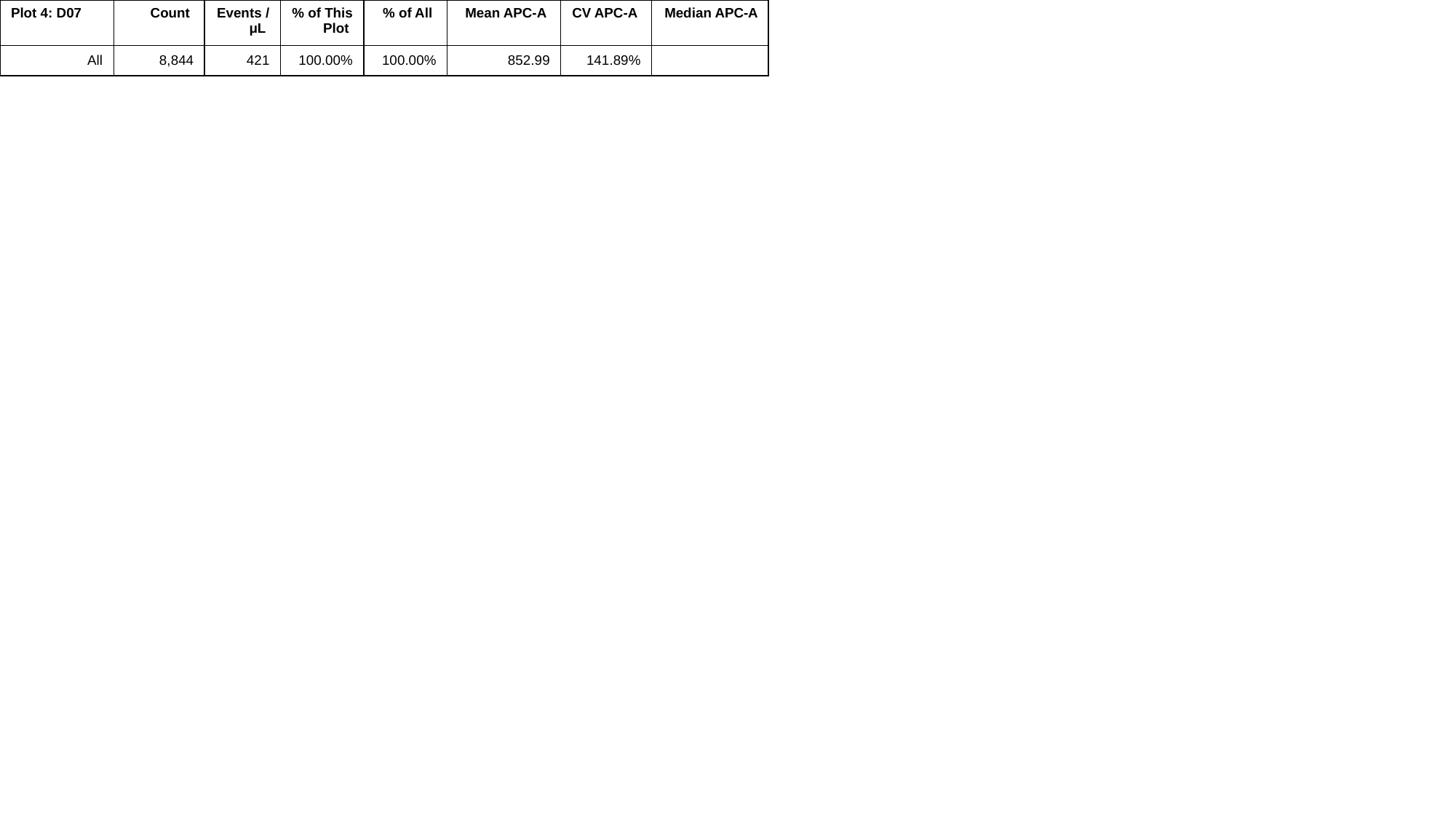

| Plot 4: D07 | Count | Events / μL | % of This Plot | % of All | Mean APC-A | CV APC-A | Median APC-A |
| --- | --- | --- | --- | --- | --- | --- | --- |
| All | 8,844 | 421 | 100.00% | 100.00% | 852.99 | 141.89% | |

## Slide 28
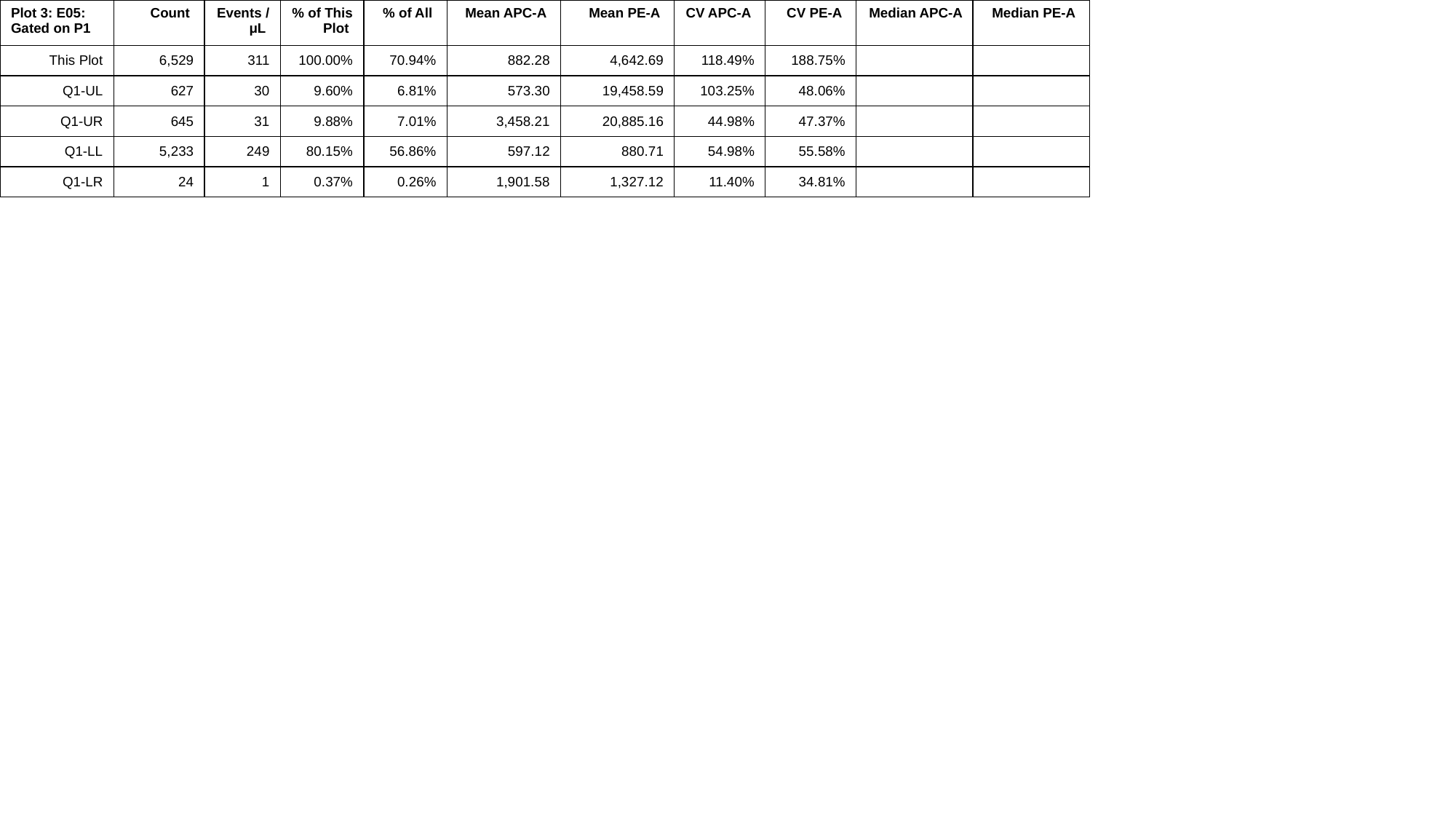

| Plot 3: E05: Gated on P1 | Count | Events / μL | % of This Plot | % of All | Mean APC-A | Mean PE-A | CV APC-A | CV PE-A | Median APC-A | Median PE-A |
| --- | --- | --- | --- | --- | --- | --- | --- | --- | --- | --- |
| This Plot | 6,529 | 311 | 100.00% | 70.94% | 882.28 | 4,642.69 | 118.49% | 188.75% | | |
| Q1-UL | 627 | 30 | 9.60% | 6.81% | 573.30 | 19,458.59 | 103.25% | 48.06% | | |
| Q1-UR | 645 | 31 | 9.88% | 7.01% | 3,458.21 | 20,885.16 | 44.98% | 47.37% | | |
| Q1-LL | 5,233 | 249 | 80.15% | 56.86% | 597.12 | 880.71 | 54.98% | 55.58% | | |
| Q1-LR | 24 | 1 | 0.37% | 0.26% | 1,901.58 | 1,327.12 | 11.40% | 34.81% | | |

## Slide 29
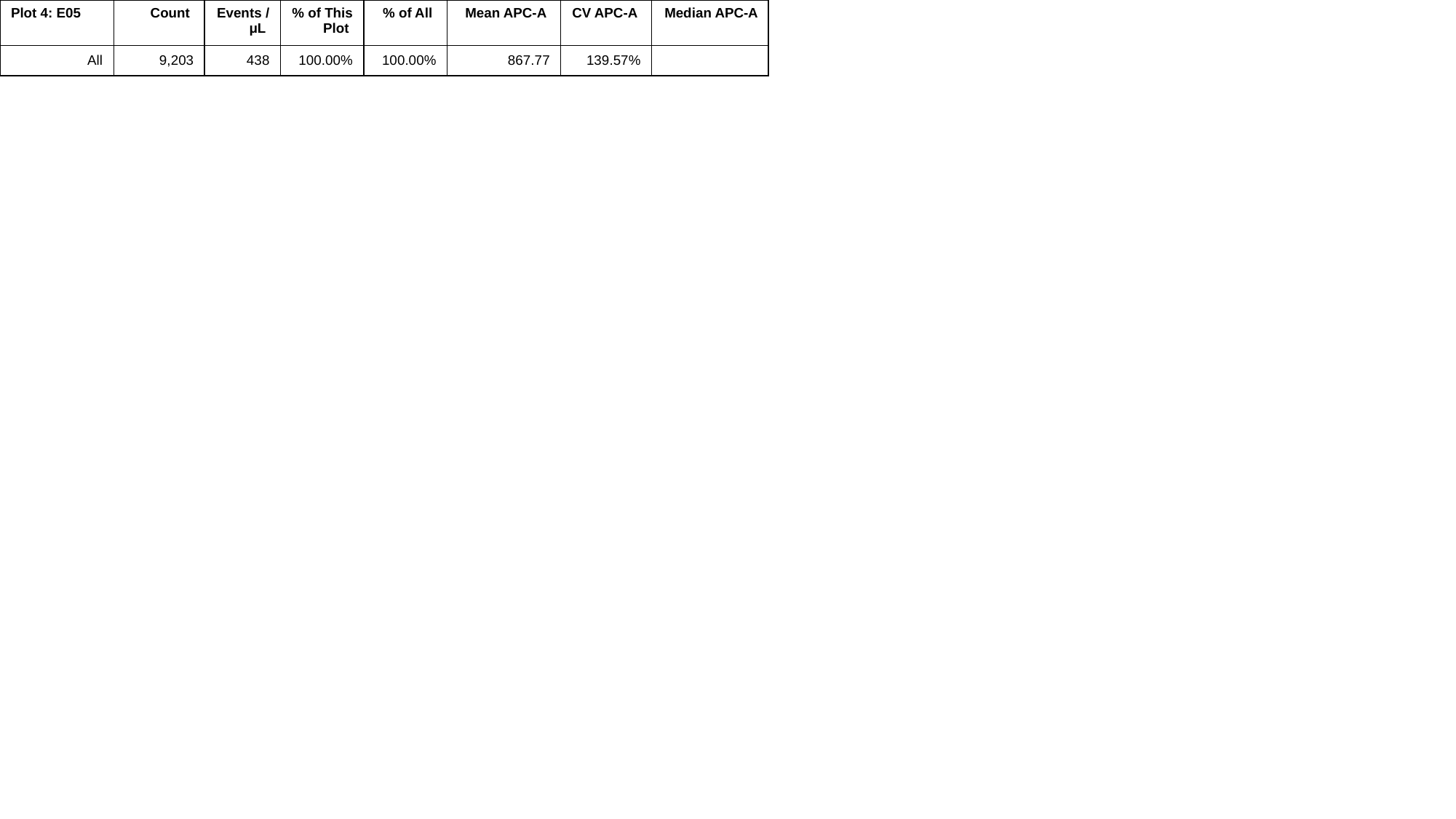

| Plot 4: E05 | Count | Events / μL | % of This Plot | % of All | Mean APC-A | CV APC-A | Median APC-A |
| --- | --- | --- | --- | --- | --- | --- | --- |
| All | 9,203 | 438 | 100.00% | 100.00% | 867.77 | 139.57% | |

## Slide 30
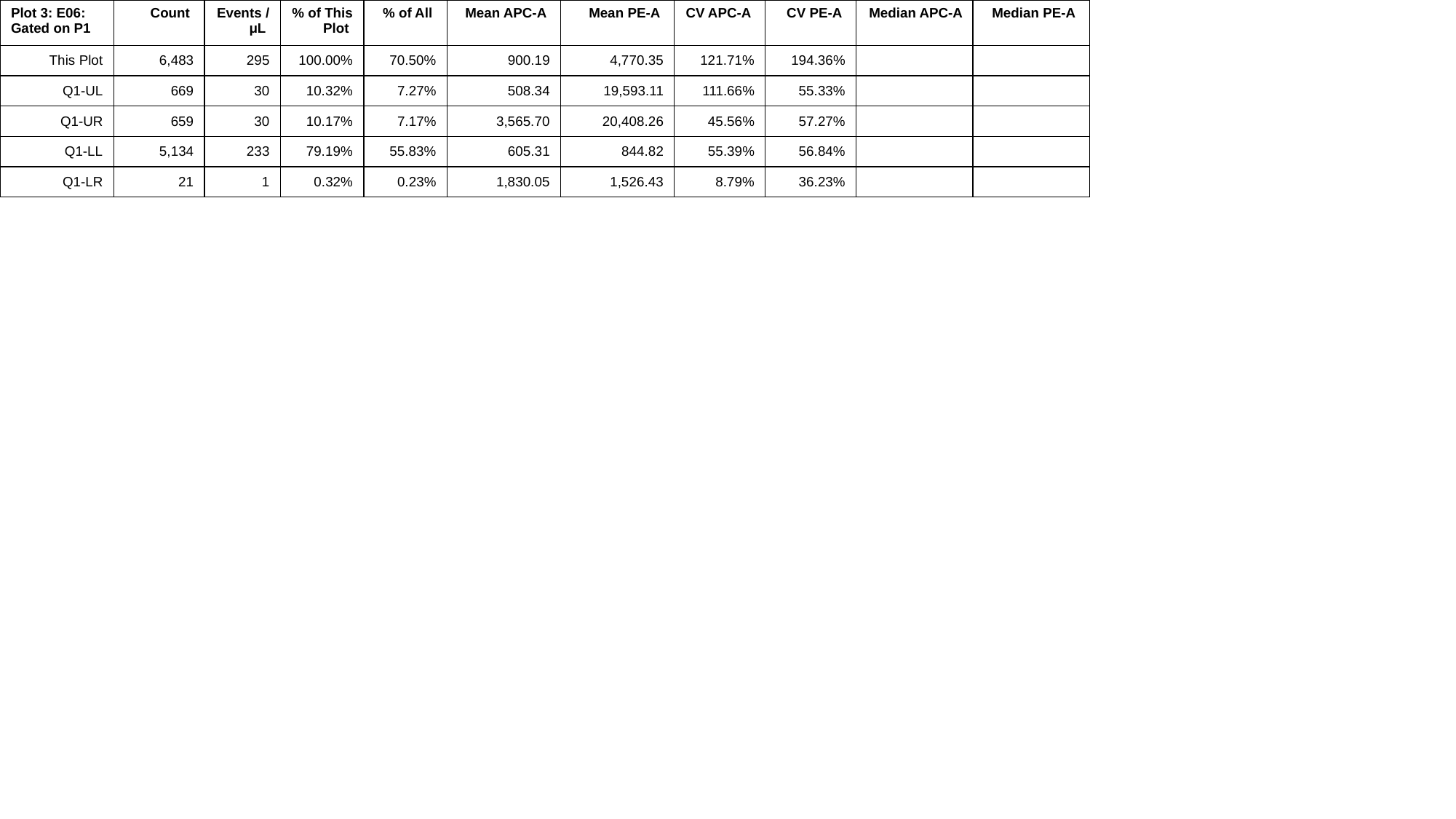

| Plot 3: E06: Gated on P1 | Count | Events / μL | % of This Plot | % of All | Mean APC-A | Mean PE-A | CV APC-A | CV PE-A | Median APC-A | Median PE-A |
| --- | --- | --- | --- | --- | --- | --- | --- | --- | --- | --- |
| This Plot | 6,483 | 295 | 100.00% | 70.50% | 900.19 | 4,770.35 | 121.71% | 194.36% | | |
| Q1-UL | 669 | 30 | 10.32% | 7.27% | 508.34 | 19,593.11 | 111.66% | 55.33% | | |
| Q1-UR | 659 | 30 | 10.17% | 7.17% | 3,565.70 | 20,408.26 | 45.56% | 57.27% | | |
| Q1-LL | 5,134 | 233 | 79.19% | 55.83% | 605.31 | 844.82 | 55.39% | 56.84% | | |
| Q1-LR | 21 | 1 | 0.32% | 0.23% | 1,830.05 | 1,526.43 | 8.79% | 36.23% | | |

## Slide 31
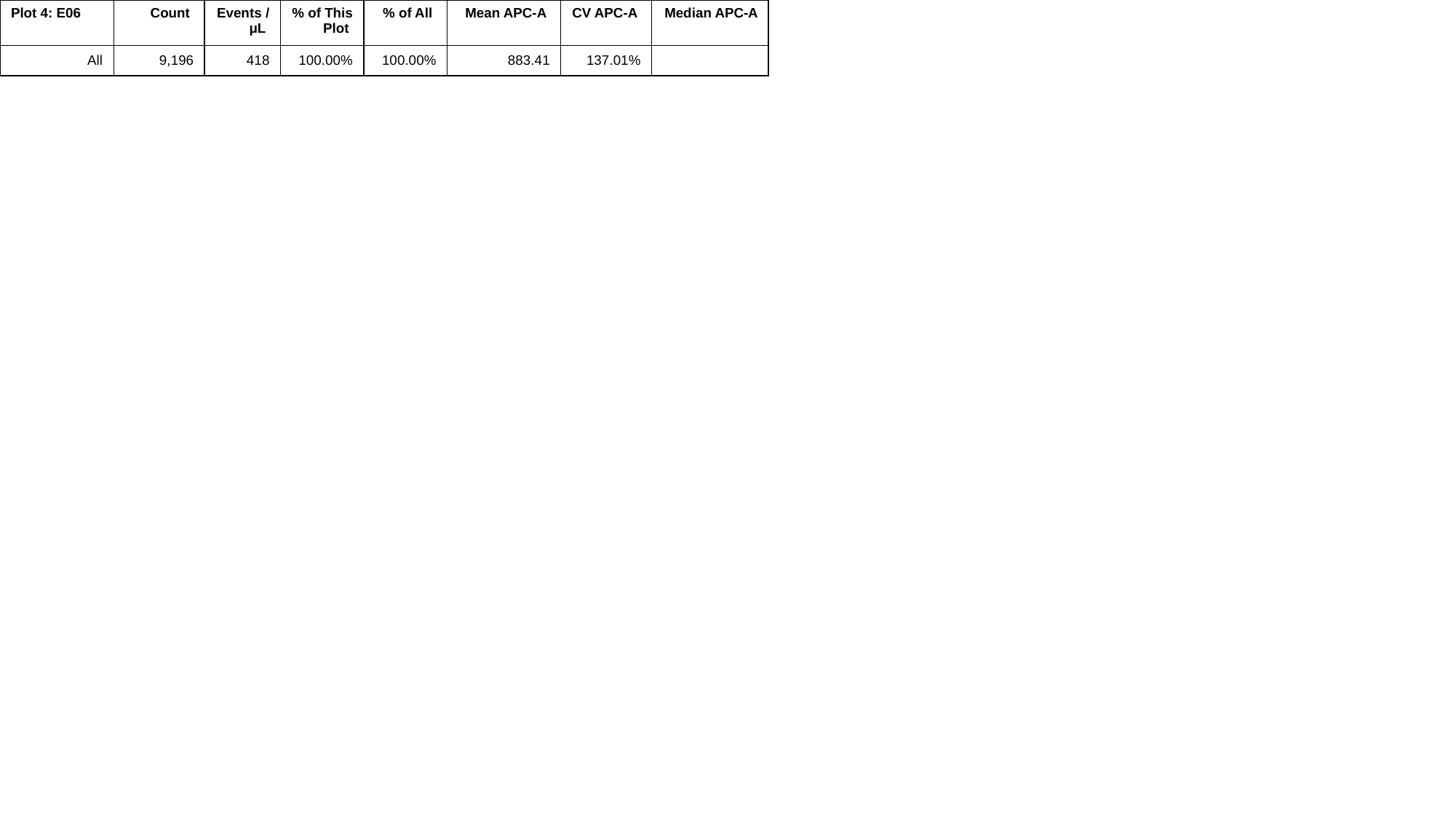

| Plot 4: E06 | Count | Events / μL | % of This Plot | % of All | Mean APC-A | CV APC-A | Median APC-A |
| --- | --- | --- | --- | --- | --- | --- | --- |
| All | 9,196 | 418 | 100.00% | 100.00% | 883.41 | 137.01% | |

## Slide 32
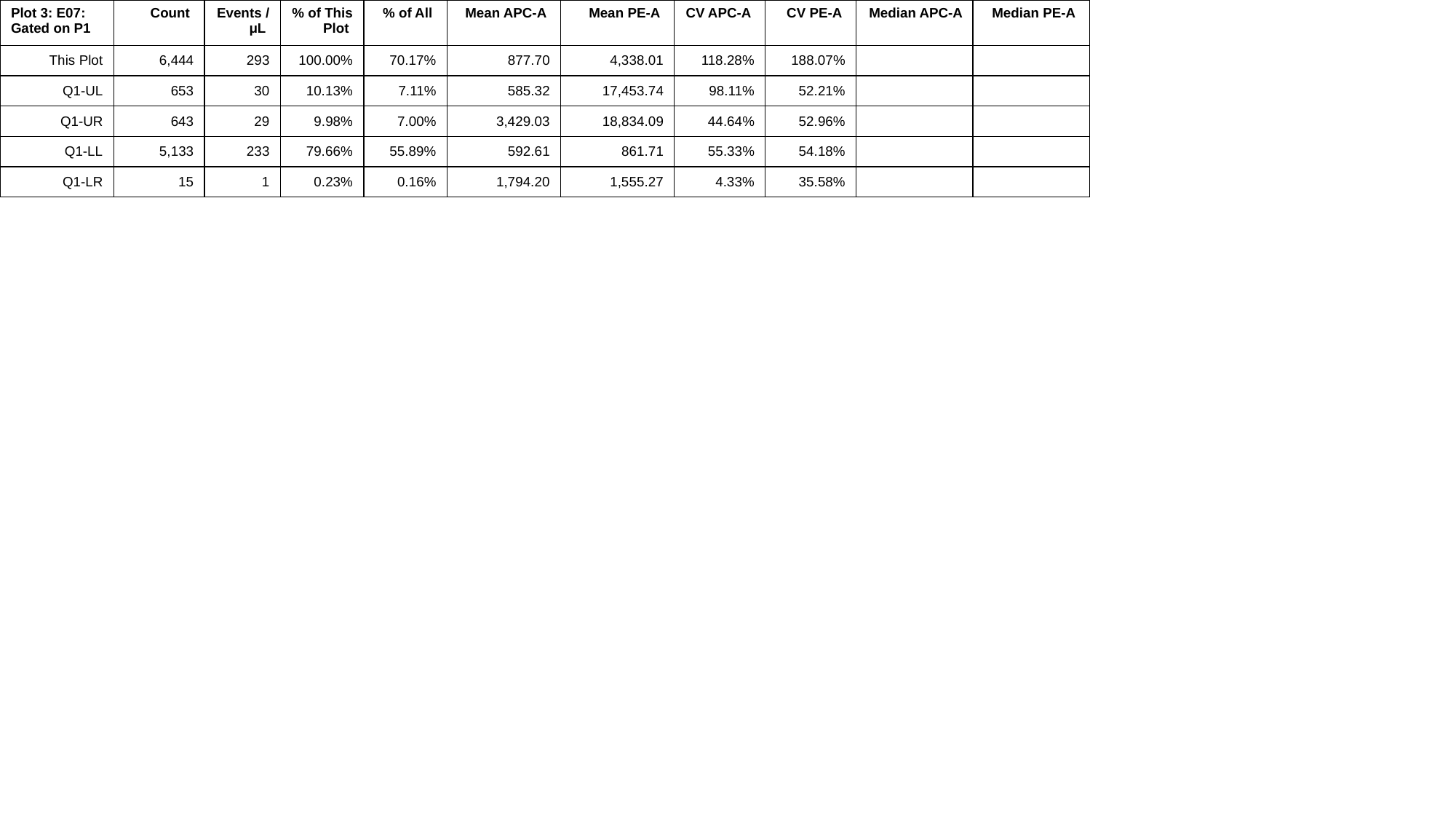

| Plot 3: E07: Gated on P1 | Count | Events / μL | % of This Plot | % of All | Mean APC-A | Mean PE-A | CV APC-A | CV PE-A | Median APC-A | Median PE-A |
| --- | --- | --- | --- | --- | --- | --- | --- | --- | --- | --- |
| This Plot | 6,444 | 293 | 100.00% | 70.17% | 877.70 | 4,338.01 | 118.28% | 188.07% | | |
| Q1-UL | 653 | 30 | 10.13% | 7.11% | 585.32 | 17,453.74 | 98.11% | 52.21% | | |
| Q1-UR | 643 | 29 | 9.98% | 7.00% | 3,429.03 | 18,834.09 | 44.64% | 52.96% | | |
| Q1-LL | 5,133 | 233 | 79.66% | 55.89% | 592.61 | 861.71 | 55.33% | 54.18% | | |
| Q1-LR | 15 | 1 | 0.23% | 0.16% | 1,794.20 | 1,555.27 | 4.33% | 35.58% | | |

## Slide 33
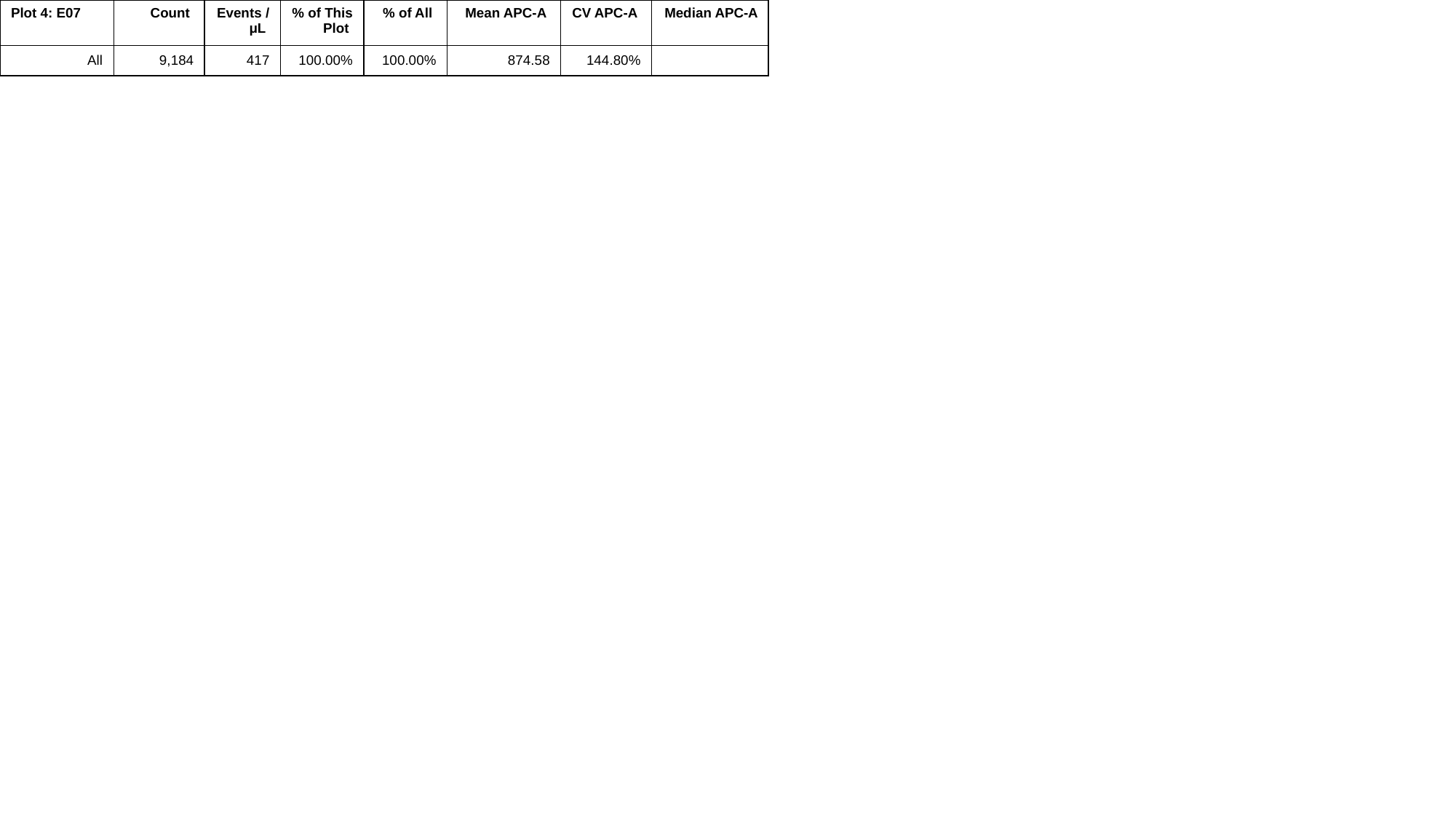

| Plot 4: E07 | Count | Events / μL | % of This Plot | % of All | Mean APC-A | CV APC-A | Median APC-A |
| --- | --- | --- | --- | --- | --- | --- | --- |
| All | 9,184 | 417 | 100.00% | 100.00% | 874.58 | 144.80% | |
