# Supplementary material for: CNN2 silencing inhibits colorectal cancer development through promoting ubiquitination of EGR1
Source: Life Sci Alliance. 2023 May 15;6(7):e202201639. doi: 10.26508/lsa.202201639 (PMC10185810; doi:10.26508/lsa.202201639)
Supplement: Supplementary file 5 [file LSA-2022-01639_SdataF2.3.pptx]

## Slide 1
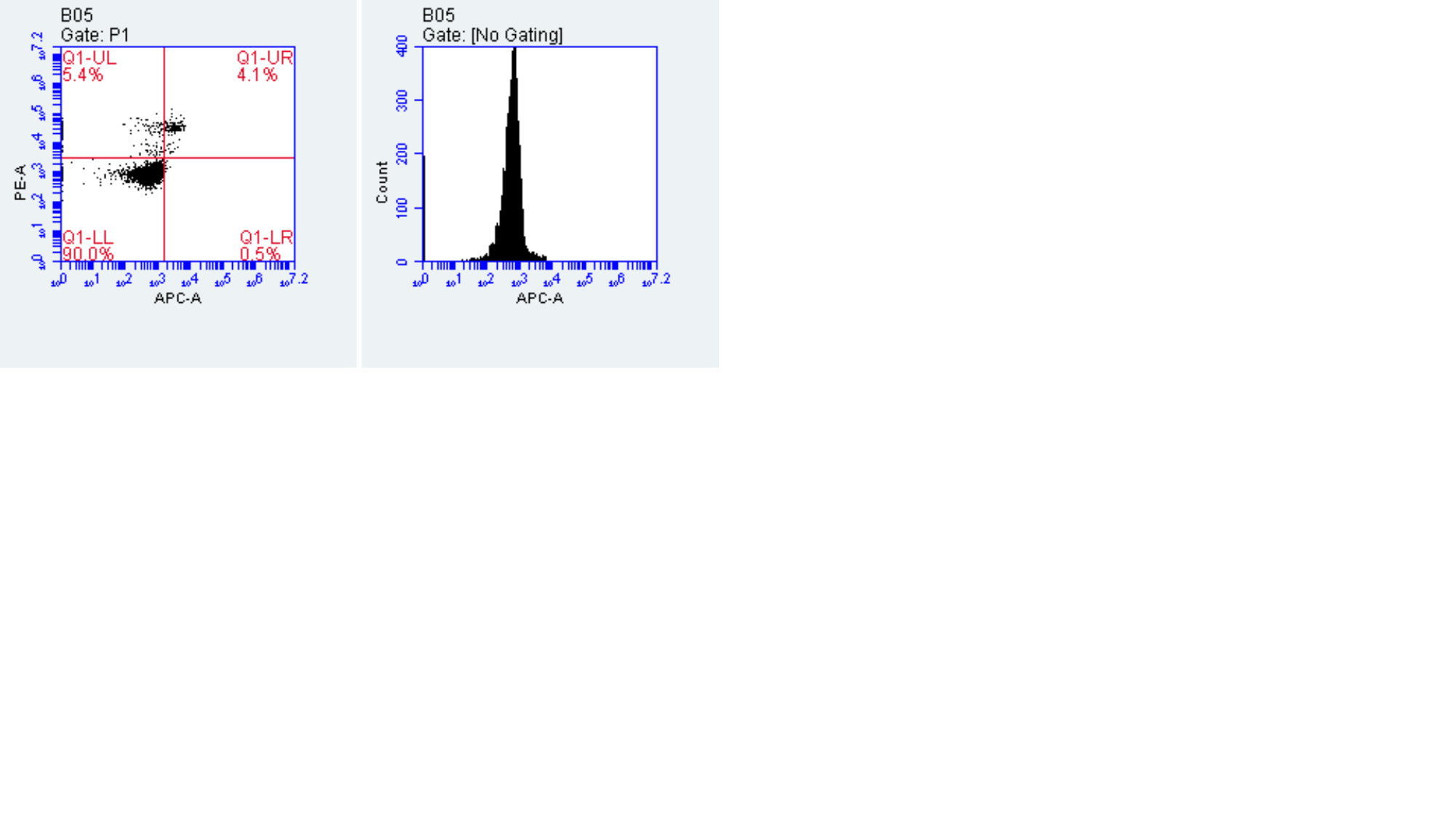

## Slide 2
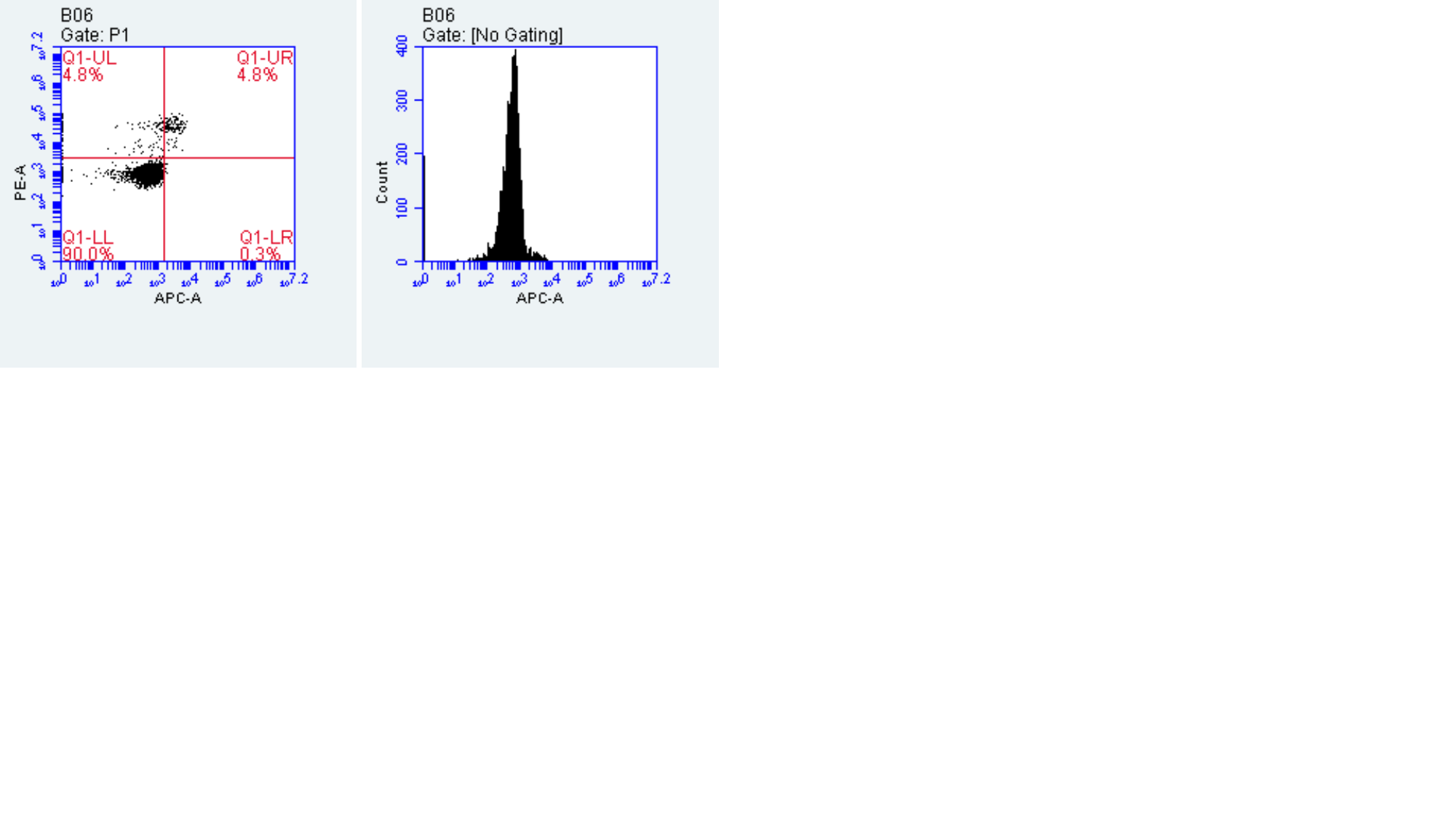

## Slide 3
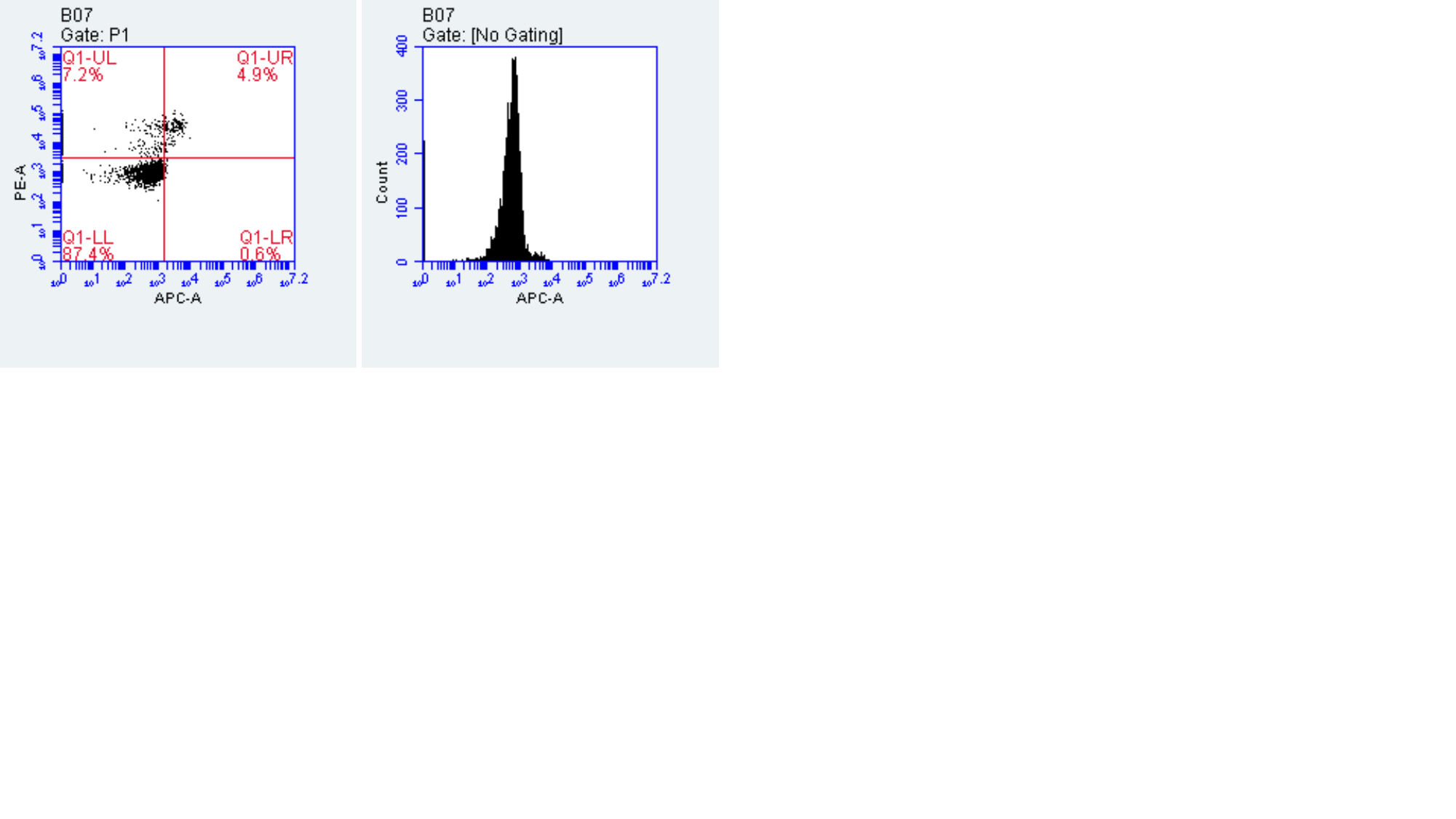

## Slide 4
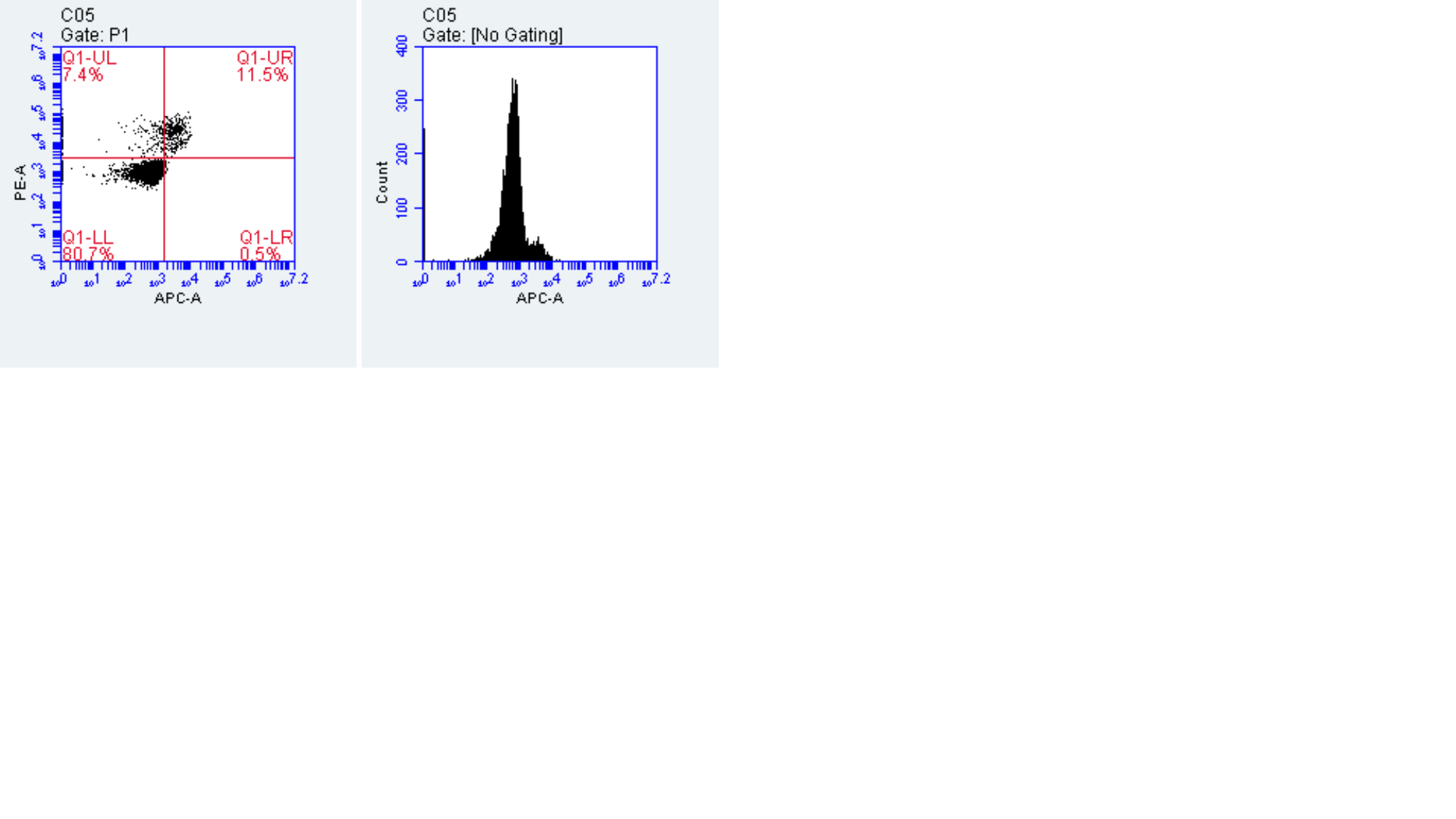

## Slide 5
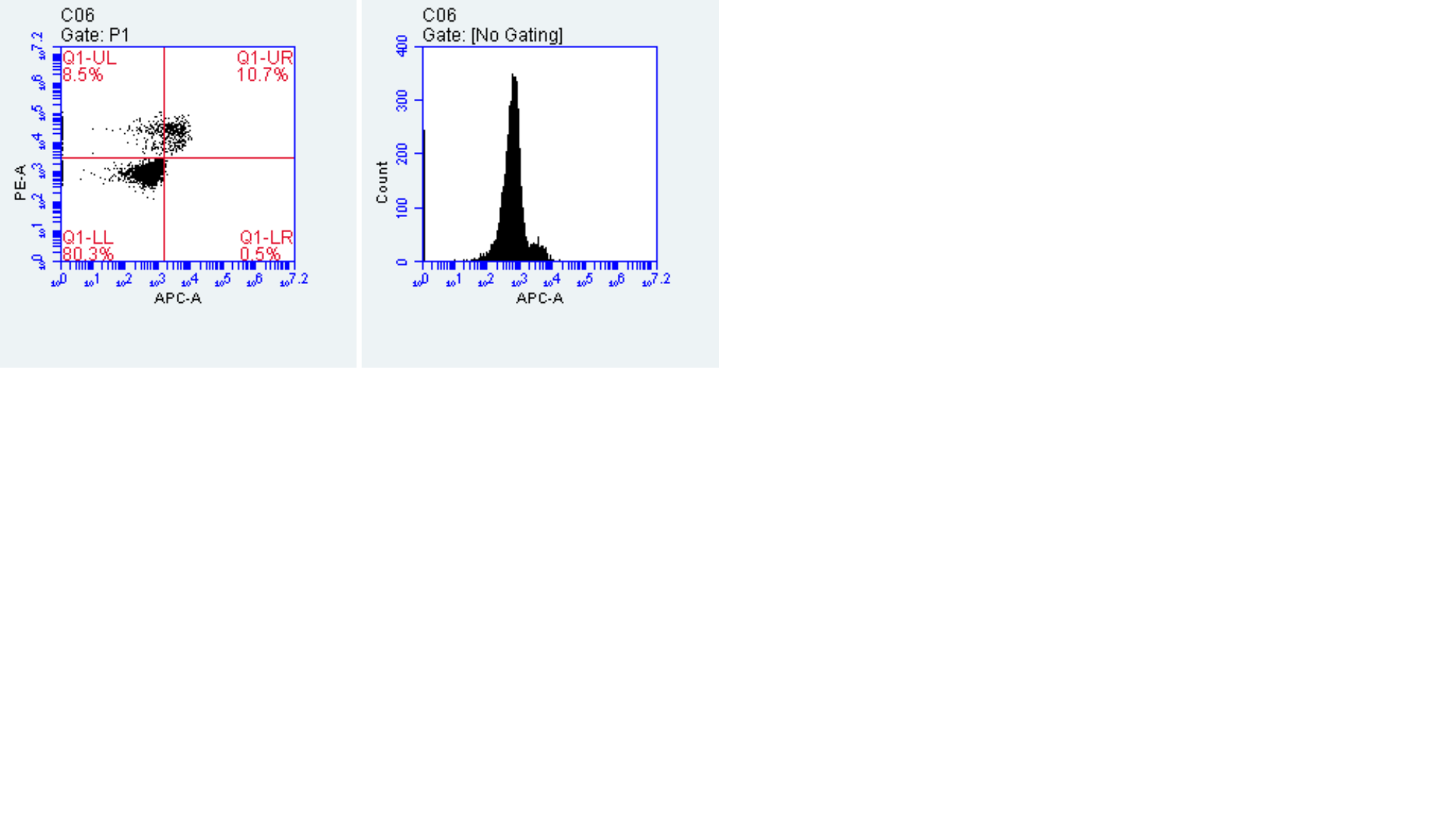

## Slide 6
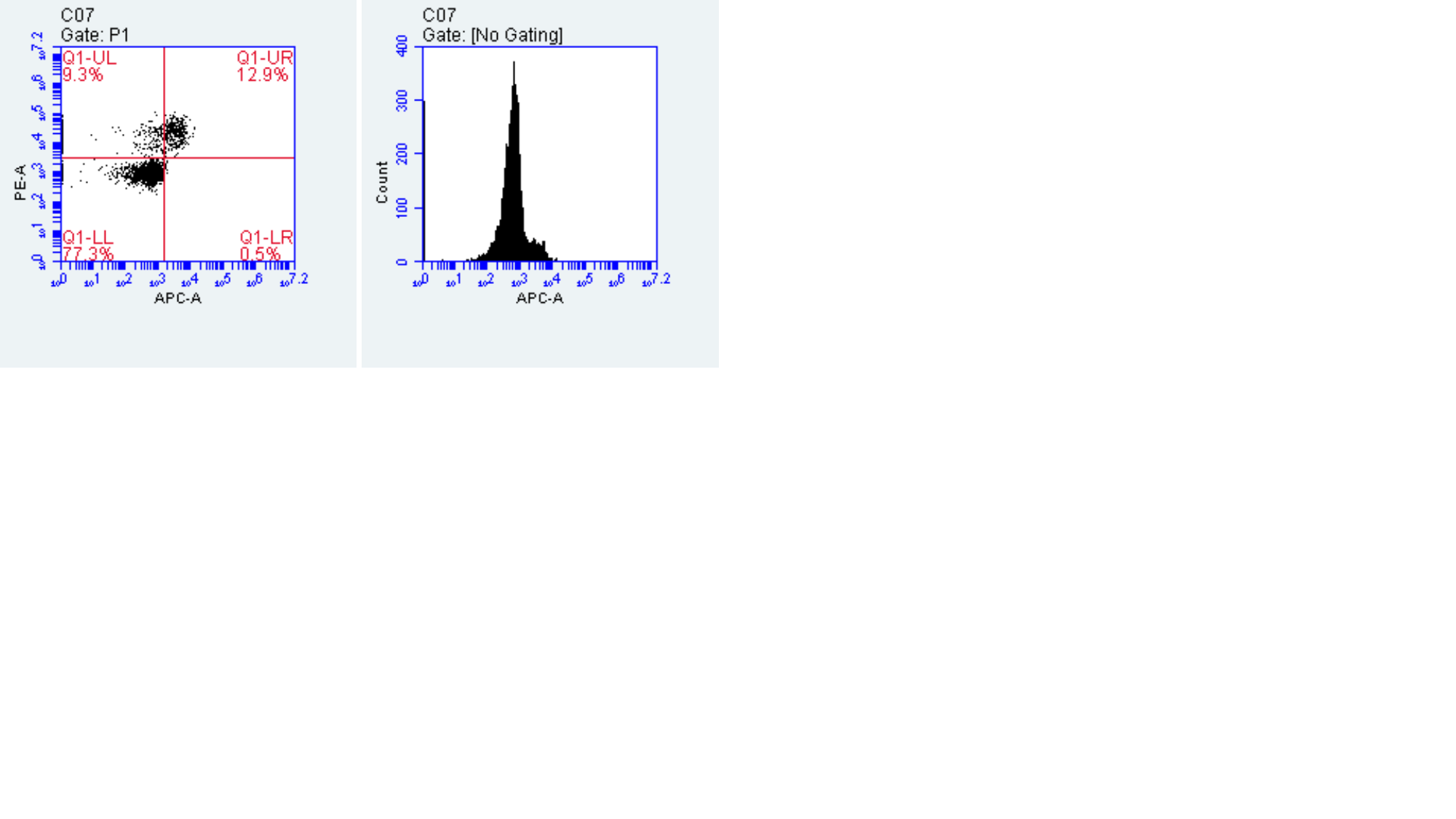

## Slide 7
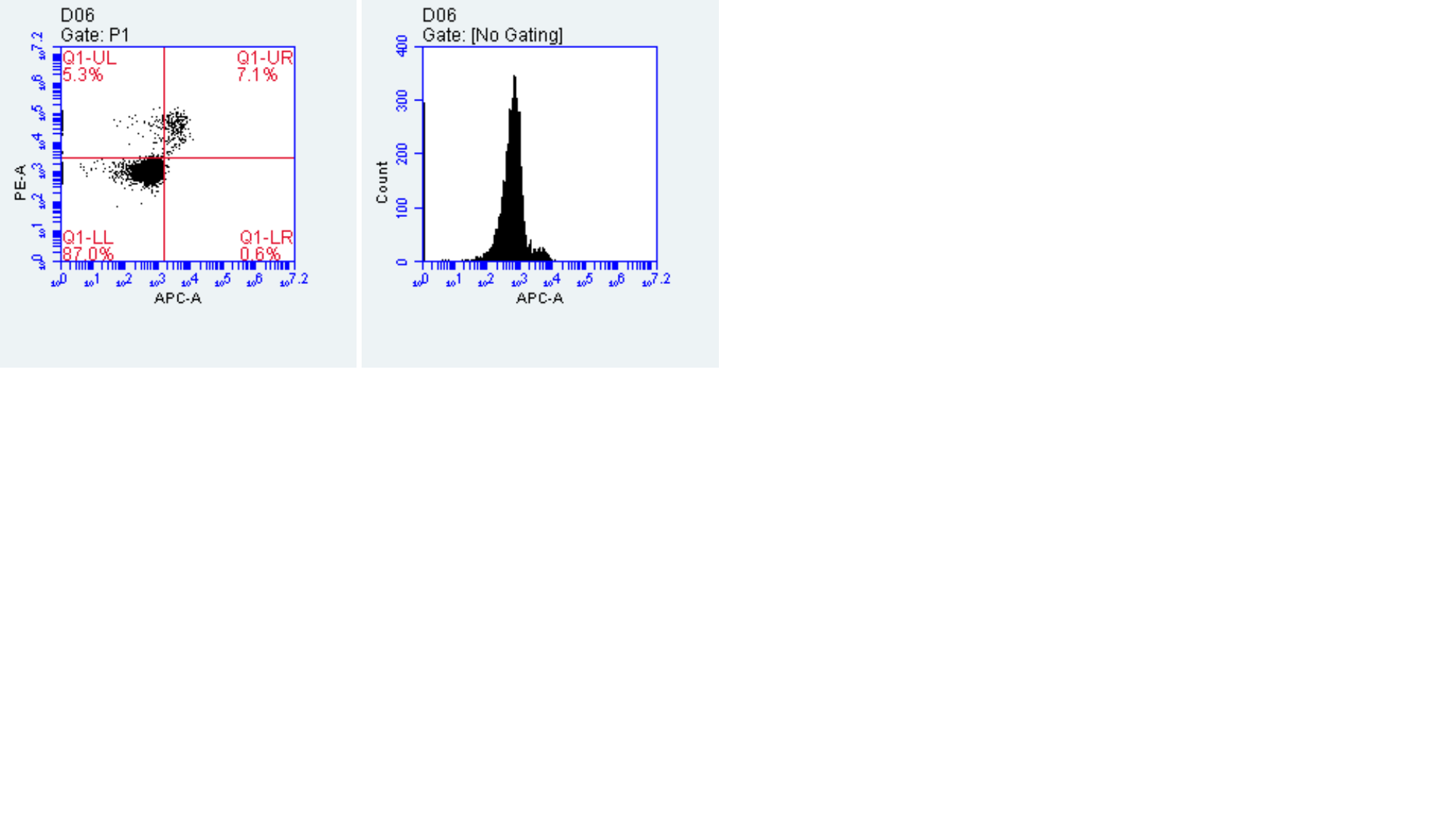

## Slide 8
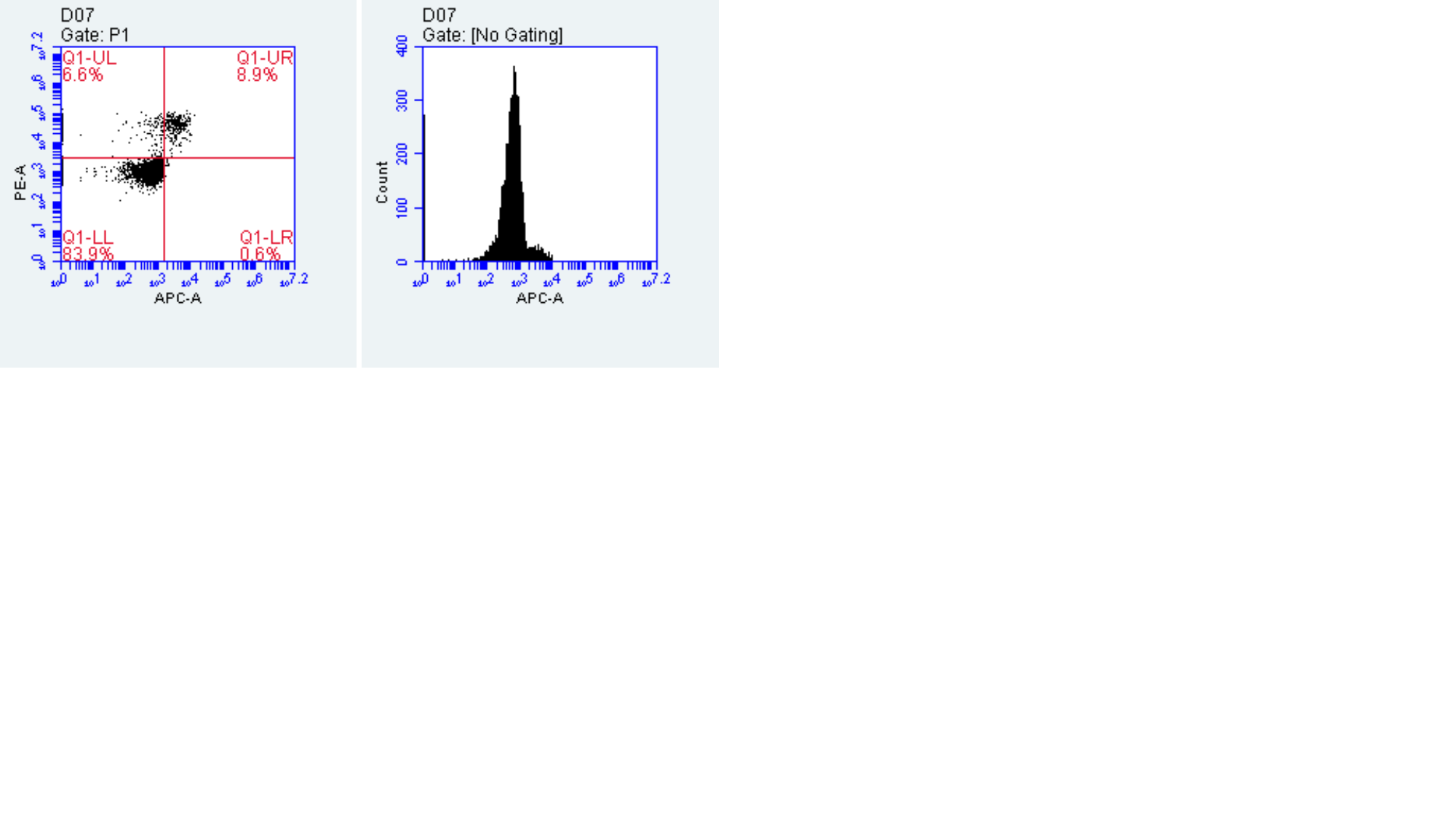

## Slide 9
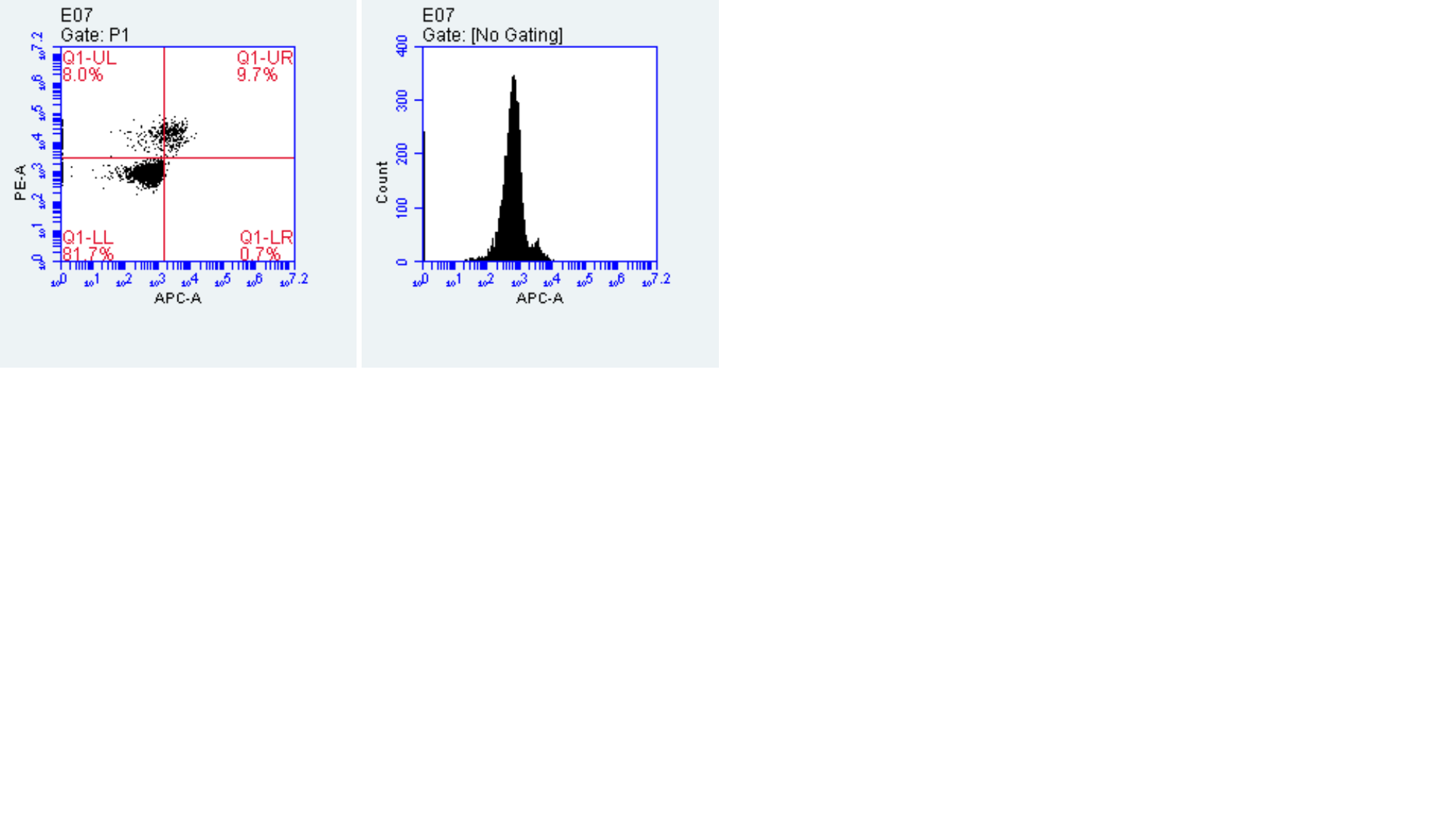

## Slide 10
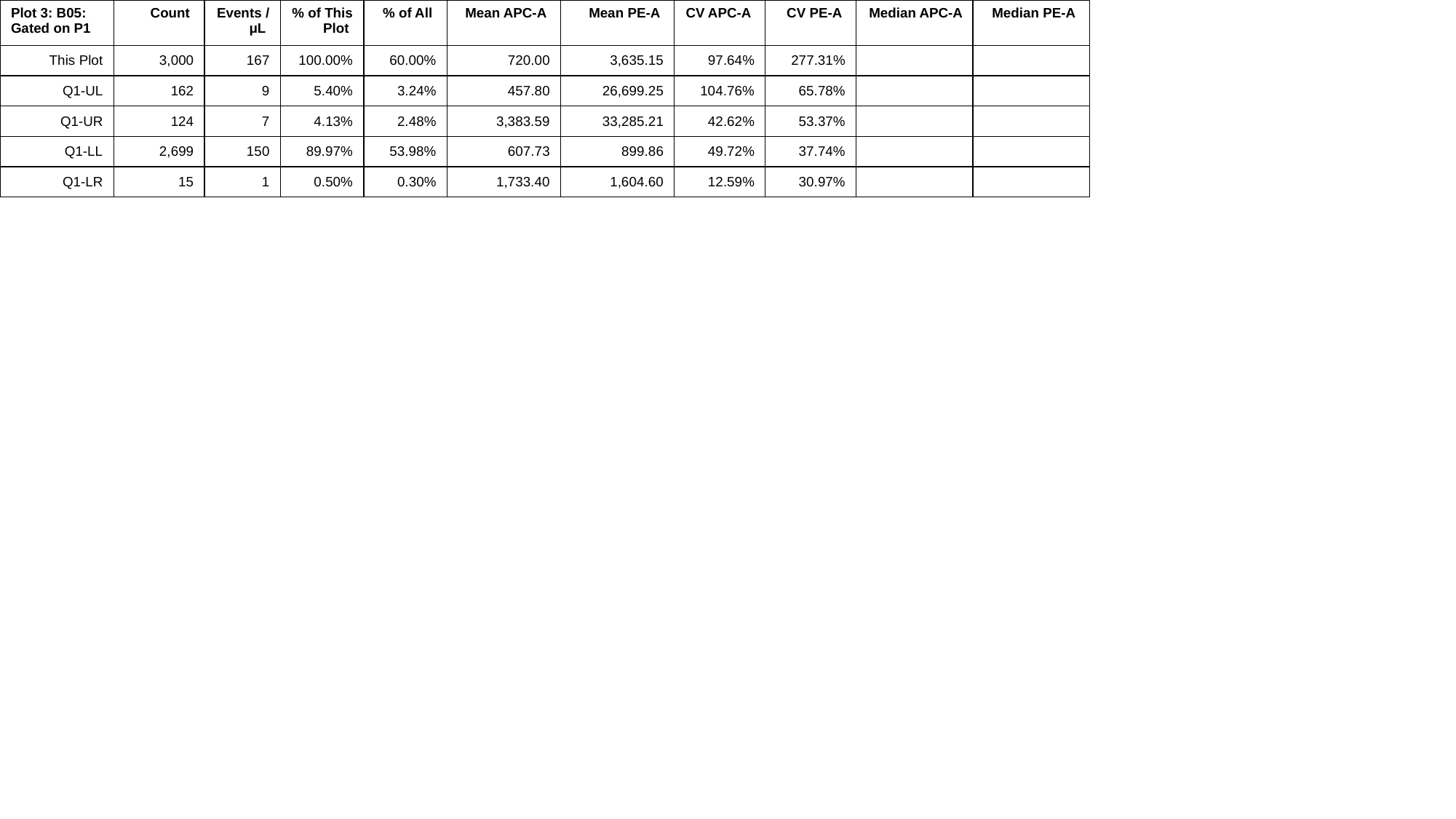

| Plot 3: B05: Gated on P1 | Count | Events / μL | % of This Plot | % of All | Mean APC-A | Mean PE-A | CV APC-A | CV PE-A | Median APC-A | Median PE-A |
| --- | --- | --- | --- | --- | --- | --- | --- | --- | --- | --- |
| This Plot | 3,000 | 167 | 100.00% | 60.00% | 720.00 | 3,635.15 | 97.64% | 277.31% | | |
| Q1-UL | 162 | 9 | 5.40% | 3.24% | 457.80 | 26,699.25 | 104.76% | 65.78% | | |
| Q1-UR | 124 | 7 | 4.13% | 2.48% | 3,383.59 | 33,285.21 | 42.62% | 53.37% | | |
| Q1-LL | 2,699 | 150 | 89.97% | 53.98% | 607.73 | 899.86 | 49.72% | 37.74% | | |
| Q1-LR | 15 | 1 | 0.50% | 0.30% | 1,733.40 | 1,604.60 | 12.59% | 30.97% | | |

## Slide 11
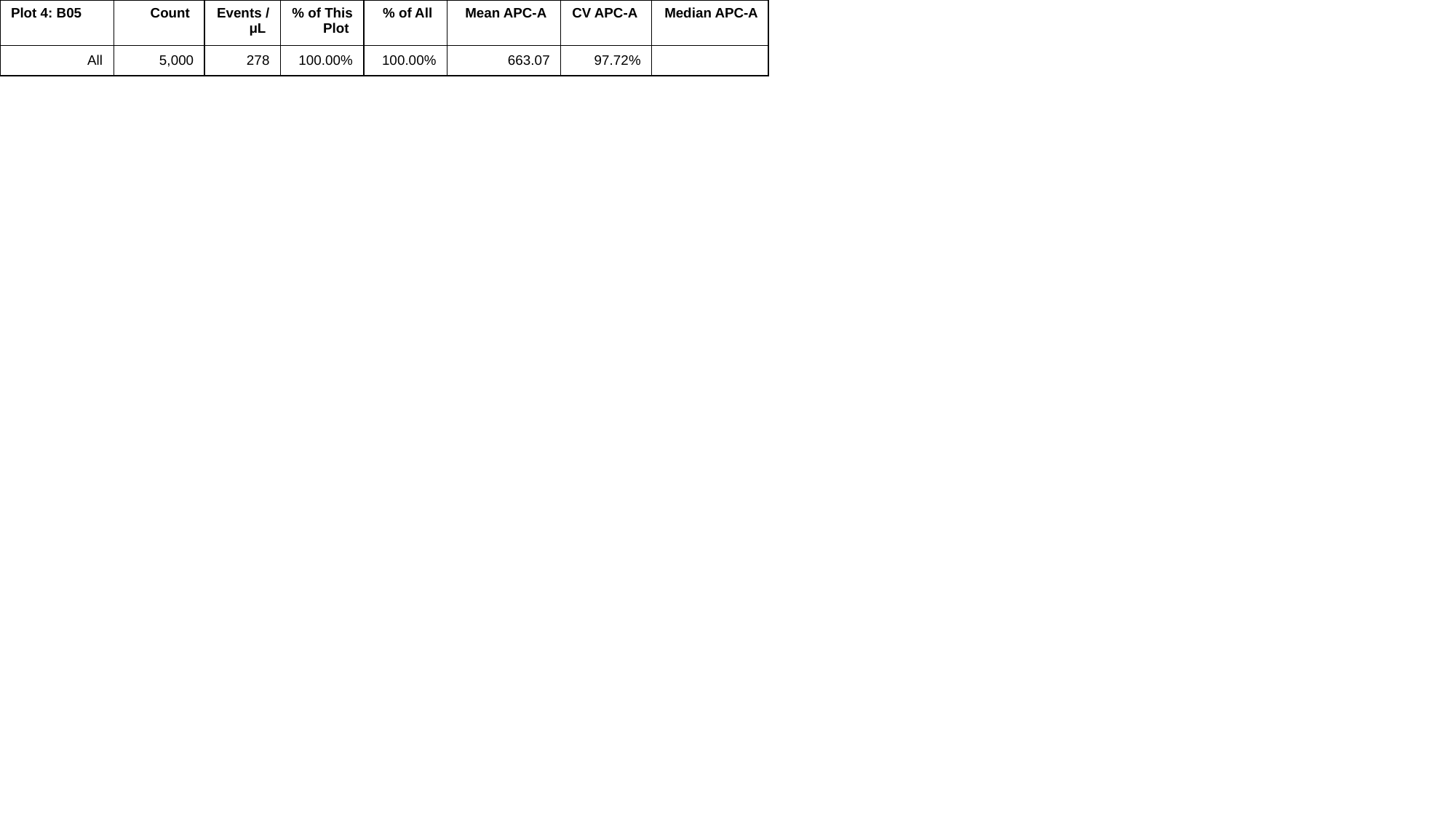

| Plot 4: B05 | Count | Events / μL | % of This Plot | % of All | Mean APC-A | CV APC-A | Median APC-A |
| --- | --- | --- | --- | --- | --- | --- | --- |
| All | 5,000 | 278 | 100.00% | 100.00% | 663.07 | 97.72% | |

## Slide 12
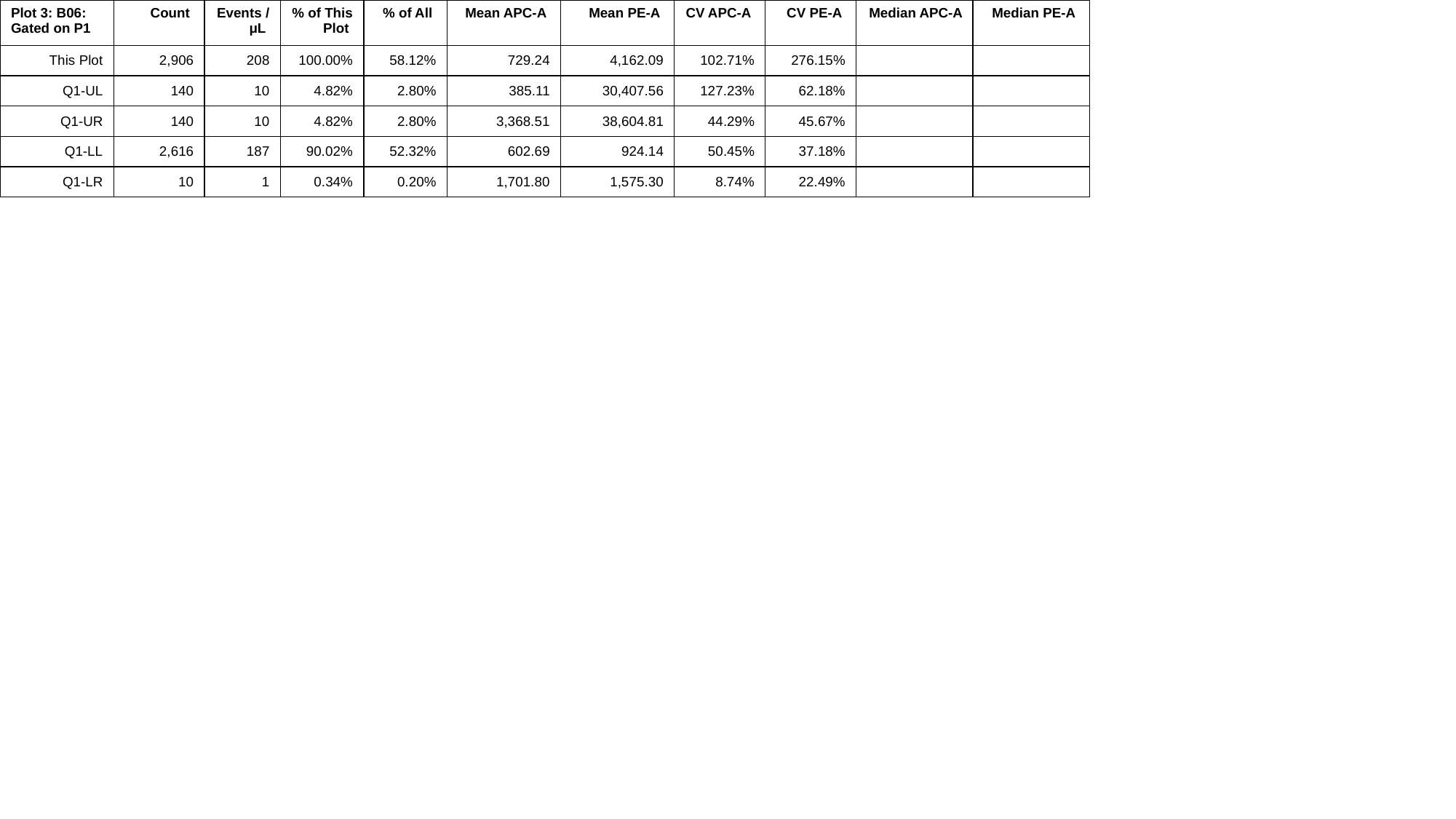

| Plot 3: B06: Gated on P1 | Count | Events / μL | % of This Plot | % of All | Mean APC-A | Mean PE-A | CV APC-A | CV PE-A | Median APC-A | Median PE-A |
| --- | --- | --- | --- | --- | --- | --- | --- | --- | --- | --- |
| This Plot | 2,906 | 208 | 100.00% | 58.12% | 729.24 | 4,162.09 | 102.71% | 276.15% | | |
| Q1-UL | 140 | 10 | 4.82% | 2.80% | 385.11 | 30,407.56 | 127.23% | 62.18% | | |
| Q1-UR | 140 | 10 | 4.82% | 2.80% | 3,368.51 | 38,604.81 | 44.29% | 45.67% | | |
| Q1-LL | 2,616 | 187 | 90.02% | 52.32% | 602.69 | 924.14 | 50.45% | 37.18% | | |
| Q1-LR | 10 | 1 | 0.34% | 0.20% | 1,701.80 | 1,575.30 | 8.74% | 22.49% | | |

## Slide 13
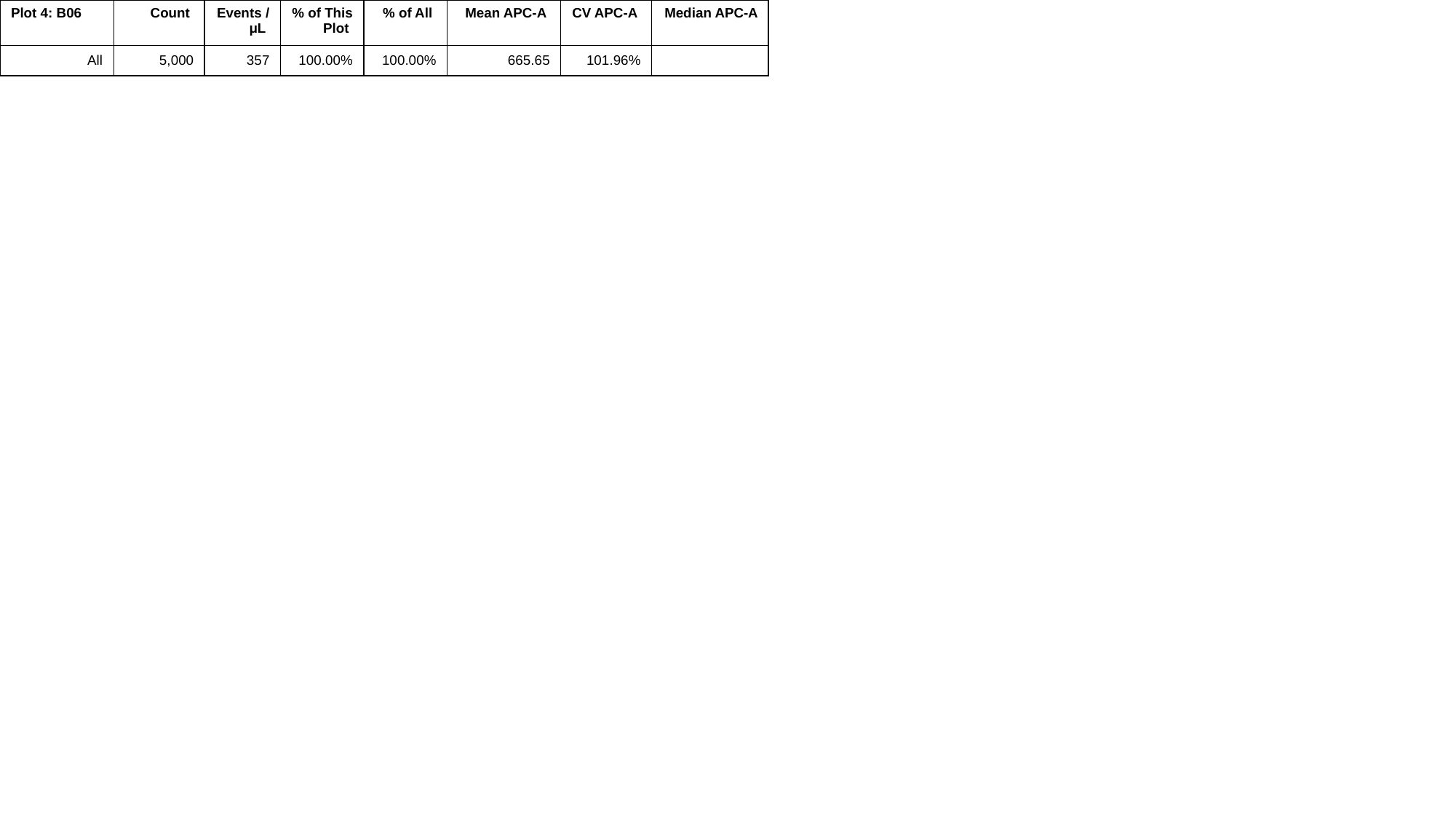

| Plot 4: B06 | Count | Events / μL | % of This Plot | % of All | Mean APC-A | CV APC-A | Median APC-A |
| --- | --- | --- | --- | --- | --- | --- | --- |
| All | 5,000 | 357 | 100.00% | 100.00% | 665.65 | 101.96% | |

## Slide 14
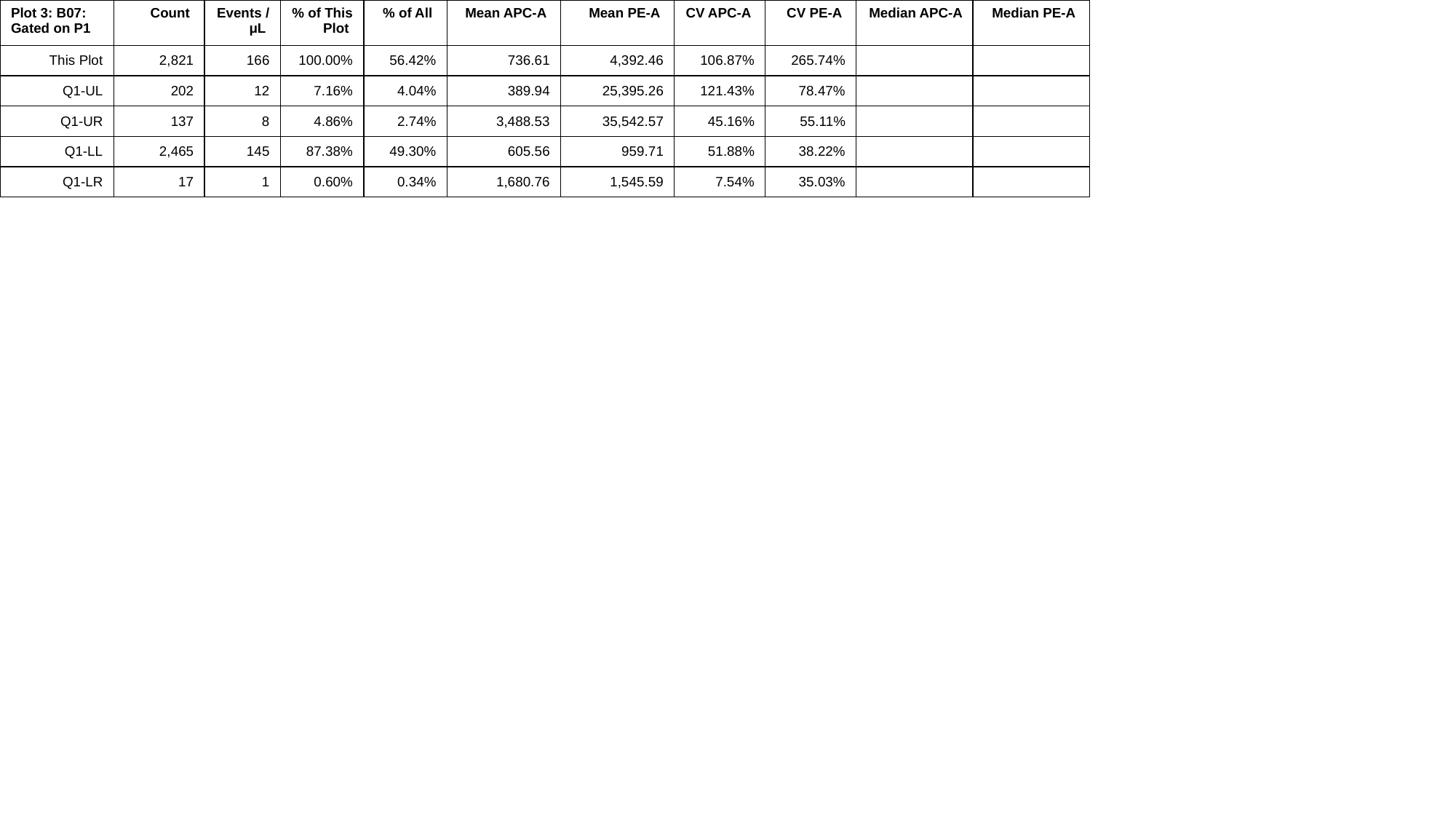

| Plot 3: B07: Gated on P1 | Count | Events / μL | % of This Plot | % of All | Mean APC-A | Mean PE-A | CV APC-A | CV PE-A | Median APC-A | Median PE-A |
| --- | --- | --- | --- | --- | --- | --- | --- | --- | --- | --- |
| This Plot | 2,821 | 166 | 100.00% | 56.42% | 736.61 | 4,392.46 | 106.87% | 265.74% | | |
| Q1-UL | 202 | 12 | 7.16% | 4.04% | 389.94 | 25,395.26 | 121.43% | 78.47% | | |
| Q1-UR | 137 | 8 | 4.86% | 2.74% | 3,488.53 | 35,542.57 | 45.16% | 55.11% | | |
| Q1-LL | 2,465 | 145 | 87.38% | 49.30% | 605.56 | 959.71 | 51.88% | 38.22% | | |
| Q1-LR | 17 | 1 | 0.60% | 0.34% | 1,680.76 | 1,545.59 | 7.54% | 35.03% | | |

## Slide 15
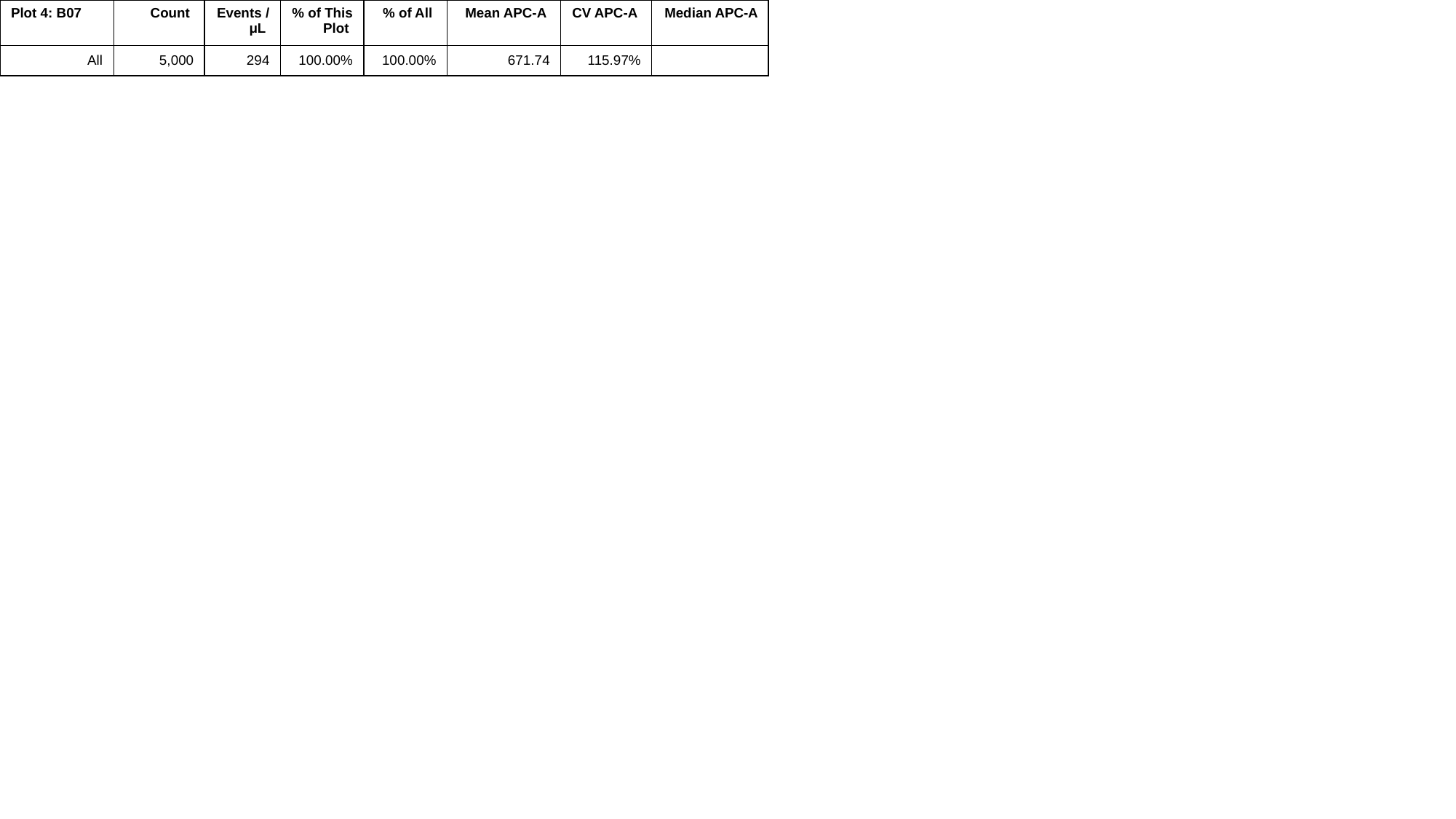

| Plot 4: B07 | Count | Events / μL | % of This Plot | % of All | Mean APC-A | CV APC-A | Median APC-A |
| --- | --- | --- | --- | --- | --- | --- | --- |
| All | 5,000 | 294 | 100.00% | 100.00% | 671.74 | 115.97% | |

## Slide 16
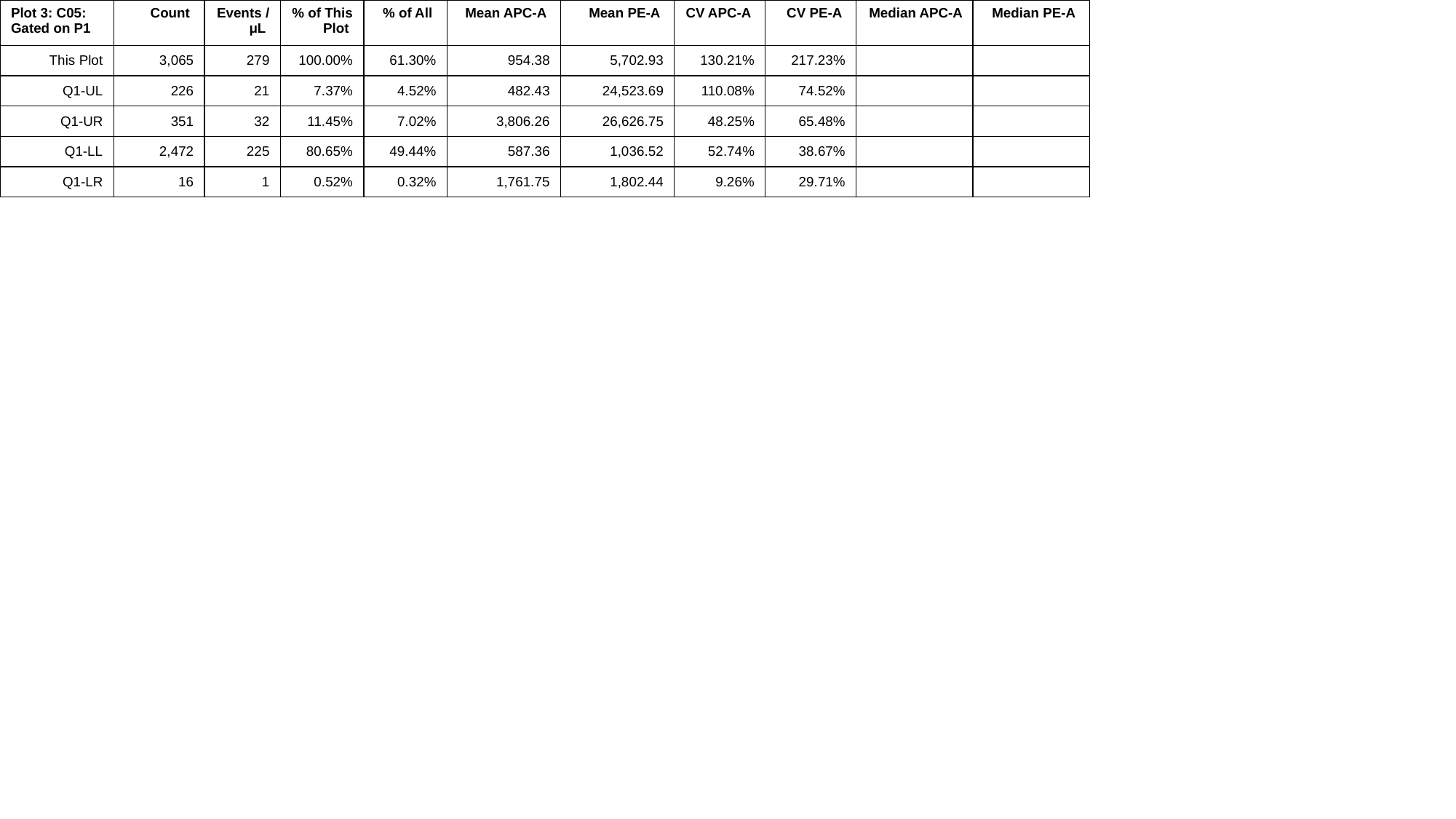

| Plot 3: C05: Gated on P1 | Count | Events / μL | % of This Plot | % of All | Mean APC-A | Mean PE-A | CV APC-A | CV PE-A | Median APC-A | Median PE-A |
| --- | --- | --- | --- | --- | --- | --- | --- | --- | --- | --- |
| This Plot | 3,065 | 279 | 100.00% | 61.30% | 954.38 | 5,702.93 | 130.21% | 217.23% | | |
| Q1-UL | 226 | 21 | 7.37% | 4.52% | 482.43 | 24,523.69 | 110.08% | 74.52% | | |
| Q1-UR | 351 | 32 | 11.45% | 7.02% | 3,806.26 | 26,626.75 | 48.25% | 65.48% | | |
| Q1-LL | 2,472 | 225 | 80.65% | 49.44% | 587.36 | 1,036.52 | 52.74% | 38.67% | | |
| Q1-LR | 16 | 1 | 0.52% | 0.32% | 1,761.75 | 1,802.44 | 9.26% | 29.71% | | |

## Slide 17
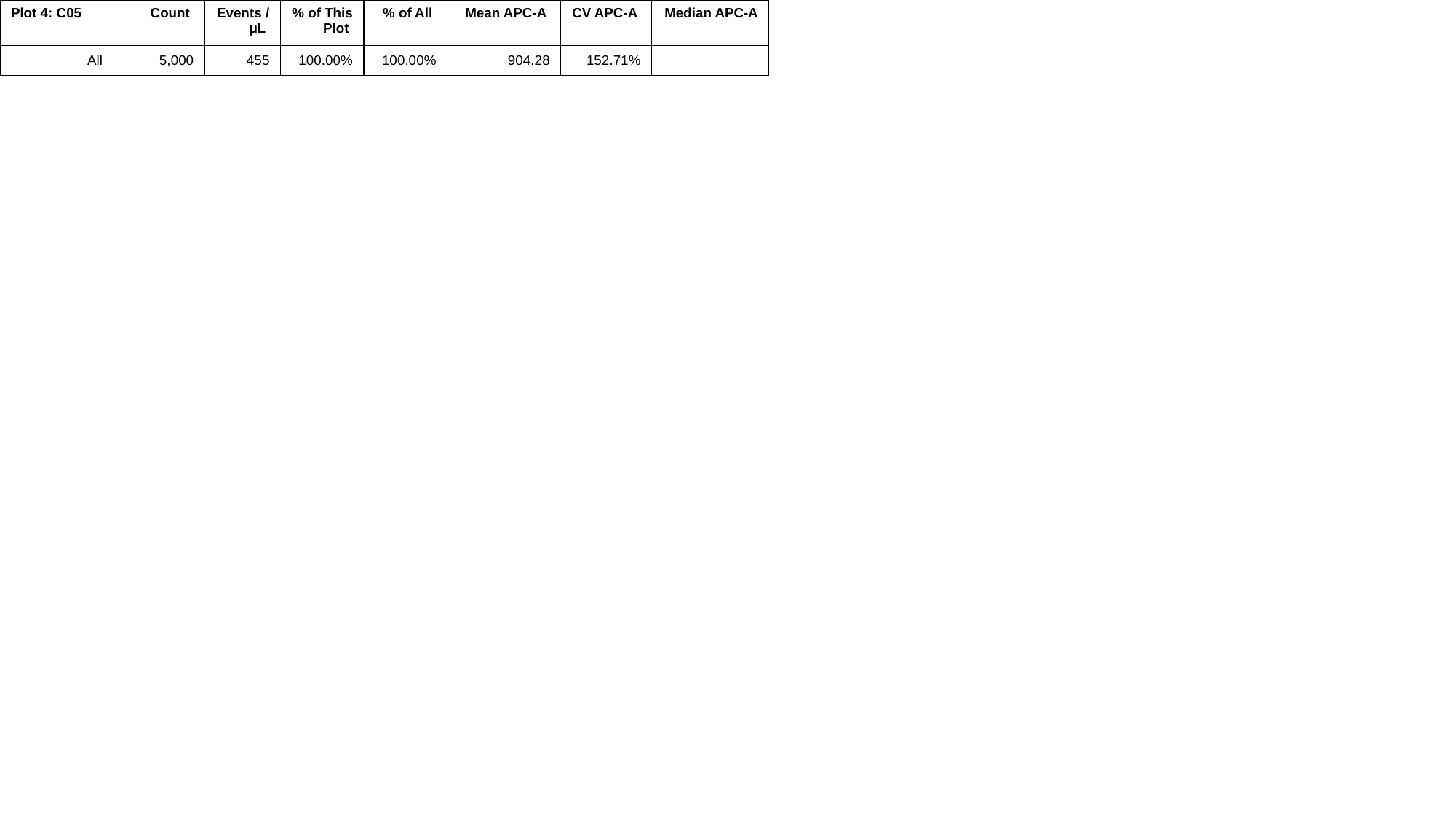

| Plot 4: C05 | Count | Events / μL | % of This Plot | % of All | Mean APC-A | CV APC-A | Median APC-A |
| --- | --- | --- | --- | --- | --- | --- | --- |
| All | 5,000 | 455 | 100.00% | 100.00% | 904.28 | 152.71% | |

## Slide 18
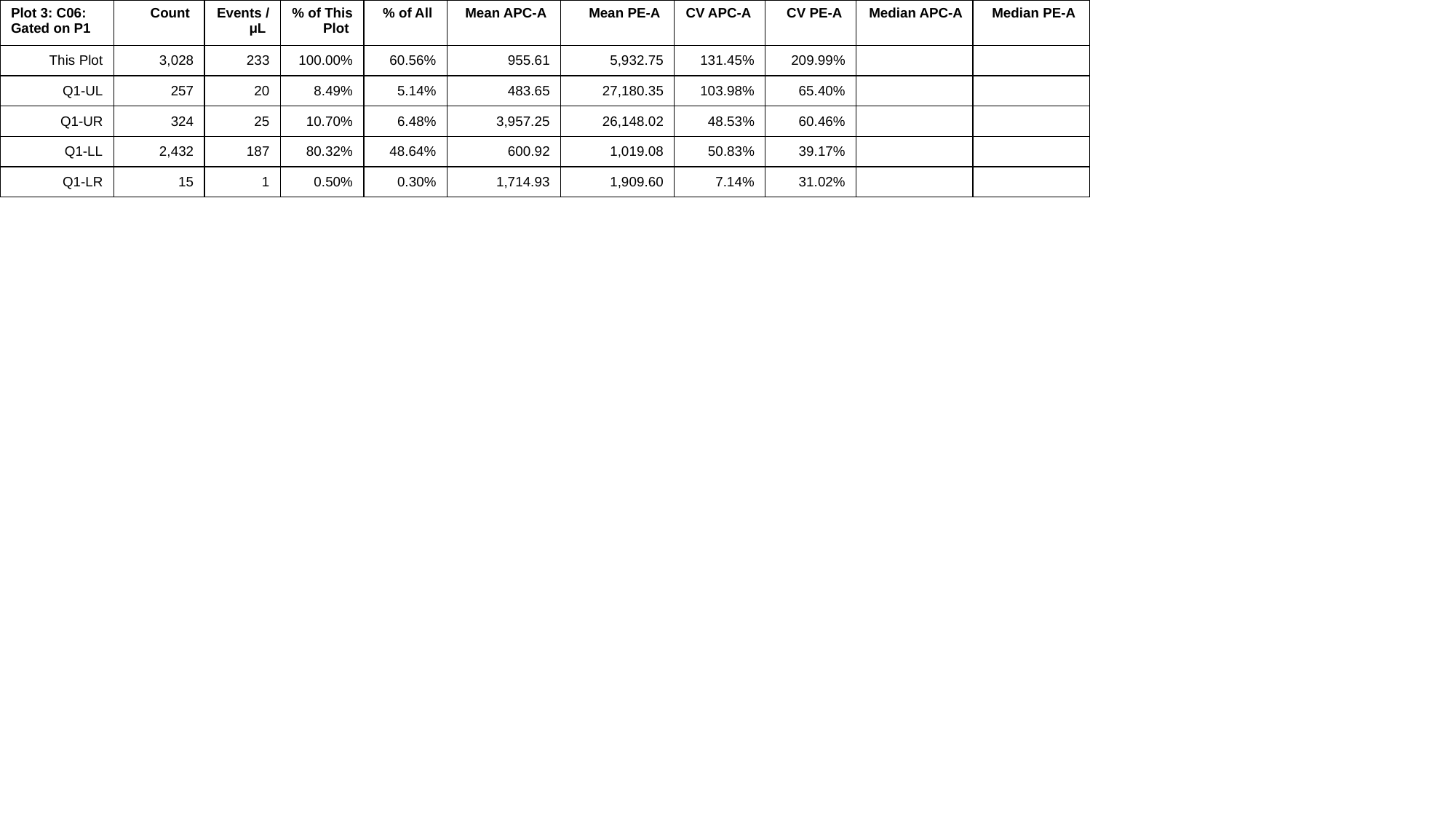

| Plot 3: C06: Gated on P1 | Count | Events / μL | % of This Plot | % of All | Mean APC-A | Mean PE-A | CV APC-A | CV PE-A | Median APC-A | Median PE-A |
| --- | --- | --- | --- | --- | --- | --- | --- | --- | --- | --- |
| This Plot | 3,028 | 233 | 100.00% | 60.56% | 955.61 | 5,932.75 | 131.45% | 209.99% | | |
| Q1-UL | 257 | 20 | 8.49% | 5.14% | 483.65 | 27,180.35 | 103.98% | 65.40% | | |
| Q1-UR | 324 | 25 | 10.70% | 6.48% | 3,957.25 | 26,148.02 | 48.53% | 60.46% | | |
| Q1-LL | 2,432 | 187 | 80.32% | 48.64% | 600.92 | 1,019.08 | 50.83% | 39.17% | | |
| Q1-LR | 15 | 1 | 0.50% | 0.30% | 1,714.93 | 1,909.60 | 7.14% | 31.02% | | |

## Slide 19
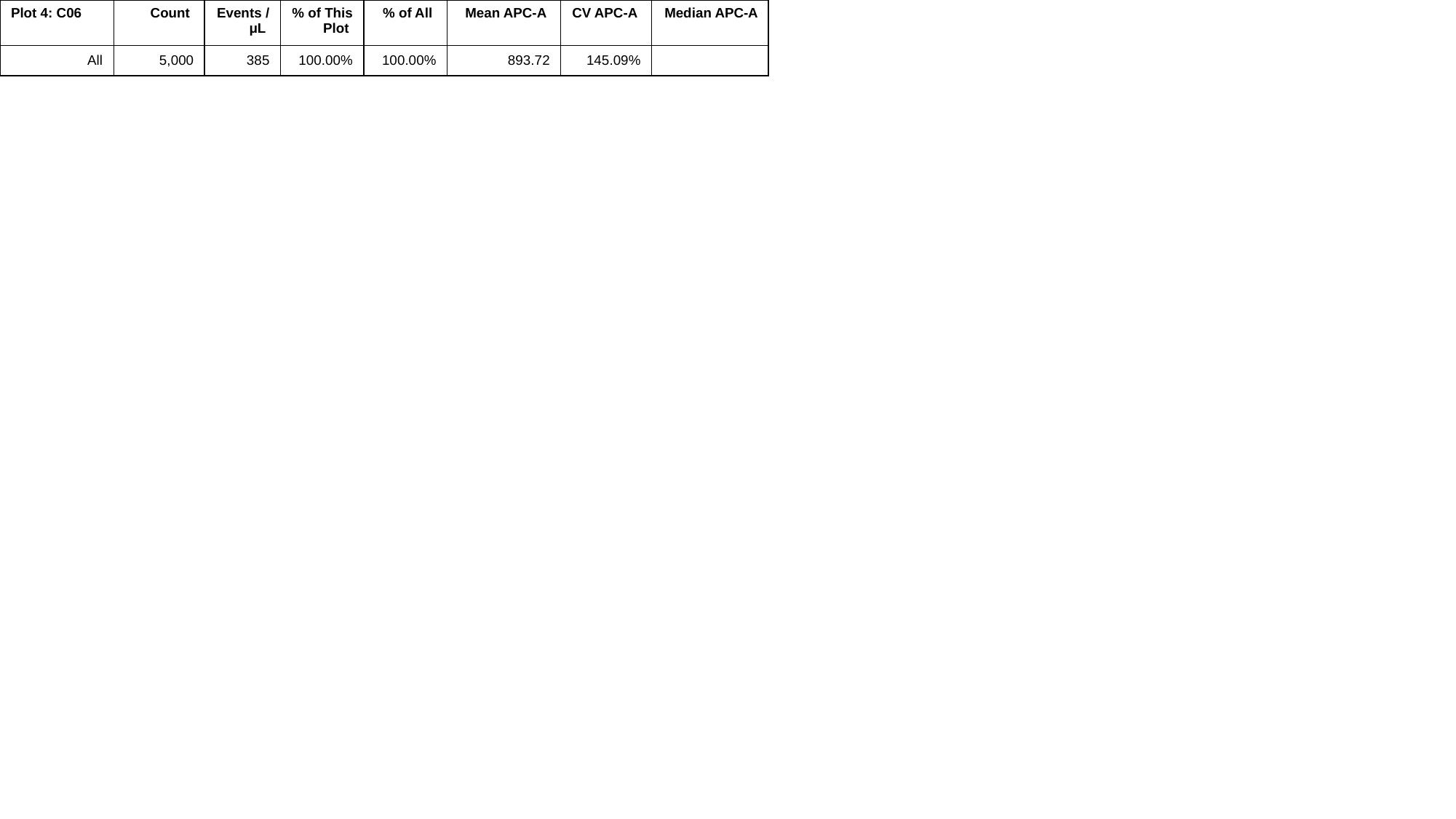

| Plot 4: C06 | Count | Events / μL | % of This Plot | % of All | Mean APC-A | CV APC-A | Median APC-A |
| --- | --- | --- | --- | --- | --- | --- | --- |
| All | 5,000 | 385 | 100.00% | 100.00% | 893.72 | 145.09% | |

## Slide 20
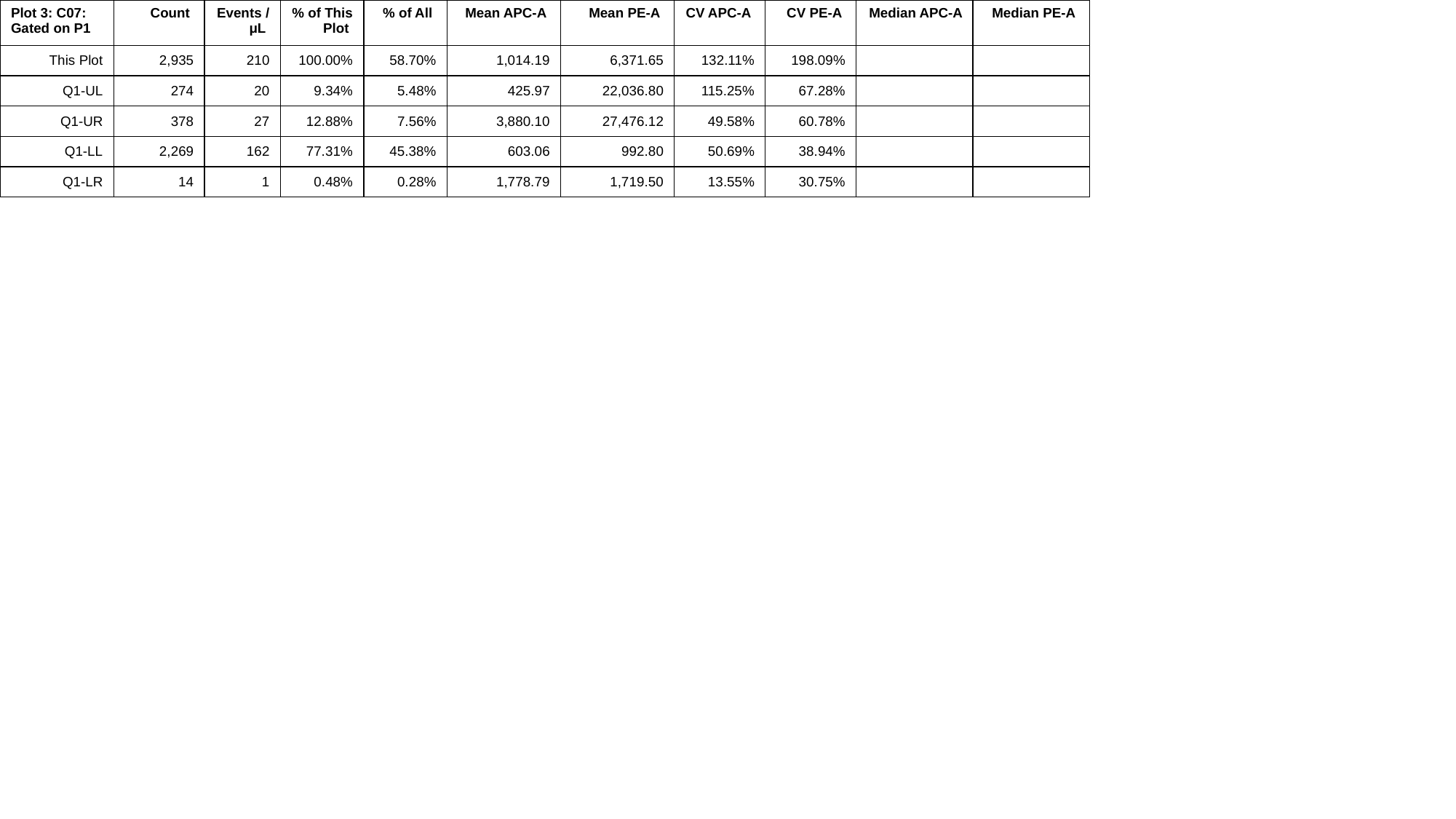

| Plot 3: C07: Gated on P1 | Count | Events / μL | % of This Plot | % of All | Mean APC-A | Mean PE-A | CV APC-A | CV PE-A | Median APC-A | Median PE-A |
| --- | --- | --- | --- | --- | --- | --- | --- | --- | --- | --- |
| This Plot | 2,935 | 210 | 100.00% | 58.70% | 1,014.19 | 6,371.65 | 132.11% | 198.09% | | |
| Q1-UL | 274 | 20 | 9.34% | 5.48% | 425.97 | 22,036.80 | 115.25% | 67.28% | | |
| Q1-UR | 378 | 27 | 12.88% | 7.56% | 3,880.10 | 27,476.12 | 49.58% | 60.78% | | |
| Q1-LL | 2,269 | 162 | 77.31% | 45.38% | 603.06 | 992.80 | 50.69% | 38.94% | | |
| Q1-LR | 14 | 1 | 0.48% | 0.28% | 1,778.79 | 1,719.50 | 13.55% | 30.75% | | |

## Slide 21
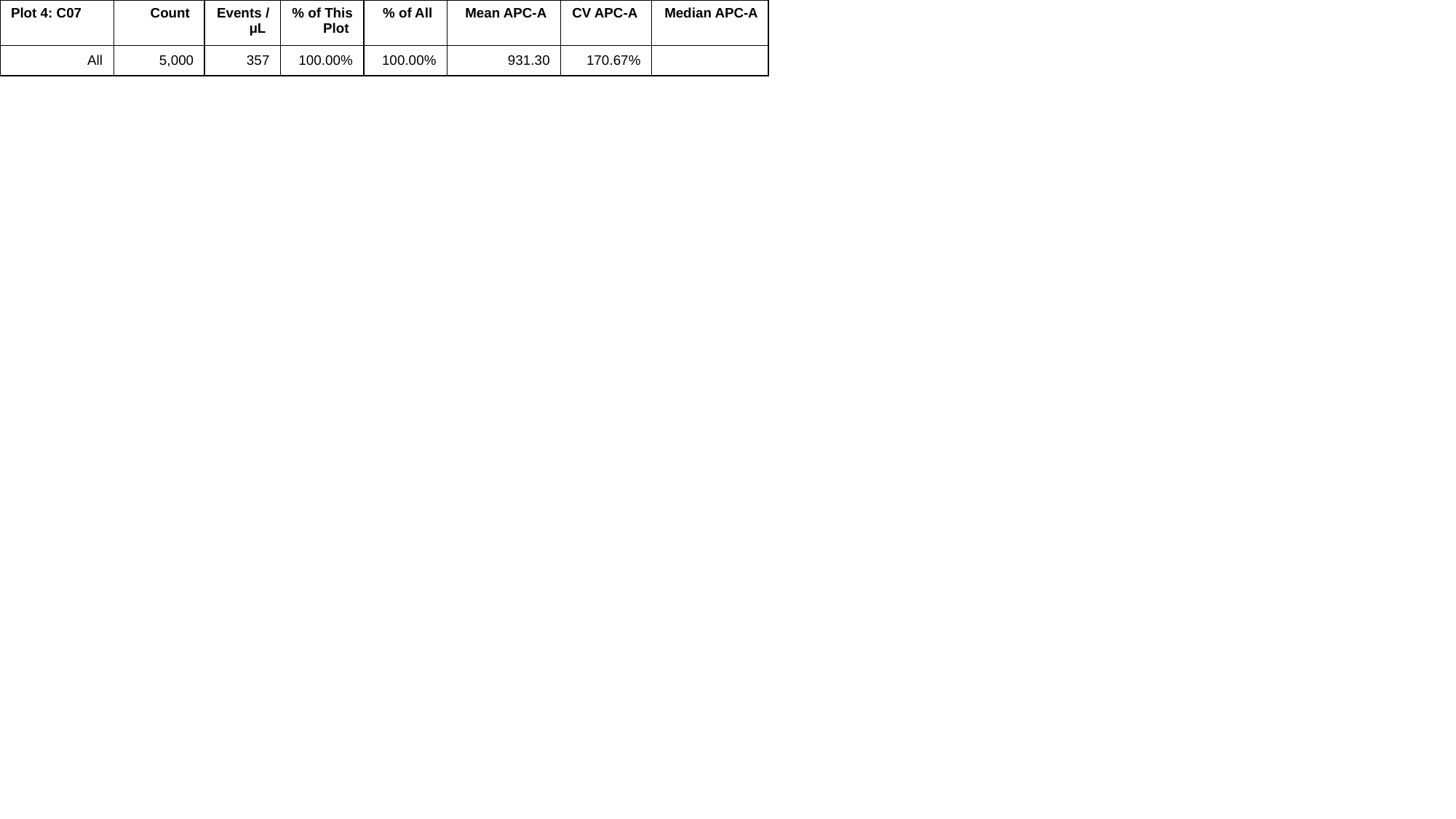

| Plot 4: C07 | Count | Events / μL | % of This Plot | % of All | Mean APC-A | CV APC-A | Median APC-A |
| --- | --- | --- | --- | --- | --- | --- | --- |
| All | 5,000 | 357 | 100.00% | 100.00% | 931.30 | 170.67% | |

## Slide 22
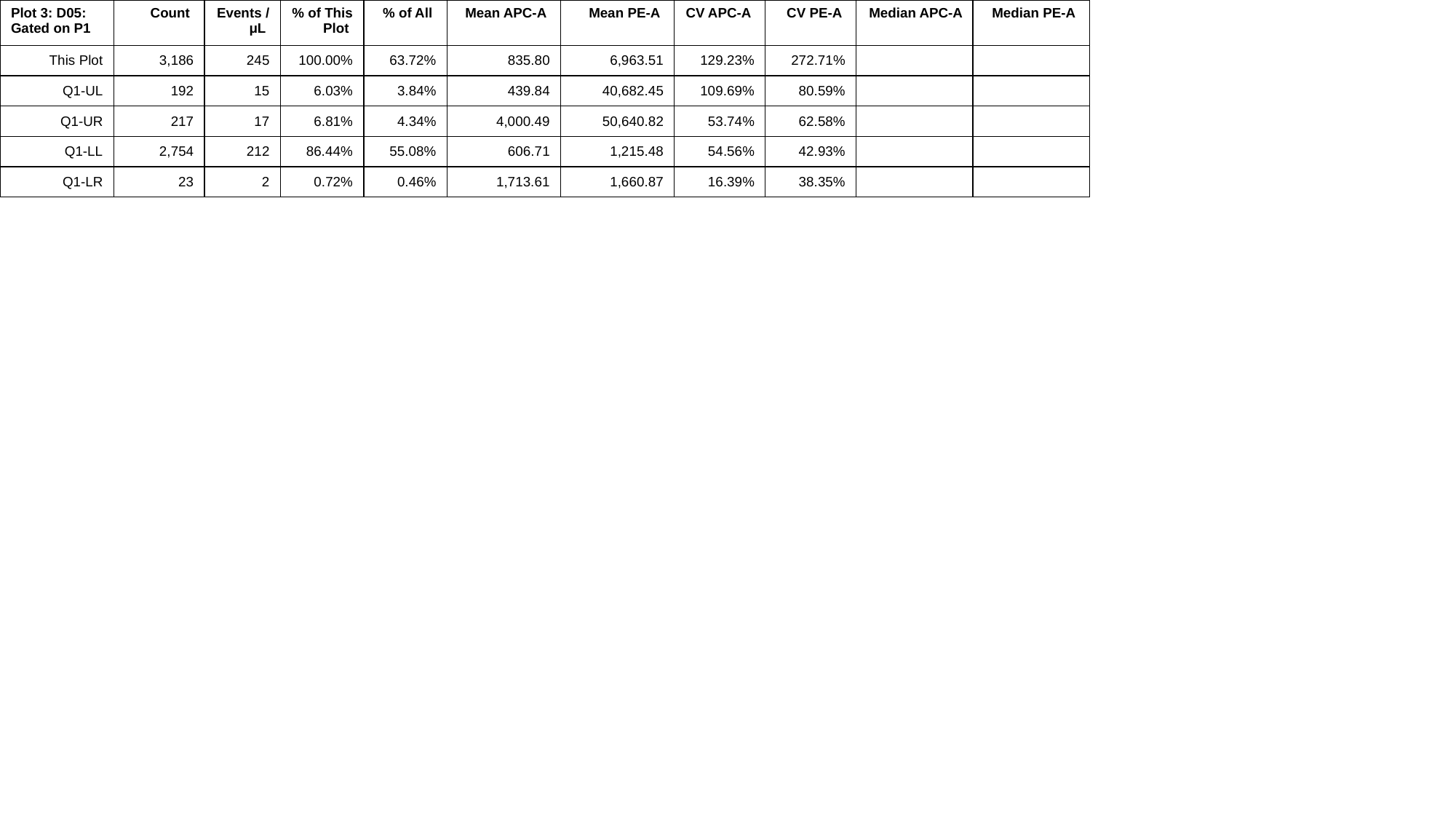

| Plot 3: D05: Gated on P1 | Count | Events / μL | % of This Plot | % of All | Mean APC-A | Mean PE-A | CV APC-A | CV PE-A | Median APC-A | Median PE-A |
| --- | --- | --- | --- | --- | --- | --- | --- | --- | --- | --- |
| This Plot | 3,186 | 245 | 100.00% | 63.72% | 835.80 | 6,963.51 | 129.23% | 272.71% | | |
| Q1-UL | 192 | 15 | 6.03% | 3.84% | 439.84 | 40,682.45 | 109.69% | 80.59% | | |
| Q1-UR | 217 | 17 | 6.81% | 4.34% | 4,000.49 | 50,640.82 | 53.74% | 62.58% | | |
| Q1-LL | 2,754 | 212 | 86.44% | 55.08% | 606.71 | 1,215.48 | 54.56% | 42.93% | | |
| Q1-LR | 23 | 2 | 0.72% | 0.46% | 1,713.61 | 1,660.87 | 16.39% | 38.35% | | |

## Slide 23
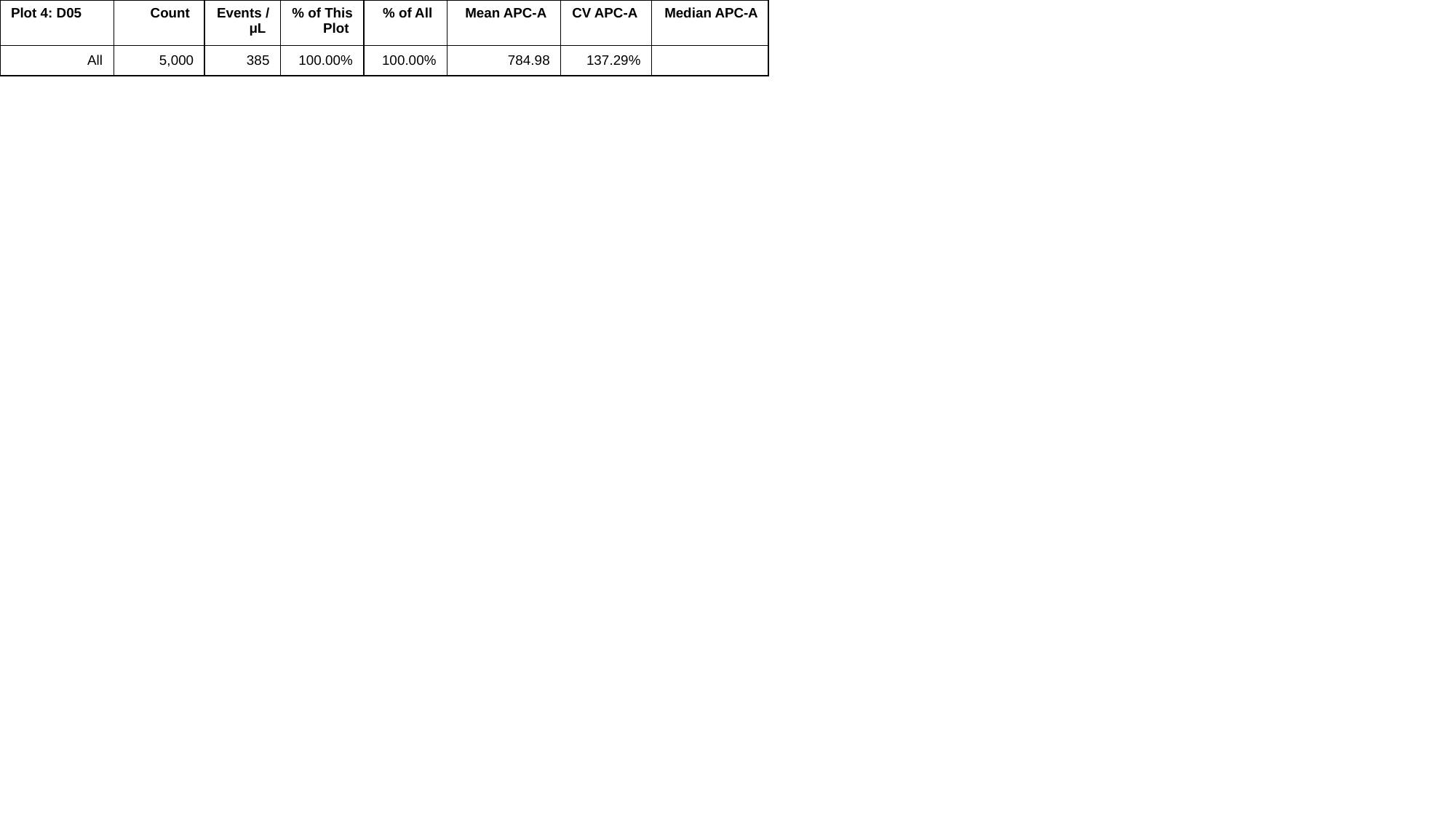

| Plot 4: D05 | Count | Events / μL | % of This Plot | % of All | Mean APC-A | CV APC-A | Median APC-A |
| --- | --- | --- | --- | --- | --- | --- | --- |
| All | 5,000 | 385 | 100.00% | 100.00% | 784.98 | 137.29% | |

## Slide 24
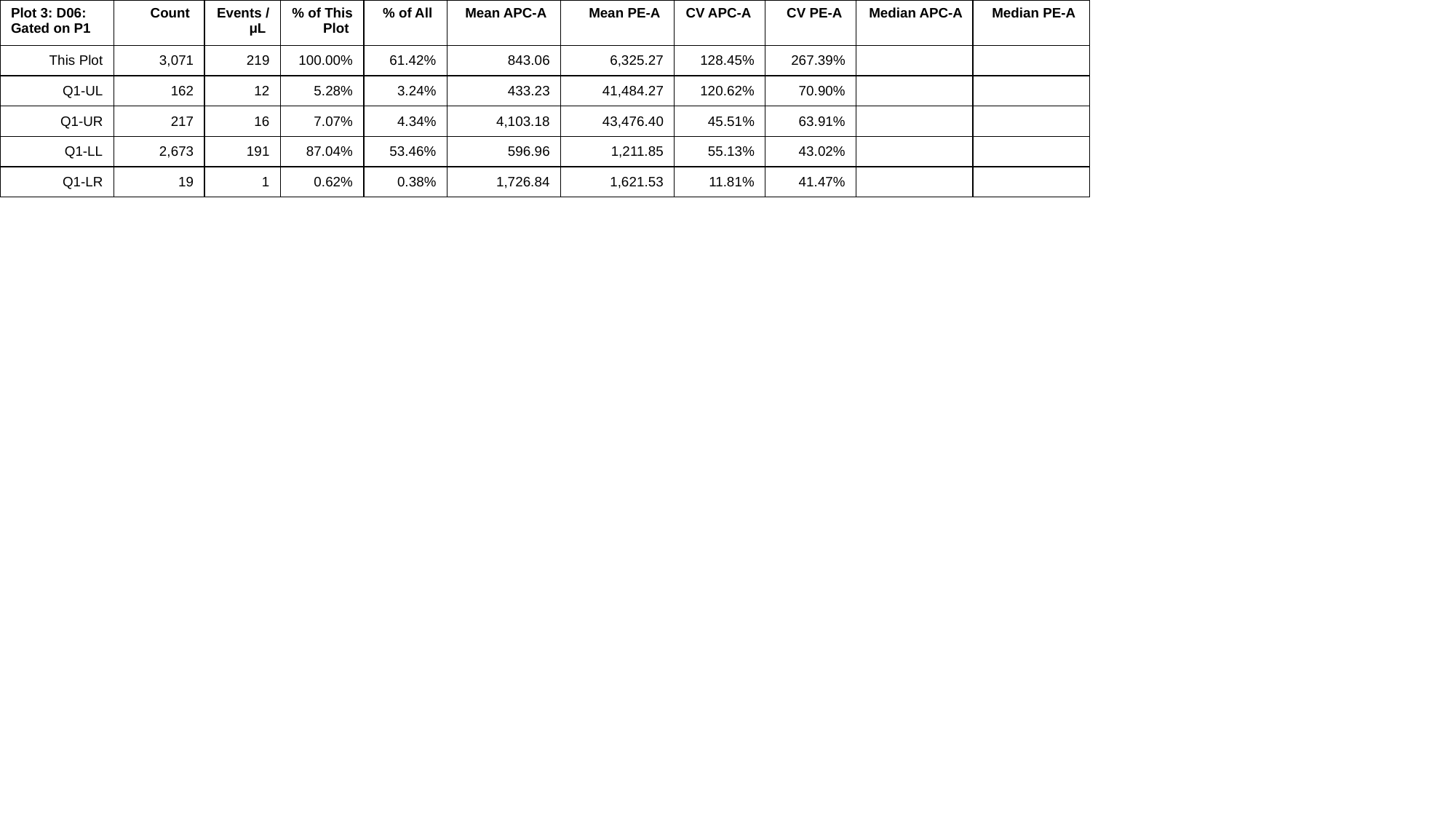

| Plot 3: D06: Gated on P1 | Count | Events / μL | % of This Plot | % of All | Mean APC-A | Mean PE-A | CV APC-A | CV PE-A | Median APC-A | Median PE-A |
| --- | --- | --- | --- | --- | --- | --- | --- | --- | --- | --- |
| This Plot | 3,071 | 219 | 100.00% | 61.42% | 843.06 | 6,325.27 | 128.45% | 267.39% | | |
| Q1-UL | 162 | 12 | 5.28% | 3.24% | 433.23 | 41,484.27 | 120.62% | 70.90% | | |
| Q1-UR | 217 | 16 | 7.07% | 4.34% | 4,103.18 | 43,476.40 | 45.51% | 63.91% | | |
| Q1-LL | 2,673 | 191 | 87.04% | 53.46% | 596.96 | 1,211.85 | 55.13% | 43.02% | | |
| Q1-LR | 19 | 1 | 0.62% | 0.38% | 1,726.84 | 1,621.53 | 11.81% | 41.47% | | |

## Slide 25
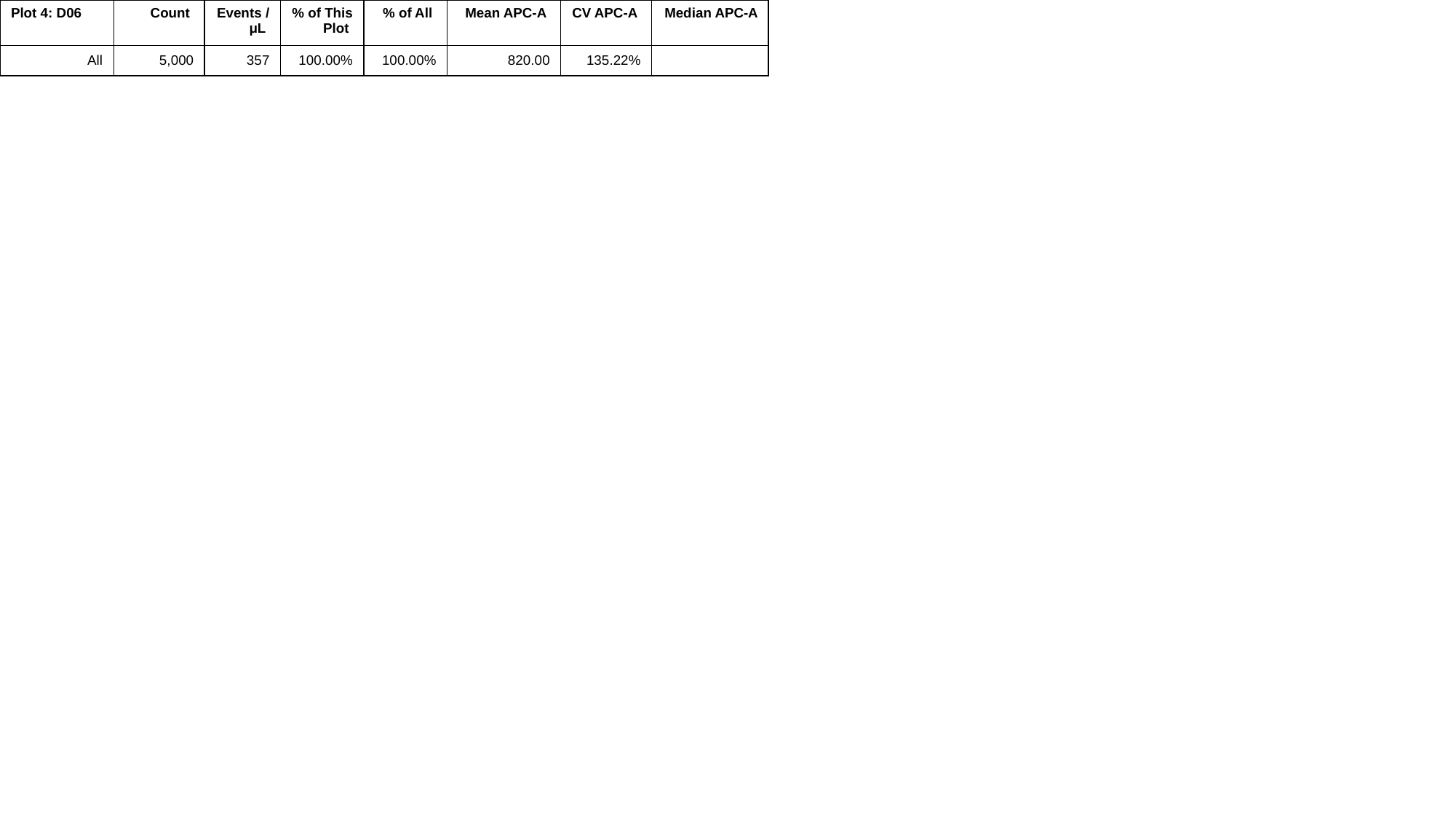

| Plot 4: D06 | Count | Events / μL | % of This Plot | % of All | Mean APC-A | CV APC-A | Median APC-A |
| --- | --- | --- | --- | --- | --- | --- | --- |
| All | 5,000 | 357 | 100.00% | 100.00% | 820.00 | 135.22% | |

## Slide 26
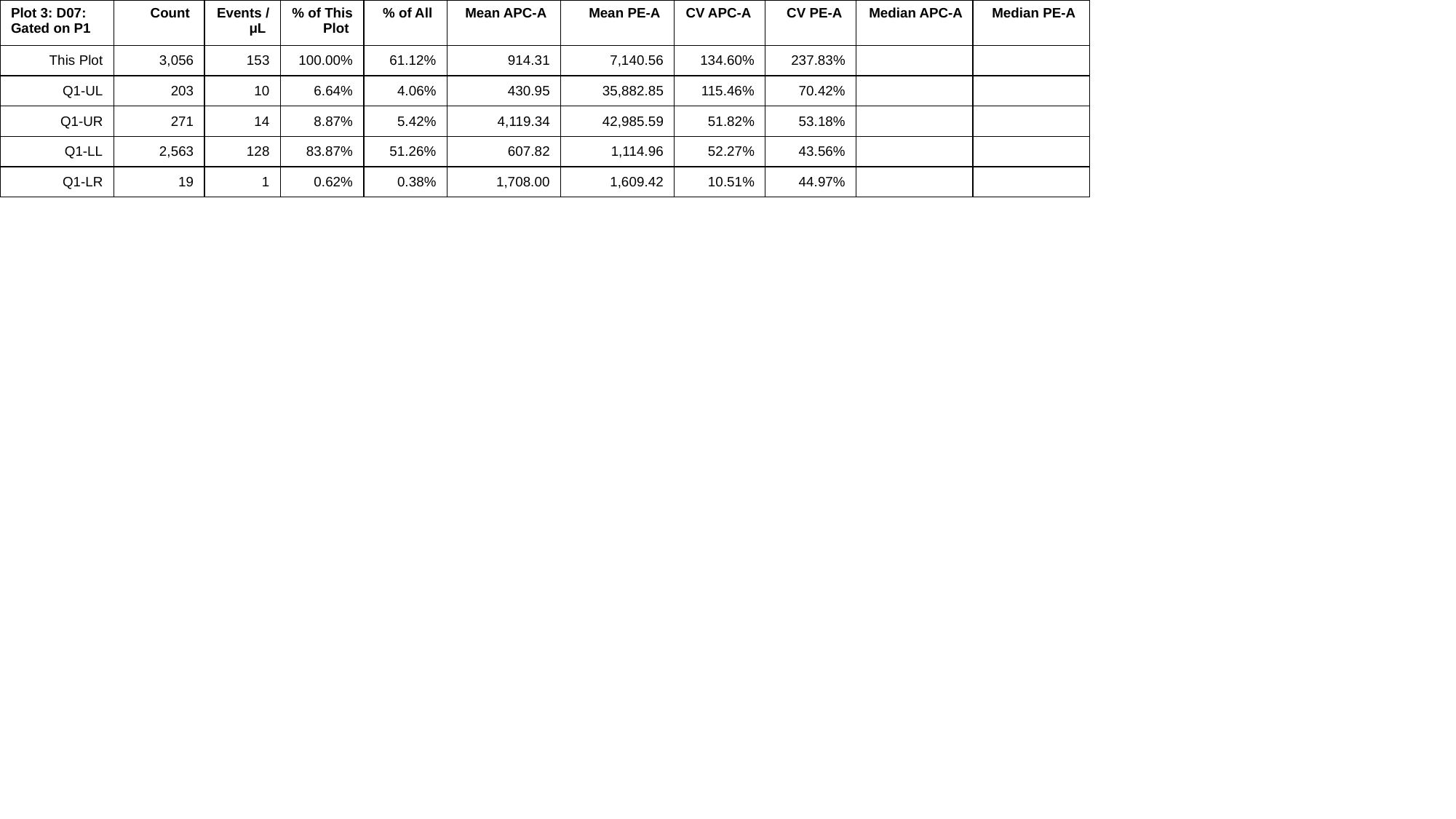

| Plot 3: D07: Gated on P1 | Count | Events / μL | % of This Plot | % of All | Mean APC-A | Mean PE-A | CV APC-A | CV PE-A | Median APC-A | Median PE-A |
| --- | --- | --- | --- | --- | --- | --- | --- | --- | --- | --- |
| This Plot | 3,056 | 153 | 100.00% | 61.12% | 914.31 | 7,140.56 | 134.60% | 237.83% | | |
| Q1-UL | 203 | 10 | 6.64% | 4.06% | 430.95 | 35,882.85 | 115.46% | 70.42% | | |
| Q1-UR | 271 | 14 | 8.87% | 5.42% | 4,119.34 | 42,985.59 | 51.82% | 53.18% | | |
| Q1-LL | 2,563 | 128 | 83.87% | 51.26% | 607.82 | 1,114.96 | 52.27% | 43.56% | | |
| Q1-LR | 19 | 1 | 0.62% | 0.38% | 1,708.00 | 1,609.42 | 10.51% | 44.97% | | |

## Slide 27
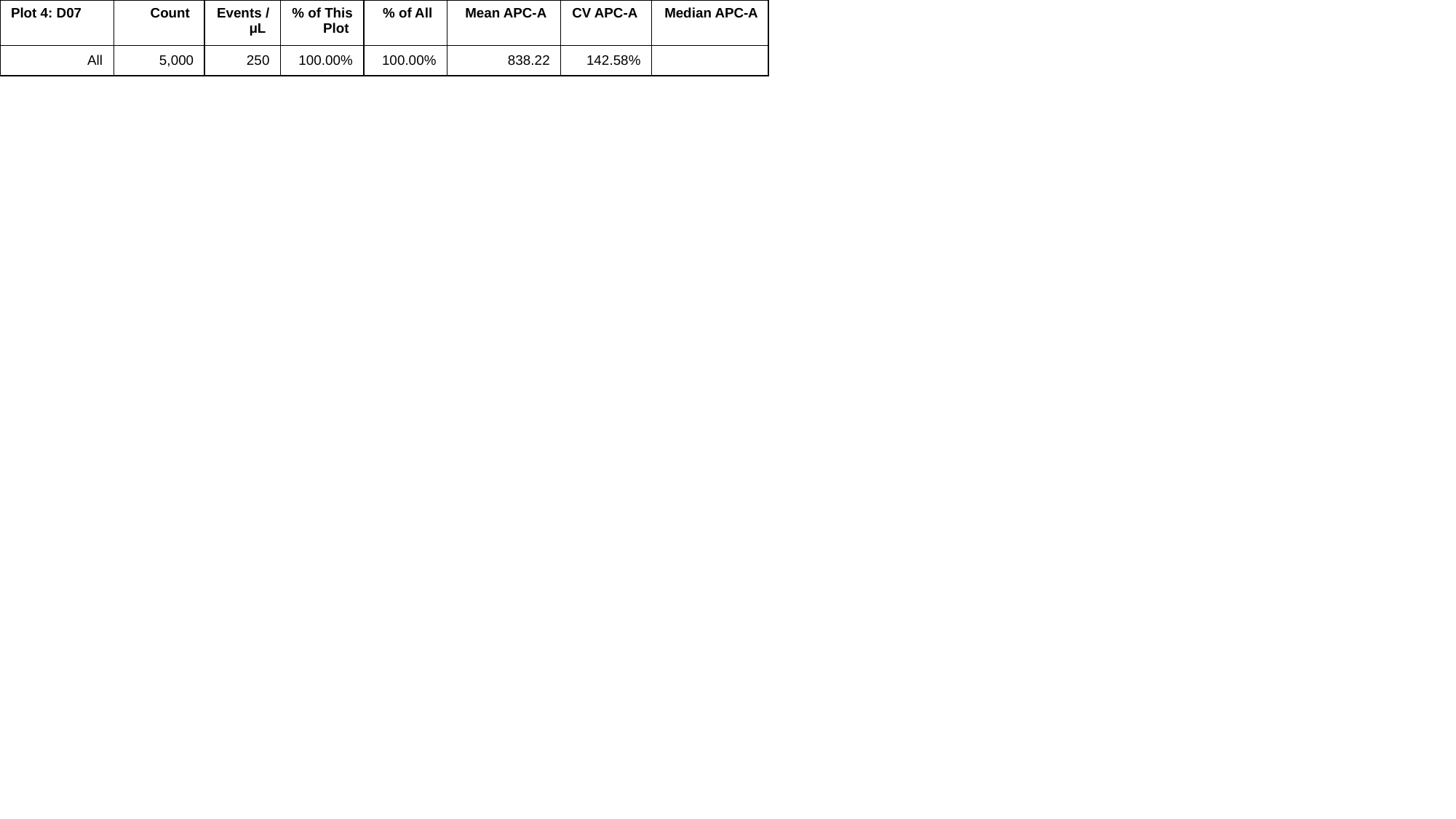

| Plot 4: D07 | Count | Events / μL | % of This Plot | % of All | Mean APC-A | CV APC-A | Median APC-A |
| --- | --- | --- | --- | --- | --- | --- | --- |
| All | 5,000 | 250 | 100.00% | 100.00% | 838.22 | 142.58% | |

## Slide 28
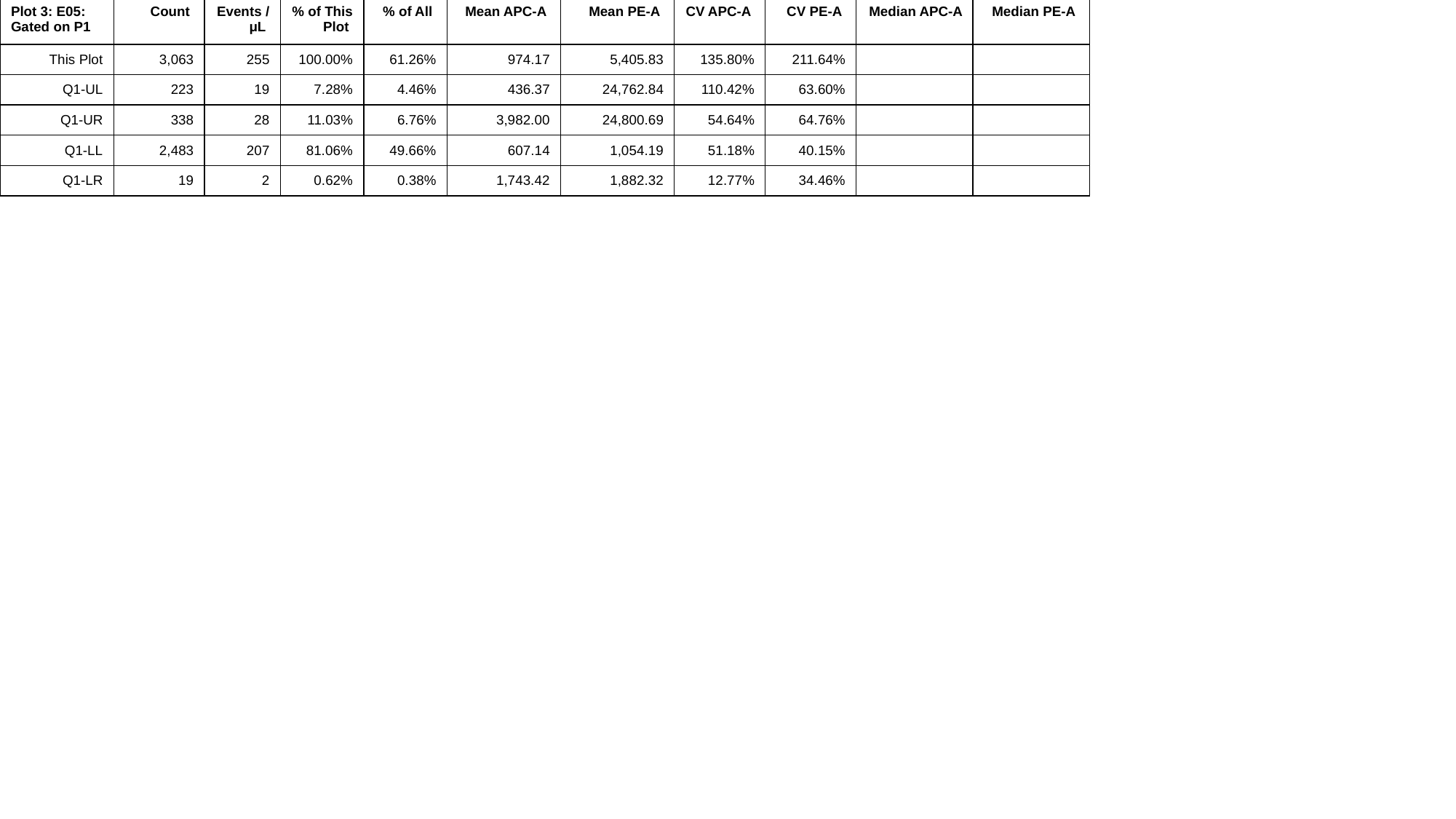

| Plot 3: E05: Gated on P1 | Count | Events / μL | % of This Plot | % of All | Mean APC-A | Mean PE-A | CV APC-A | CV PE-A | Median APC-A | Median PE-A |
| --- | --- | --- | --- | --- | --- | --- | --- | --- | --- | --- |
| This Plot | 3,063 | 255 | 100.00% | 61.26% | 974.17 | 5,405.83 | 135.80% | 211.64% | | |
| Q1-UL | 223 | 19 | 7.28% | 4.46% | 436.37 | 24,762.84 | 110.42% | 63.60% | | |
| Q1-UR | 338 | 28 | 11.03% | 6.76% | 3,982.00 | 24,800.69 | 54.64% | 64.76% | | |
| Q1-LL | 2,483 | 207 | 81.06% | 49.66% | 607.14 | 1,054.19 | 51.18% | 40.15% | | |
| Q1-LR | 19 | 2 | 0.62% | 0.38% | 1,743.42 | 1,882.32 | 12.77% | 34.46% | | |

## Slide 29
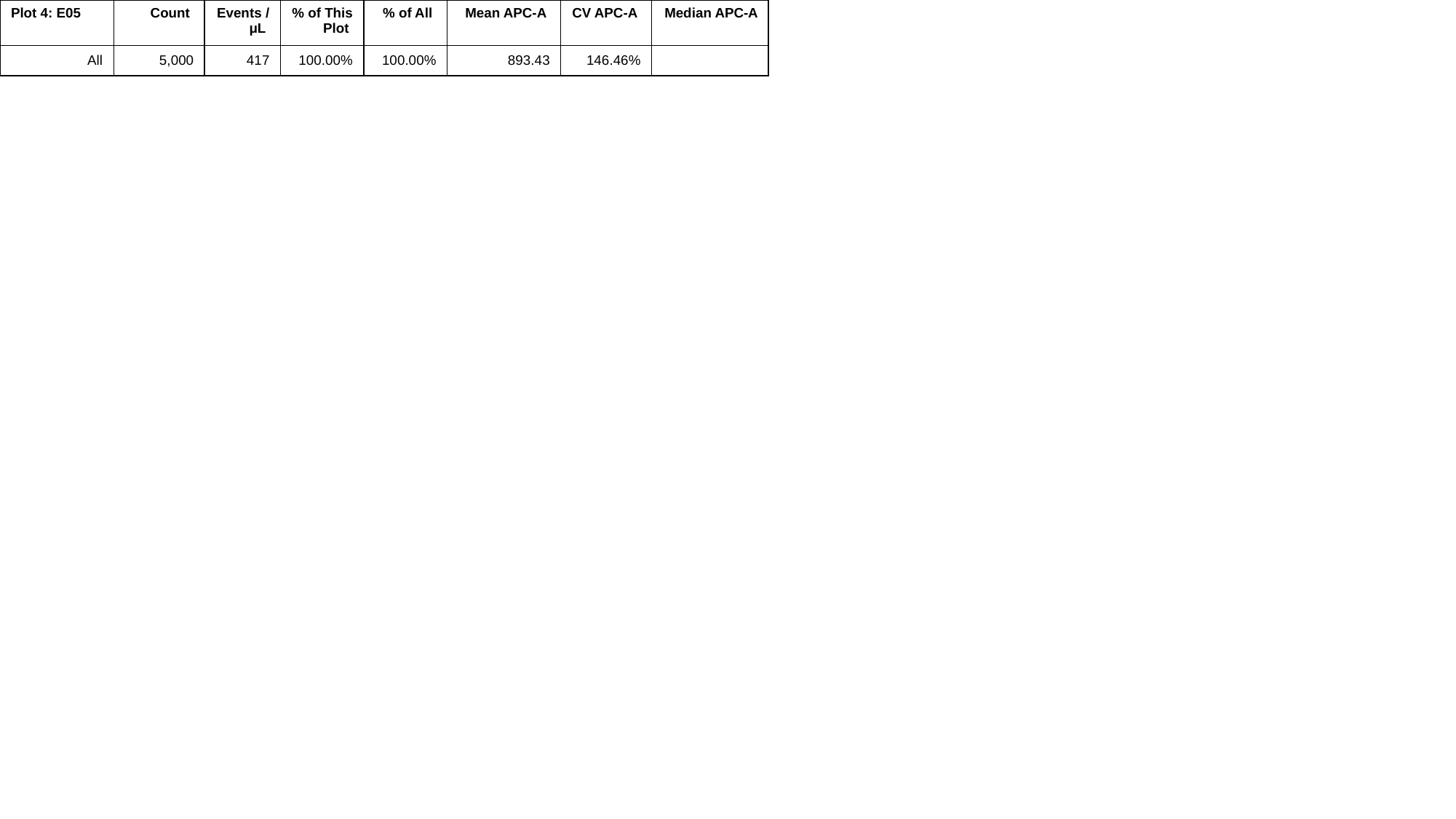

| Plot 4: E05 | Count | Events / μL | % of This Plot | % of All | Mean APC-A | CV APC-A | Median APC-A |
| --- | --- | --- | --- | --- | --- | --- | --- |
| All | 5,000 | 417 | 100.00% | 100.00% | 893.43 | 146.46% | |

## Slide 30
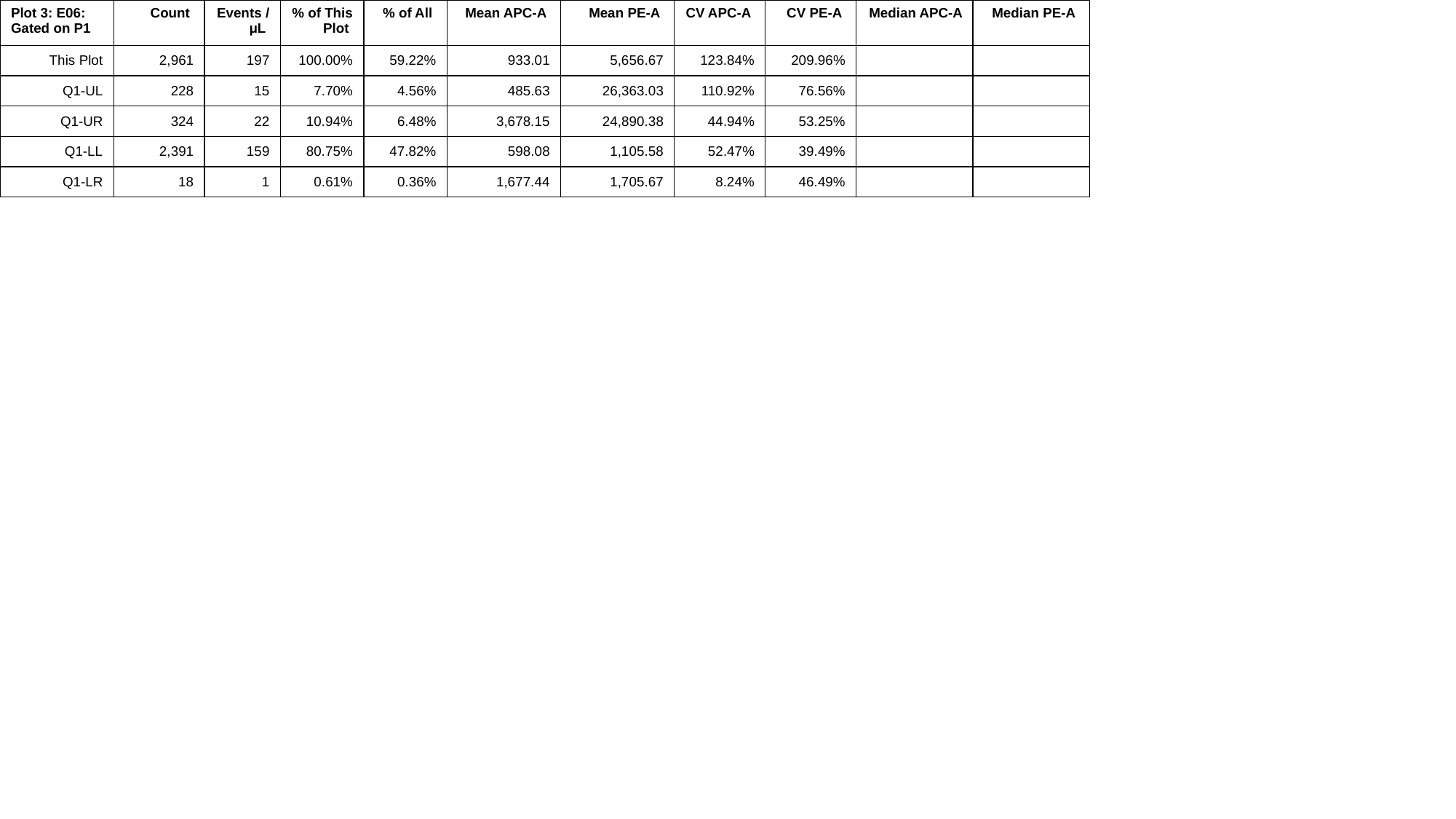

| Plot 3: E06: Gated on P1 | Count | Events / μL | % of This Plot | % of All | Mean APC-A | Mean PE-A | CV APC-A | CV PE-A | Median APC-A | Median PE-A |
| --- | --- | --- | --- | --- | --- | --- | --- | --- | --- | --- |
| This Plot | 2,961 | 197 | 100.00% | 59.22% | 933.01 | 5,656.67 | 123.84% | 209.96% | | |
| Q1-UL | 228 | 15 | 7.70% | 4.56% | 485.63 | 26,363.03 | 110.92% | 76.56% | | |
| Q1-UR | 324 | 22 | 10.94% | 6.48% | 3,678.15 | 24,890.38 | 44.94% | 53.25% | | |
| Q1-LL | 2,391 | 159 | 80.75% | 47.82% | 598.08 | 1,105.58 | 52.47% | 39.49% | | |
| Q1-LR | 18 | 1 | 0.61% | 0.36% | 1,677.44 | 1,705.67 | 8.24% | 46.49% | | |

## Slide 31
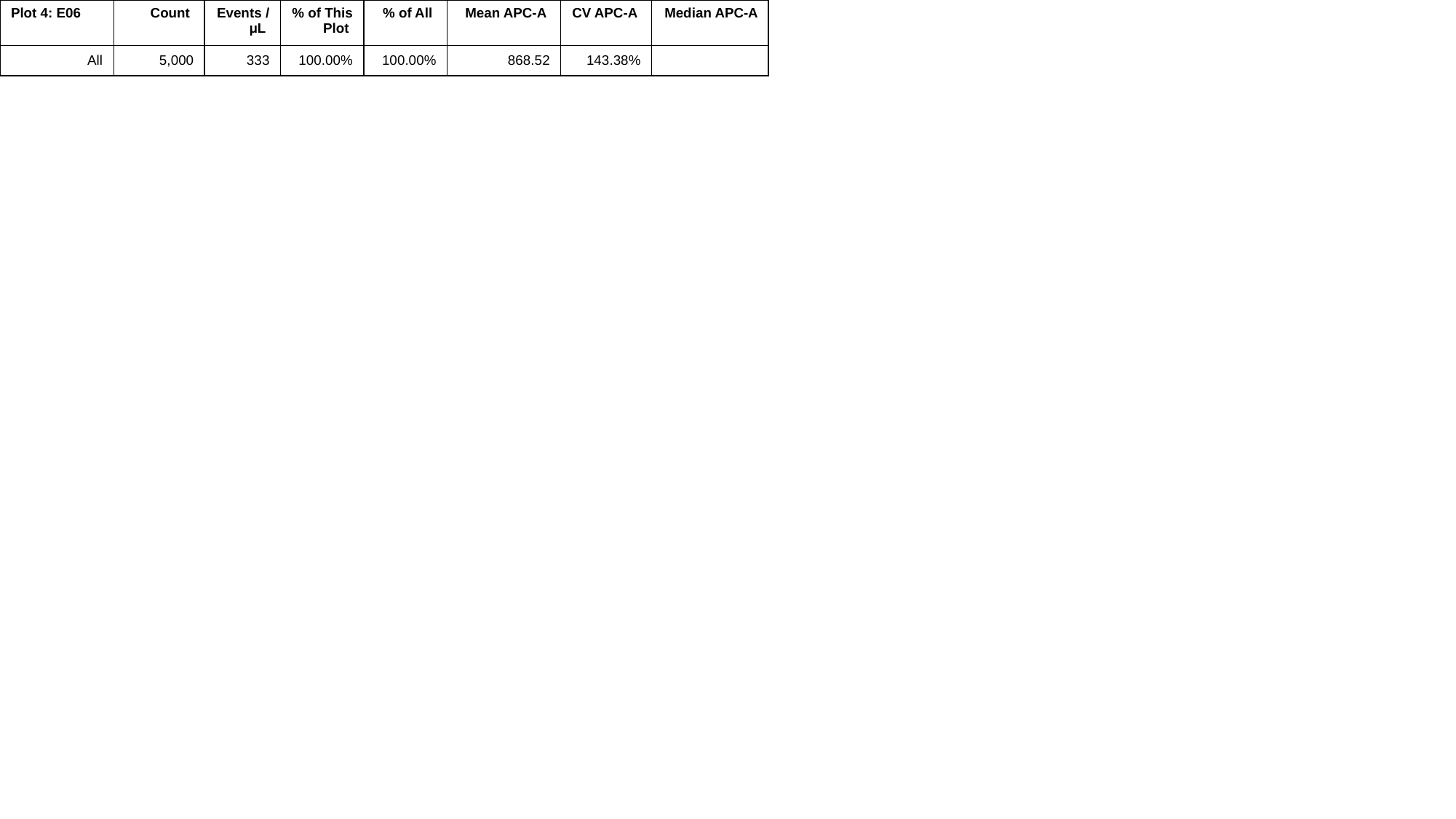

| Plot 4: E06 | Count | Events / μL | % of This Plot | % of All | Mean APC-A | CV APC-A | Median APC-A |
| --- | --- | --- | --- | --- | --- | --- | --- |
| All | 5,000 | 333 | 100.00% | 100.00% | 868.52 | 143.38% | |

## Slide 32
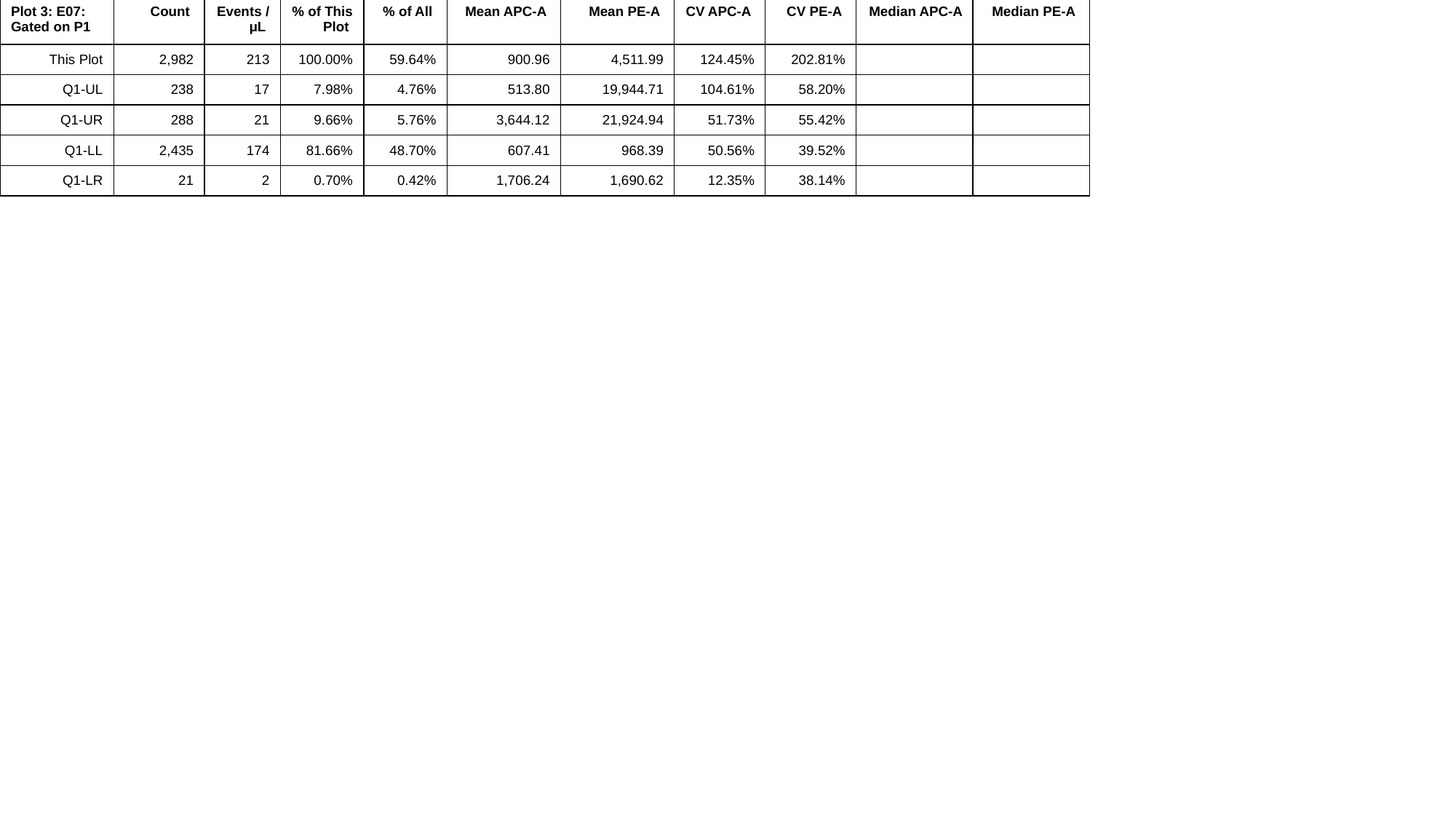

| Plot 3: E07: Gated on P1 | Count | Events / μL | % of This Plot | % of All | Mean APC-A | Mean PE-A | CV APC-A | CV PE-A | Median APC-A | Median PE-A |
| --- | --- | --- | --- | --- | --- | --- | --- | --- | --- | --- |
| This Plot | 2,982 | 213 | 100.00% | 59.64% | 900.96 | 4,511.99 | 124.45% | 202.81% | | |
| Q1-UL | 238 | 17 | 7.98% | 4.76% | 513.80 | 19,944.71 | 104.61% | 58.20% | | |
| Q1-UR | 288 | 21 | 9.66% | 5.76% | 3,644.12 | 21,924.94 | 51.73% | 55.42% | | |
| Q1-LL | 2,435 | 174 | 81.66% | 48.70% | 607.41 | 968.39 | 50.56% | 39.52% | | |
| Q1-LR | 21 | 2 | 0.70% | 0.42% | 1,706.24 | 1,690.62 | 12.35% | 38.14% | | |

## Slide 33
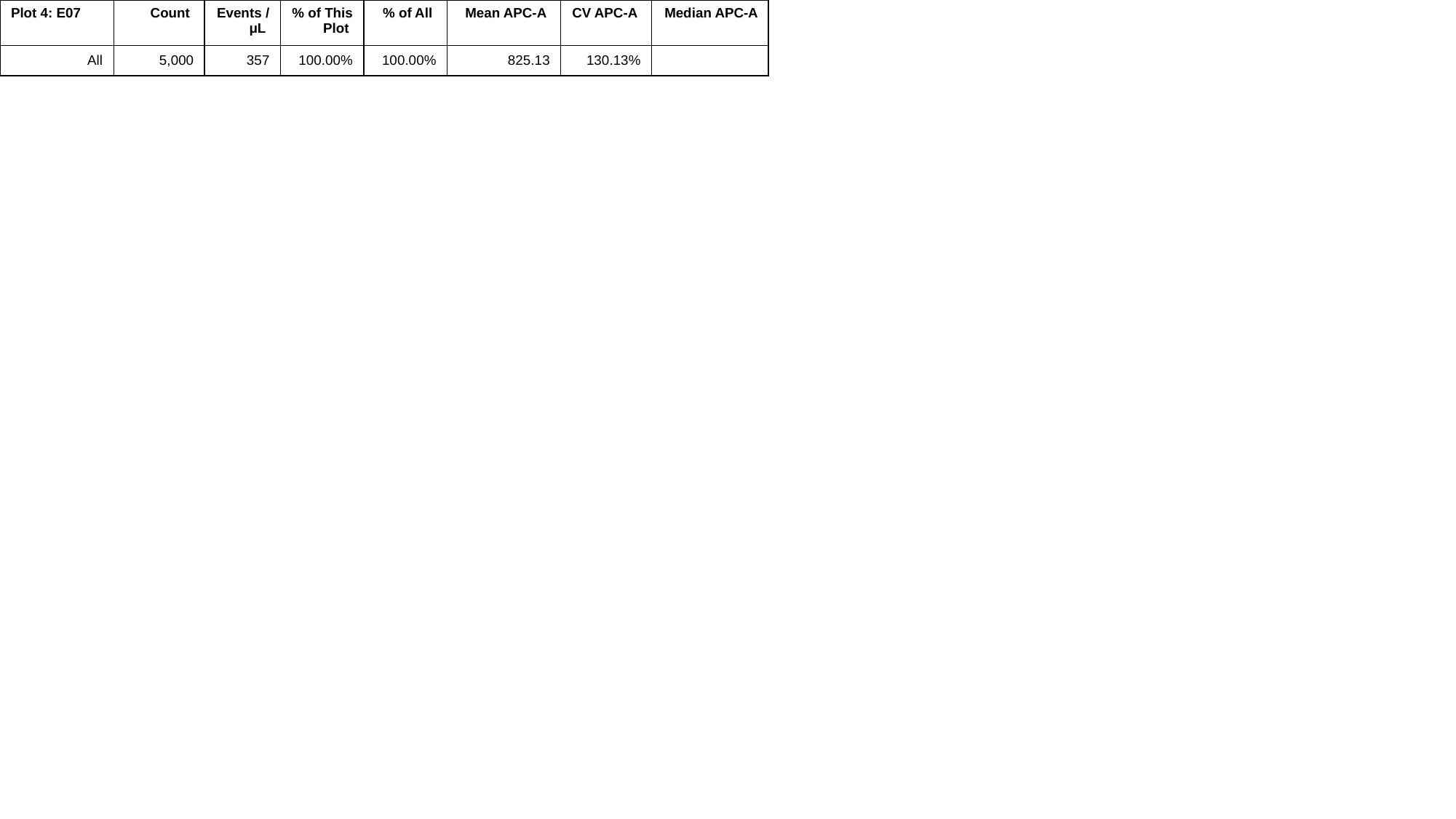

| Plot 4: E07 | Count | Events / μL | % of This Plot | % of All | Mean APC-A | CV APC-A | Median APC-A |
| --- | --- | --- | --- | --- | --- | --- | --- |
| All | 5,000 | 357 | 100.00% | 100.00% | 825.13 | 130.13% | |
