# Supplementary material for: CNN2 silencing inhibits colorectal cancer development through promoting ubiquitination of EGR1
Source: Life Sci Alliance. 2023 May 15;6(7):e202201639. doi: 10.26508/lsa.202201639 (PMC10185810; doi:10.26508/lsa.202201639)
Supplement: Supplementary file 10 [file LSA-2022-01639_SdataF6.3.pdf]

Well Number: B12 Sample ID: B12
File Name: C:/Users/keygg/Desktop/ssx/20210108 RKO DW/2021-01-08_at_06-10-36pm.fcs

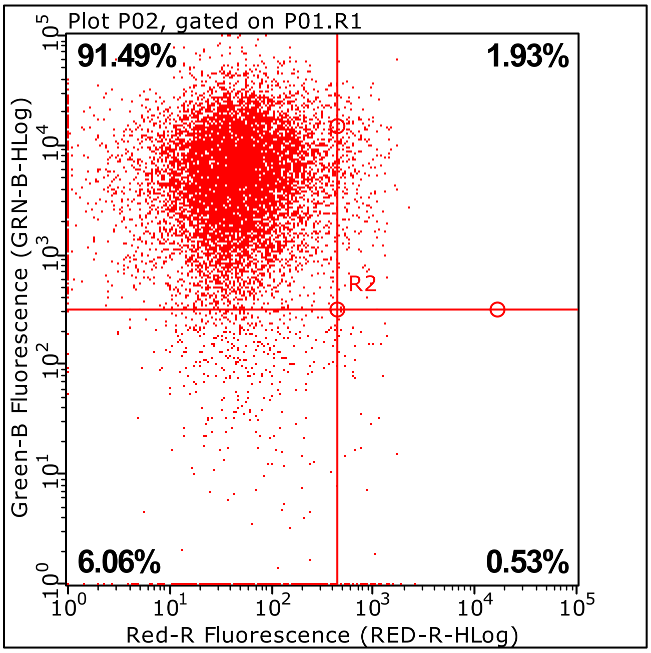


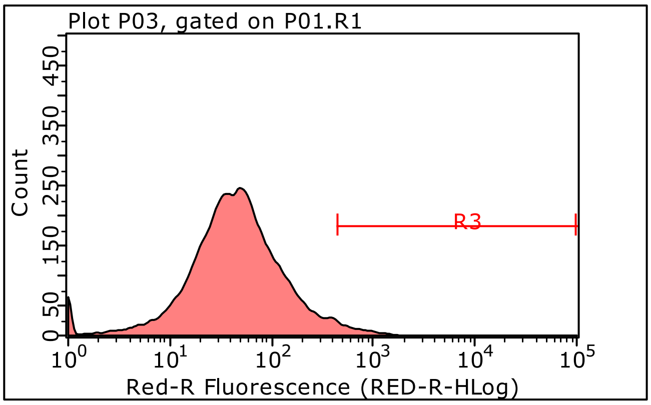

| Well | Sample ID | Date | R2.Percent.UL Percent for R2 gated by P01.R1 (%) | R2.Percent.UR Percent for R2 gated by P01.R1 (%) | R2.Percent.LL Percent for R2 gated by P01.R1 (%) |
|---|---|---|---|---|---|
| B12 | B12 | 01.15.2021 | 91.49 | 1.93 | 6.06 |

| Well | R2.Percent.LR Percent for R2 gated by P01.R1 (%) | R3.Percent Percent for R3 gated by P01.R1 (%) |
|---|---|---|
| B12 | 0.53 | 2.45 |
