# Supplementary material for: CNN2 silencing inhibits colorectal cancer development through promoting ubiquitination of EGR1
Source: Life Sci Alliance. 2023 May 15;6(7):e202201639. doi: 10.26508/lsa.202201639 (PMC10185810; doi:10.26508/lsa.202201639)
Supplement: Supplementary file 15 [file LSA-2022-01639_SdataF6.8.pdf]

Well Number: A11 Sample ID: A11
File Name: C:/Users/keygg/Desktop/ssx/20210108 RKO DW/2021-01-08_at_06-10-36pm.fcs

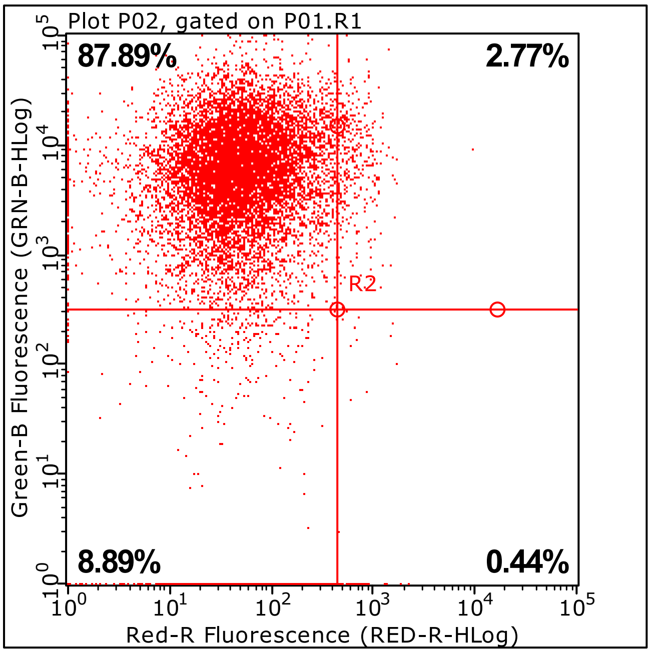

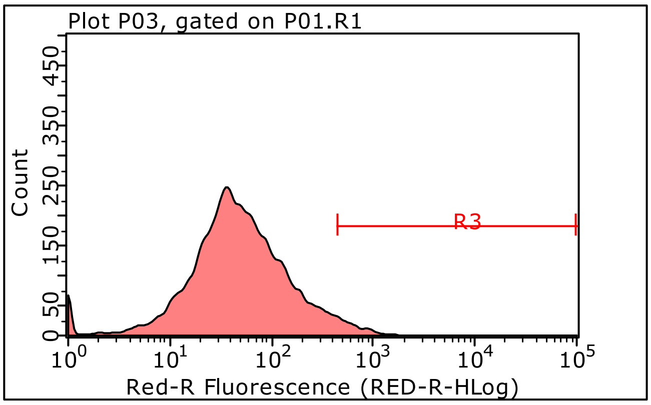

Well	Sample ID	Date	R2.Percent.UL Percent for R2 gated by P01.R1 (%)	R2.Percent.UR Percent for R2 gated by P01.R1 (%)	R2.Percent.LL Percent for R2 gated by P01.R1 (%)
A11	A11	01.15.2021	87.89	2.77	8.89

Well	R2.Percent.LR Percent for R2 gated by P01.R1 (%)	R3.Percent Percent for R3 gated by P01.R1 (%)
A11	0.44	3.22
