# Supplementary material for: CNN2 silencing inhibits colorectal cancer development through promoting ubiquitination of EGR1
Source: Life Sci Alliance. 2023 May 15;6(7):e202201639. doi: 10.26508/lsa.202201639 (PMC10185810; doi:10.26508/lsa.202201639)
Supplement: Supplementary file 20 [file LSA-2022-01639_SdataF6.13.pdf]

Well Number: C11 Sample ID: C11

File Name: C:/Users/keygg/Desktop/ssx/20210108 RKO DW/2021-01-08_at_06-10-36pm.fcs

Plot P02, gated on P01.R1

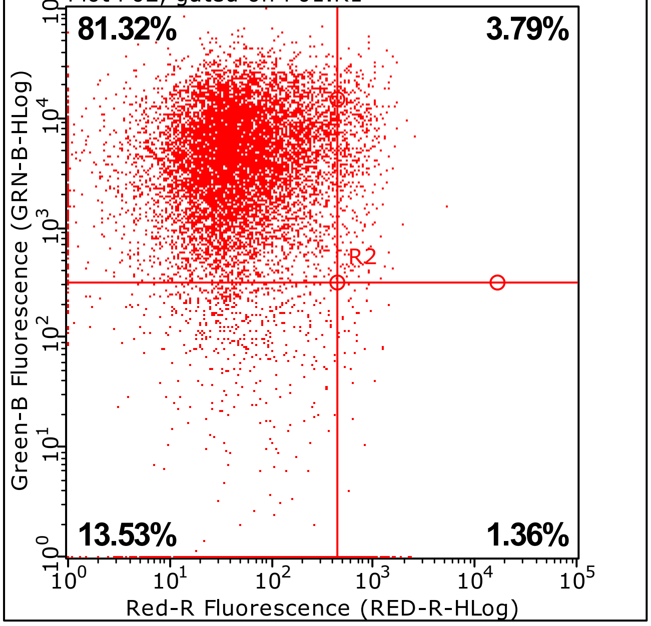

Plot P03, gated on P01.R1

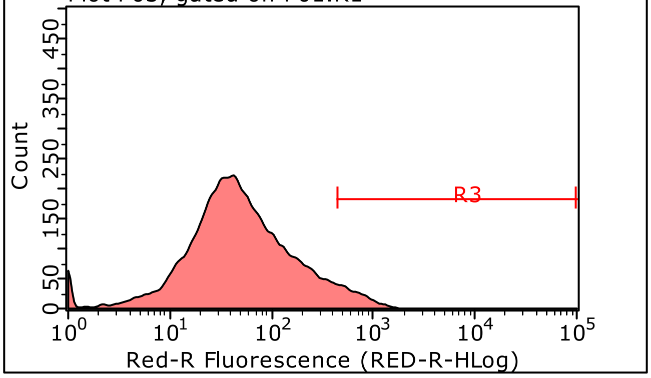

Well	Sample ID	Date	R2.Percent.UL Percent for R2 gated by P01.R1 (%)	R2.Percent.UR Percent for R2 gated by P01.R1 (%)	R2.Percent.LL Percent for R2 gated by P01.R1 (%)
C11	C11	01.15.2021	81.32	3.79	13.53

Well	R2.Percent.LR Percent for R2 gated by P01.R1 (%)	R3.Percent Percent for R3 gated by P01.R1 (%)
C11	1.36	5.13
