# Supplementary material for: CNN2 silencing inhibits colorectal cancer development through promoting ubiquitination of EGR1
Source: Life Sci Alliance. 2023 May 15;6(7):e202201639. doi: 10.26508/lsa.202201639 (PMC10185810; doi:10.26508/lsa.202201639)
Supplement: Supplementary file 21 [file LSA-2022-01639_TableS2.docx]

Table S2 Antibodies used in western blotting and IHC

| Primary antibodies | Dilution in WB | Source species | Company | Catalog No. |
| --- | --- | --- | --- | --- |
| CCNF | 1:1000 | Rabbit | bioss | bs-1686R |
| EGR1 | 1:1000 | Rabbit | bioss | bs-1076R |
| GPX4 | 1:1000 | Rabbit | bioss | bs-3884R |
| CNN2 | 1:1000 | Mouse | biorbyt | orb394893 |
| DYKDDDDK Tag* | 1:50/1:1000 | Rabbit | CST | 14793 |
| YAP1 | 1:50/1:3000 | Rabbit | Abcam | ab52771 |
| Ubiquitin | 1:2000 | Mouse | santa cruz | sc-47721 |
| Primary antibodies | Dilution in IHC | Source species | Company | Catalog No. |
| CNN2 | 1:100 | Mouse | biorbyt | orb394893 |
| EGR1 | 1:200 | Rabbit | bioss | bs-1076R |
| Ki67 | 1/200 | Rabbit | abcam | Ab16667 |
|  |  |  |  |  |
| Secondary antibody | Dilution |  | Company | Catalog No. |
| HRP Goat Anti-Rabbit IgG (WB) | 1:3000 |  | Beyotime | A0208 |
| HRP Goat Anti-Mouse IgG (WB/IHC) | 1:3000/1:200 |  | Beyotime | A0216 |
| HRP Goat Anti-Rabbit IgG (IHC) | 1:200 |  | Abcam | Ab111909 |

* Note: DYKDDDDK Tag binds to same epitope as Sigma's Anti-FLAG® M2 Antibody
