# Supplementary material for: CNN2 silencing inhibits colorectal cancer development through promoting ubiquitination of EGR1
Source: Life Sci Alliance. 2023 May 15;6(7):e202201639. doi: 10.26508/lsa.202201639 (PMC10185810; doi:10.26508/lsa.202201639)
Supplement: Supplementary file 22 [file LSA-2022-01639_TableS3.docx]

Table S3 The target sequences and shRNA sequences

| Gene | No. | Target sequence (5'-3') | shRNA sequences (5'-3') |
| --- | --- | --- | --- |
| CNN2 | Pbr24084-a | CCGCACCTGGATCGAGGGACT | CCGGCCGCACCTGGATCGAGGGACTCTCGAGAGTCCCTCGATCCAGGTGCGGTTTTTG |
| CNN2 | Pbr24084-b | CCGCACCTGGATCGAGGGACT | AATTCAAAAACCGCACCTGGATCGAGGGACTCTCGAGAGTCCCTCGATCCAGGTGCGG |
| CNN2 | Pbr24085-a | CGGCATGAACCCTGTGGACCT | CCGGCGGCATGAACCCTGTGGACCTCTCGAGAGGTCCACAGGGTTCATGCCGTTTTTG |
| CNN2 | Pbr24085-b | CGGCATGAACCCTGTGGACCT | AATTCAAAAACGGCATGAACCCTGTGGACCTCTCGAGAGGTCCACAGGGTTCATGCCG |
| CNN2 | Pbr24085-a | TGGGAACCGACAAGTGTGACA | CCGGTGGGAACCGACAAGTGTGACACTCGAGTGTCACACTTGTCGGTTCCCATTTTTG |
| CNN2 | Pbr24085-b | TGGGAACCGACAAGTGTGACA | AATTCAAAAATGGGAACCGACAAGTGTGACACTCGAGTGTCACACTTGTCGGTTCCCA |
| EGR1 | Pbr10081-a | CATCTCTCTGAACAACGAGAA | CCGGCATCTCTCTGAACAACGAGAACTCGAGTTCTCGTTGTTCAGAGAGATGTTTTTG |
| EGR1 | Pbr10081-b | CATCTCTCTGAACAACGAGAA | AATTCAAAAACATCTCTCTGAACAACGAGAACTCGAGTTCTCGTTGTTCAGAGAGATG |
| EGR1 | Pbr00229-a | CACCATGGACAACTACCCTAA | CCGGCACCATGGACAACTACCCTAACTCGAGTTAGGGTAGTTGTCCATGGTGTTTTTG |
| EGR1 | Pbr00229-b | CACCATGGACAACTACCCTAA | AATTCAAAAACACCATGGACAACTACCCTAACTCGAGTTAGGGTAGTTGTCCATGGTG |
| EGR1 | Pbr00230-a | TGGCCTAGTGAGCATGACCAA | CCGGTGGCCTAGTGAGCATGACCAACTCGAGTTGGTCATGCTCACTAGGCCATTTTTG |
| EGR1 | Pbr00230-b | TGGCCTAGTGAGCATGACCAA | AATTCAAAAATGGCCTAGTGAGCATGACCAACTCGAGTTGGTCATGCTCACTAGGCCA |
| YAP1 | Pbr-12127-a | GAGCTCATTCCTCTCCAGCTT | CCGGGAGCTCATTCCTCTCCAGCTTCTCGAGAAGCTGGAGAGGAATGAGCTCTTTTTG |
| YAP1 | Pbr-12127-b | GAGCTCATTCCTCTCCAGCTT | aattCAAAAAGAGCTCATTCCTCTCCAGCTTCTCGAGAAGCTGGAGAGGAATGAGCTC |
| YAP1 | Pbr-12128-a | CTGGTCAGAGATACTTCTTAA | CCGGCTGGTCAGAGATACTTCTTAACTCGAGTTAAGAAGTATCTCTGACCAGTTTTTG |
| YAP1 | Pbr-12128-b | CTGGTCAGAGATACTTCTTAA | aattCAAAAACTGGTCAGAGATACTTCTTAACTCGAGTTAAGAAGTATCTCTGACCAG |
| YAP1 | Pbr-12129-a | AACCGTTTCCCAGACTACCTT | CCGGAACCGTTTCCCAGACTACCTTCTCGAGAAGGTAGTCTGGGAAACGGTTTTTTTG |
| YAP1 | Pbr-12129-b | AACCGTTTCCCAGACTACCTT | aattCAAAAAAACCGTTTCCCAGACTACCTTCTCGAGAAGGTAGTCTGGGAAACGGTT |
