# Supplementary material for: CNN2 silencing inhibits colorectal cancer development through promoting ubiquitination of EGR1
Source: Life Sci Alliance. 2023 May 15;6(7):e202201639. doi: 10.26508/lsa.202201639 (PMC10185810; doi:10.26508/lsa.202201639)
Supplement: Supplementary file 23 [file LSA-2022-01639_TableS4.docx]

Table S4 Primers used in qPCR

| Gene | Forward primer sequence (5’-3’) | Reverse primer sequence (5’-3’) |
| --- | --- | --- |
| GAPDH | TGACTTCAACAGCGACACCCA | CACCCTGTTGCTGTAGCCAAA |
| CNN2 | GGGCCTGAAGGATGGAACTA | TTCTGCATGGAGCGGTTGA |
| ABL1 | TTCTGATGGCAAGCTCTACGT | TTTGGGGCTGGATAATGGA |
| CDCA5 | CCGAGCATCCTCCCTGAAAT | CATGGGCCACGATCCTCTTTA |
| GNB2 | ACAGTGGGTTTTGCTGGACA | GCACGTCCCACAGCTTGATA |
| NDUFA3 | GCGGAGACAAAGATGGCT | ACGACGAAGGACACGACC |
| PLCD3 | TCAACGAGACAGCCAAGC | AGATGAAGTAGTGGGCAAGG |
| RPTOR | TACGACTGCTCCAATGCTG | AGAGGGTGATTTGGGTTGA |
| LIMK1 | CAGACTGCTTCAGGTGTTGTGA | CAGTCCCTTGGTGATTTGCT |
| GRIN2D | TTCCACCGAGCAACAGCTT | ACGAGTGGTCACGGCTACAA |
| ULK1 | TGCACAGCAAAGGCATCAT | AGCCGAAGTCAGCGATCTTG |
| EGR1 | CACCTGACCGCAGAGTCTTTT | TGGTTTGGCTGGGGTAACTG |
| GRINA | ACTTCCCTGCCACCAACT | TTCACCTCCGCAACAAAA |
| PRKCD | CGTTTCTCCTGGTGGTTGGT | TAGGAGTTGAAGGCGATGCG |
| APOA2 | TTGAAGGAGCTTTGGTTCGG | CATAGTCAGTCACGGTCTGGAAG |
| GNB2 | ACAGTGGGTTTTGCTGGACA | GCACGTCCCACAGCTTGATA |
| RASGRP1 | CCTTCAAAGCCACCAGTA | CAGAATCCACCATCCTCT |
| JAK1 | CAAGCAGGACAACAAGAAAATG | GGTGCAGAGGTAATGATGGG |
| FAM117B | CAGAGCCGAAGCTCGCCGGAGAAGA | CAGGGAGGAAGTGCGTCGGATAATACTGG |
| CCNF | AAACGGAAGCGGGAGAACA | TCGCCTTCATAGCCAGAGCA |
| GPX4 | CCAGTGAGGCAAGACCGAAGTA | TTACACGGGAAGGCCAGGAT |
| FAS | GGAAAAGAGGCCGTGGGG | GGACTGCGGACTAGGTGTG |
|  |  |  |
